# Biocatalyst collection and heterologous expression of sesquiterpene synthases from basidiomycetous fungi: Discovery of a novel sesquiterpene hydrocarbon

**DOI:** 10.1111/1751-7915.14204

**Published:** 2022-12-28

**Authors:** Natsuki Masunaga, Takuya Kitaoka, Hirofumi Ichinose

**Affiliations:** ^1^ Faculty of Agriculture Kyushu University Fukuoka Japan

## Abstract

Basidiomycetes produce a wide variety of sesquiterpenoids, which attract significant interest in pharmaceutical and industrial applications. Structural diversification of sesquiterpenoids is performed by sesquiterpene synthases (STSs), which produce a wide array of backbone structures; therefore, functional characterization and increased biocatalyst collection of STSs are important for expanding scientific knowledge and meeting the needs of advanced biotechnology. Gene identification and functional annotation of STSs from the basidiomycetous fungi *Agaricus bisporus*, *Auriscalpium vulgare*, *Lepista nuda*, *Pleurotus ostreatus* and *Trametes versicolor* were conducted. Through these investigations, the catalytic functions of 30 STSs were revealed using recombinant enzymes heterologously expressed in *Saccharomyces cerevisiae*. Furthermore, the unique function of an STS from *P. ostreatus*, PoSTS‐06, was revealed to be the production of a novel sesquiterpene hydrocarbon that we named pleostene. The absolute structure of pleostene was determined by NMR spectroscopy and X‐ray crystallography using the crystalline sponge method.

## Graphical abstract

Gene identification of basidiomycetous sesquiterpene synthases. Functional characterization of basidiomycetous sesquiterpene synthases. Discovery of the novel sesquiterpene hydrocarbon pleostene.

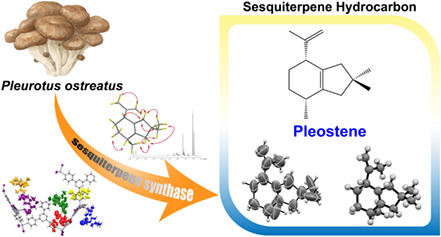

## INTRODUCTION

Terpenoids are arguably the largest class of natural products, showing a fascinating array of structural diversity. Many researchers have devoted much attention to discovering and using their potential in pharmaceutical and/or industrial fields. A wide variety of terpenoids have been isolated from nature and, to improve our understanding, their bioactive properties and the mechanisms of their biosynthesis have attracted significant interest (Ludwiczuk et al., 2017). A remarkable diversity of backbone structures is found among the sesquiterpenoids, which possess three isoprene units (Fraga, 2013; Kramer & Abraham, 2012; Quan et al., 2021; Quin et al., 2014). Biochemical studies on sesquiterpene metabolism have established a general scheme in which naturally occurring sesquiterpenoids are produced by concomitant actions of sesquiterpene synthases (STS) and terpene‐decoration enzymes, such as cytochrome P450 monooxygenases (Christianson, 2006; Quan et al., 2021; Weitzel & Simonsen, 2015). In the early stage of sesquiterpene biosynthesis, STSs play fundamental roles in the diversification of the backbone structure by catalysing cyclization of the common precursor farnesyl pyrophosphate (FPP) and/or its isomer nerolidyl pyrophosphate (NPP). The highly complex cyclization events give rise to a fascinating diversity of backbone structures of sesquiterpene molecules (Miller & Allemann, 2012), which are an important source of lead compounds in drug development (Urabe et al., 2015). Thus, wide‐ranging investigations of STSs are challenging but are a gateway to the development of potentially valuable compounds and their downstream applications.

Fungi inhabit a broad range of environments and produce an enormous array of secondary metabolites. A series of bioactive natural products such as illudin M and S (cytotoxic activity) and pleuromutilin (antibacterial activity) have been isolated from basidiomycetous fungi; thus, fungi are a rich source of interesting natural products that have pharmaceutical and agricultural relevance (Chen & Liu, 2017; Gressler et al., 2021; Keller, 2021; Nord et al., 2015; Sandargo et al., 2019; Yamane et al., 2017). In the fungal kingdom, basidiomycetes produce a wide variety of sesquiterpenoids with an extensive repertoire of backbone structures (Chen & Liu, 2017; Fraga, 2013; Kramer & Abraham, 2012). For instance, several sesquiterpenoids possessing rare/unique/novel backbone skeletons have been isolated and characterized from basidiomycetous fungi (Luo et al., 2022; Nakashima et al., 2019; Niu et al., 2020). Genome sequences of many fungi have been determined and released through the 1000 Fungal Genomes Project (Grigoriev et al., 2014), leading to a new research paradigm associated with the functionomics of enzymes. This stimulating project enables us to access genomic databases for 500 basidiomycetous fungi (thus far). Thereby, tremendous numbers of STS candidates can be rationally and rapidly discovered. However, it remains challenging to informatically elucidate the catalytic functions of fungal STSs, even in the postgenomic era.

In these circumstances, we aimed to increase functional information about basidiomycetous STSs isolated from a series of basidiomycetes from several families and genera. Herein, we describe the catalytic functions of 30 STSs gathered from five basidiomycetous fungi: *Agaricus* (*Ag*.) *bisporus*, *Auriscalpium* (*Au*.) *vulgare*, *Lepista nuda*, *Pleurotus* (*P*.) *ostreatus* and *Trametes versicolor*. On the basis of functional studies, several STSs that produce unique backbone structures of sesquiterpenoids were identified. Notably, our study has also led to the discovery of a novel sesquiterpene hydrocarbon, which we named pleostene.

## EXPERIMENTAL PROCEDURES

### Chemicals

Yeast nitrogen base without amino acids was purchased from Formedium. The dropout supplement was prepared from amino acids (Peptide Institute) and nucleotides (FUJIFILM Wako Chemicals). Custom‐synthesized oligonucleotide primers were obtained from Sigma‐Aldrich Japan. All other chemicals were of reagent grade. Deionized water was obtained from a Barnstead™ Smart2Pure™ Water Purification System (Thermo Fisher Scientific).

### Gene identification of basidiomycetous STSs

Possible coding sequences of STSs were found in the US Department of Energy Joint Genome Initiative database (http://genome.jgi.doe.gov/Phchr1/Phchr1.home.html) using the basic local alignment search tool (BLAST) with known STSs from basidiomycetes as the queries (Agger et al., 2009; Ichinose et al., 2022; Ichinose & Kitaoka, 2018; Wawrzyn et al., 2012). The genomic databases used were as follows: *Ag. bisporus* H97 (v2.0) (Morin et al., 2012), *Au. vulgare* FP105234‐Sp (v1.0) (Looney et al., 2022), *L. nuda* CBS 247.69 (v1.0) (Ruiz‐Dueñas et al., 2021), *P. ostreatus* PC15 (v2.0) (Alfaro et al., 2016; Castanera et al., 2016; Riley et al., 2014) and *T. versicolor* MB281625 (v1.0) (Floudas et al., 2012). Annotation accuracy was evaluated by comparing overall sequence similarity against known STSs and identifying the signature aspartate‐rich and NSE/DTE motifs of STSs.

### Isolation of cDNAs encoding STSs

The basidiomycetous fungi *Ag. bisporus* (NBRC 31861), *Au. vulgare* (NBRC 30159), *L. nuda* (NBRC 30380), *P. ostreatus* (NBRC 104981) and *T. versicolor* (NBRC 30340) were grown from hyphal inocula at 28°C in stationary culture (20 ml of medium) in aerobic conditions. The medium (pH 6.0) used in this study contained, as previously described, 1% glucose and 1.2 or 12 mM ammonium tartrate as the carbon and nitrogen sources, respectively (Kirk et al., 1978; Nazir et al., 2010). Total RNA was isolated individually from 5‐, 7‐, 10‐ and 12‐day‐old mycelia of each fungus and applied for cDNA amplification by reverse transcription polymerase chain reaction (RT‐PCR). Primer sequences (Table S3) and RT‐PCR conditions are provided in the Supporting Information. Amplified gene fragments were cloned into the plasmid pBluescript II KS(−) and sequenced using an automated DNA Sequencer (ABI3500xl; Applied Biosystems). The nucleotide and amino acid sequences of isolated STSs were deposited in the DNA Data Bank of Japan (see Table S1).

### Heterologous expression and functional characterization of STSs

The coding sequence of each STS was re‐amplified by PCR from the cloning vector. PCR conditions and primer sequences (Table S4) are provided in the Supporting Information. The resulting gene fragments were ligated into the yeast expression vector pGYRG, linearized with *Psh*AI/*Spe*I, using the In‐Fusion HD cloning kit (TaKaRa Bio USA) as described previously (Ichinose et al., 2022; Ichinose & Kitaoka, 2018). *Saccharomyces cerevisiae* (AH22) was transformed with the expression plasmid using the lithium acetate method (Ichinose et al., 2022; Ito et al., 1983). Positive transformants were isolated by auxotrophic selection using synthetic dextrose agar plates (2% glucose, 0.67% yeast nitrogen base without amino acids and 1.5% agar) supplemented with l‐histidine (20 mg L^−1^). A fresh transformant was inoculated into 3 ml of synthetic dextrose liquid medium containing 8% glucose, 2.68% yeast nitrogen base without amino acids and 0.1% dropout supplement without l‐leucine (SDL/−Leu) in a 15‐ml conical tube and grown for 2 days in a Micro Bio Shaker (TAITEC) at 28°C. The composition of the dropout supplement is described in the Supporting Information. After preincubation, 0.1 ml of the culture was seeded into 20 ml SDL/−Leu medium in a 100‐ml baffled flask, sealed with cling film and incubated at 28°C with shaking (180 rpm). After incubation for 48 h, metabolites in the culture headspace were sampled by monolithic silica adsorbent (Mono Trap® GL Sciences) suspended by a cotton thread in the headspace for 3 h and eluted with 300 μl of chloroform. Metabolites in the culture medium were recovered by solid‐phase extraction using an InertSep™ RP‐C18 cartridge (GL Sciences Inc.) and eluted with 300 μl of acetone (Ichinose et al., 2022). Gas chromatography–mass spectrometry (GC–MS) analysis was conducted on a Shimadzu QP2010 SE system. Separation of metabolites was performed using a 30‐m fused silica capillary column (SLB®‐5 ms; Supelco) with an injection port temperature of 280°C and helium as the carrier gas. Mass spectra were observed following electron impact ionization at 70 eV. The oven temperature was programmed from 50 to 350°C with a gradient of 3°C min^−1^. The metabolites were putatively identified by comparing their mass spectra and retention indices with those of known sesquiterpenoids using the FFNSC3 and NIST14 mass spectral library in the software GCMSsolution (Shimadzu) and the terpenoids library in MassFinder (Dr. Hochmuth Scientific Consulting).

### Purification of pleostene for structural analysis

For the structural determination of pleostene, which is a sesquiterpene hydrocarbon produced by the enzyme PoSTS‐06 from *P. ostreatus*, an expression plasmid was engineered based on pALLF (Figure S1). Briefly, the expression plasmid was designed to allow the overproduction of FPP associated with upregulated farnesyl pyrophosphate synthetase (ERG20 from *S. cerevisiae*) and truncated 3‐hydroxy‐3‐methylglutaryl‐CoA reductase (tHMG1 from *S. cerevisiae*) (Scalcinati et al., 2012). The experimental procedure and primer sequences (Table S5) for the construction of the pALLF‐based expression plasmid are detailed in the Supporting Information. For heterologous expression, *S. cerevisiae* (InvSC1) harbouring the expression plasmid was grown in synthetic dextrose liquid medium (20 ml) consisting of 8% glucose, 2.68% yeast nitrogen base without amino acids and 0.1% dropout supplement without uracil (SDL/−Ura) in 100‐ml Erlenmeyer flasks (28°C, 160 rpm) until the early stationary phase. Then, 2.0 ml of the culture was transferred to 1 L of SDL/−Ura medium in a 2‐L baffled flask in which monolithic silica adsorbents (20 pieces of Mono Trap®) were suspended by cotton thread in the headspace. The culture medium was maintained at 28°C and stirred using a magnetic stirrer (450 rpm) for cell growth and metabolic production of sesquiterpene. After 48 h of incubation, volatile metabolites trapped on the monolithic silica adsorbents were eluted with chloroform and purified by silica gel column chromatography using Wakogel C‐300 (Wako Pure Chemical) with hexane. Finally, 50 mg of pure product was obtained from 8 L of culture (i.e. eight 1‐L cultures in 2‐L baffled flasks). The purified metabolite was subjected to structural determination using nuclear magnetic resonance (NMR) spectroscopy and X‐ray crystallography using the crystalline sponge method.

### Structural analysis of pleostene

Optical rotations were recorded on a JASCO P‐2200 digital polarimeter (Jasco Corp.) at 20°C. ^1^H‐ and ^13^C‐NMR, distortionless enhancement by polarization transfer (DEPT), ^1^H–^1^H correlation spectroscopy (COSY), heteronuclear two‐bond correlation (H2BC), heteronuclear single quantum coherence (HSQC), heteronuclear multiple bond coherence (HMBC) and nuclear Overhauser effect spectroscopy (NOESY) spectra were determined with a JNM‐ECZ400 instrument (JEOL) and analysed using Delta NMR software (JEOL). Chemical shifts are expressed as parts per million downfield from the internal standard tetramethylsilane. Samples were dissolved in deuterated chloroform. Meanwhile, crystalline sponge analysis was conducted by Mitsui Chemical Analysis & Consulting Service, Inc. as described previously (Inokuma et al., 2013, 2014). A single crystal of the porous [(ZnI_2_)_3_(tpt)_2_(solvent)_*x*_]_*n*_ complex (tpt, tris(4‐pyridyl)‐1,3,5‐triazine) was used as a crystalline sponge (Supporting Information). Single crystal X‐ray diffractometry (Bruker AXS D8 VENTURE) was performed using a CuKα X‐ray source. The sample crystal was cooled to 93 K using a cold nitrogen stream. Crystallographic data are described in the Supporting Information. The crystallographic information file (CIF) has been deposited at the Cambridge Crystallographic Data Center (2203322).

## RESULTS AND DISCUSSION

Sequence information for basidiomycetous STSs has increased exponentially because of fungal genome projects, and numerous gene candidates encoding STSs can now be discovered from genomic databases. Nevertheless, it is still challenging to rationally explore their catalytic functions by informatic studies, implying that functional annotations through experimental trials are still fundamental for better understanding and application of STSs. Therefore, here, we aimed to increase functional information about basidiomycetous STSs by using recombinant enzymes. Five fungal species were employed in this study as experimental resources to study the molecular and functional diversity of STSs. Fungal strains were selected based on the following criteria: the fungi are/were (i) often used for basic and application studies; (ii) classified into different families and genera; (iii) able to grow in synthetic culture; (iv) isolated in Japan and deposited in the NBRC (National Institute of Technology and Evaluation Biological Resource Center) but found globally; and (v) available in a public genome database. Thus, the following five basidiomycetous fungi were selected: *Ag. bisporus*, *Au. vulgare*, *L. nuda*, *P. ostreatus* and *T. versicolor* (Table 1).

**TABLE 1:** Summary of numbers of STSs from basidiomycetous fungi.

Organism (fungal family)	Gene candidate^a^ ^,^ ^d^	Isolated gene^b^	Functionally expressed^c^
*Ag. bisporus* (*Agaricaceae*)	9	4	3
*Au. vulgare* (*Auriscalpiaceae*)	14	9	5
*L. nuda* (*Tricholomataceae*)	27^d^	14	8
*P. ostreatus* (*Pleurotaceae*)	17^d^	8	7
*T. versicolor* (*Polyporaceae*)	19	11	7
Total	86	46	30

^a^ Number of genes found in genome database.

^b^ Number of STSs isolated as full‐length cDNAs.

^c^ Number of STSs producing metabolite(s) in *S. cerevisiae*.

^d^ Putative gene fragments (LnSTS‐16, LnSTS‐17, LnSTS‐22, and PoSTS‐15) are also counted.

### Gene identification and isolation of STSs from basidiomycetes

**FIGURE 1:**
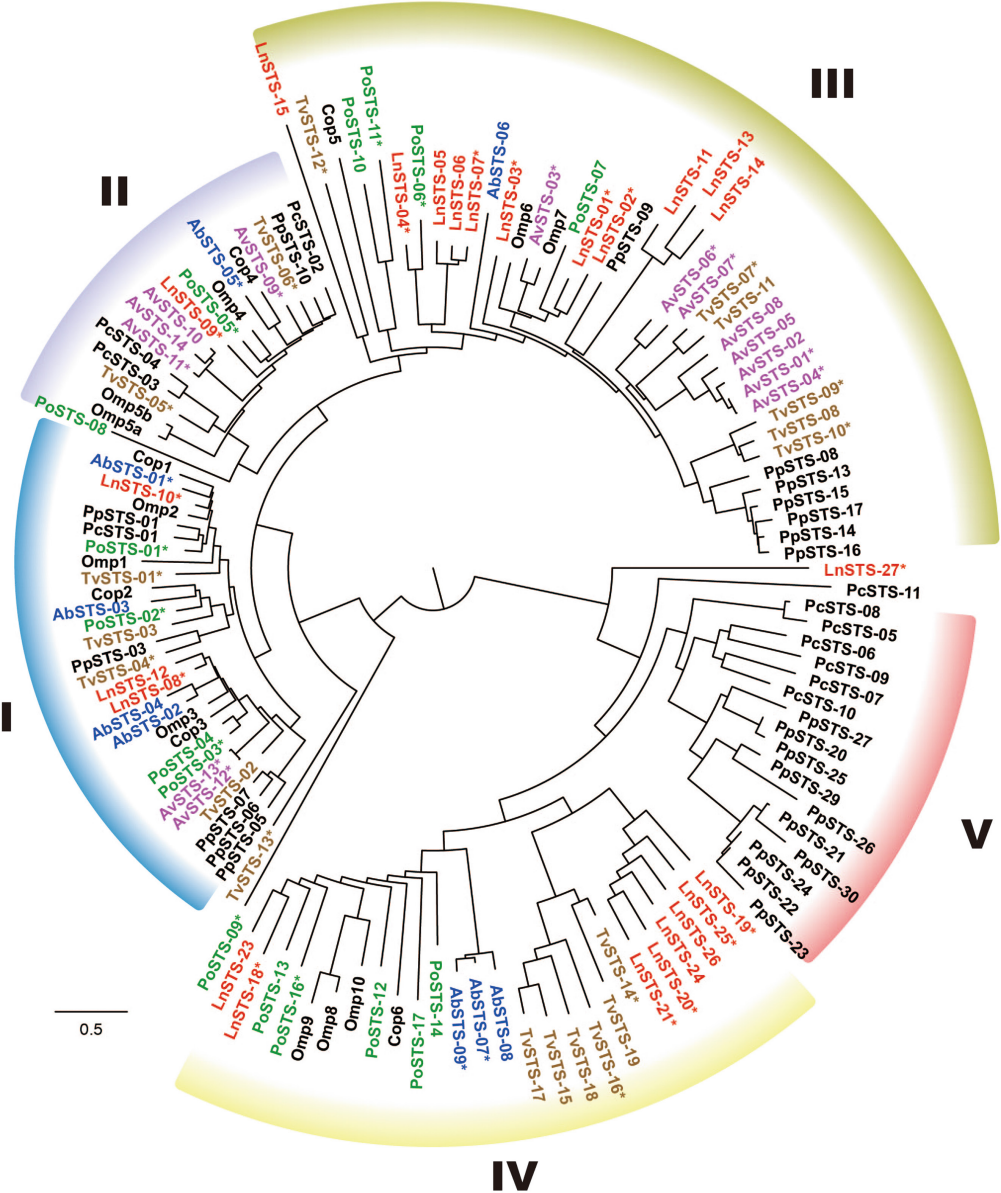
Phylogenetic tree of basidiomycetous sesquiterpene synthases (STSs). cDNAs isolated from basidiomycetous fungi in this study are indicated by asterisks. The phylogenetic tree includes known basidiomycetous STSs (Table S2).

Possible coding sequences of STSs from *Ag. bisporus* (AbSTS), *Au. vulgare* (AvSTS), *L. nuda* (LnSTS), *P. ostreatus* (PoSTS) and *T. versicolor* (TvSTS) were searched for in their genome databases using BLAST. Bioinformatic survey resulted in the identification of 86 possible sequences, which showed overall similarity to known STSs (Table 1). The candidates were arbitrary designated AbSTS‐01 to ‐09, AvSTS‐01 to ‐14, LnSTS‐01 to ‐27, PoSTS‐01 to ‐17 and TvSTS‐01 to 19 (Table S1). Four candidates (LnSTS‐16, LnSTS‐17, LnSTS‐22 and PoSTS‐15) showed partial sequence similarity to known STSs but lacked appropriate start codons, suggesting they are functionally silent. Therefore, we omitted these four candidates from further investigations. A phylogenetic tree of the identified STSs was constructed with known basidiomycetous STSs (Figure 1). As suggested in earlier reports, basidiomycetous STSs form five distinct clades (Ichinose et al., 2022; Ichinose & Kitaoka, 2018; Wawrzyn et al., 2012) and the STSs identified in the present work appeared in clades I to IV (Figure 1). Interestingly, however, clade V was exclusively populated by STSs from *Postia* (*Rhodonia*) *placenta* and *Phanerochaete chrysosporium*, which were identified in our previous studies (Ichinose et al., 2022; Ichinose & Kitaoka, 2018), suggesting that STSs classified in clade V have emerged and diversified in certain fungal species. By contrast, STSs in clades I to IV seem to be widely distributed in basidiomycetes.

Then, cDNA isolation for all possible STSs identified in this study was performed. Previously, we demonstrated that the basidiomycetous fungi *P. placenta* and *P. chrysosporium* express a number of STSs when their mycelia are grown in synthetic culture medium (Ichinose et al., 2022; Ichinose & Kitaoka, 2018). Therefore, *Ag. bisporus*, *Au. vulgare*, *L. nuda*, *P. ostreatus* and *T. versicolor* were grown in the synthetic culture medium and total RNAs were extracted from the fungal mycelia. Using RT‐PCR, 46 cDNAs of candidate STSs in which a mature open reading frame was encoded were isolated (Table 1; see also Table S1). Single nucleotide polymorphisms resulting in amino acid substitutions when compared with the genomic database were often found in the isolated cDNAs. Polymorphic variations resulting in one or two amino acid insertion(s)/deletion(s) were also found in AvSTS‐07, AvSTS‐12, AvSTS‐13, LnSTS‐10 and PoSTS‐11. However, the mutations seemed to have no detrimental effects on the catalytic performance of the STSs. Polymorphic differences might be common in basidiomycetes (Bezmenova et al., 2020; Ide et al., 2012). Several candidates were amplified as immature cDNAs whose open reading frame was shifted by intronic sequences, which were incorrectly spliced; filamentous fungi may sometimes allow a deleterious splicing event (Ide et al., 2012; Nazir et al., 2010; Permana et al., 2022). In addition, 31 candidate genes were not amplified by RT‐PCR. Although further investigations are required, it is possible that several STSs were transcriptionally regulated (Grosse et al., 2019) and probably not expressed in the experimental conditions used in this study.

The signature sequences in the isolated cDNAs were then evaluated (Figure S2). The aspartate‐rich region and the NSE/DTE motif play vital roles in coordinating Mg^2+^ to facilitate ionization of FPP/NPP in the active site of STSs (Aaron & Christianson, 2010). Amino acid sequence for the NSE/DTE motif was conserved in 44 of the STSs. Meanwhile, a D(D/E/N)xxD sequence frequently appeared in the aspartate‐rich region of STSs, and otherwise, the sequence DDxxx was encoded in the appropriate position. The sequence features of the aspartate‐rich regions were similar to those of known STS sequences (Ichinose et al., 2022; Ichinose & Kitaoka, 2018). In PoSTS‐11, amino acid residues in the aspartate‐rich region were non‐canonically substituted (two glutamic acids, but not aspartic acid residues, were found). Two of the putative STSs (PoSTS‐09 and TvSTS‐13) lacked both aspartate‐rich regions and the NSE/DTE motifs in their sequences, suggesting they might be functionally inactive. Nucleotide and deduced amino acid sequences of the isolated cDNAs are provided in the Supporting Information.

### Heterologous expression of STSs in *S. cerevisiae*

To investigate the catalytic functions of the STSs, we constructed expression plasmids using the pGYRG vector, which allows constitutive expression of a heterologous gene under the control of the glyceraldehyde‐3‐phosphate dehydrogenase promoter/terminator in *S. cerevisiae* (Nazir et al., 2011; Sakaki et al., 1992). The isolated cDNAs of 46 STSs were harboured in the expression cassette of pGYRG and, respectively, transformed into *S. cerevisiae*. The yeast transformants were grown in SDL medium and metabolic products, which accumulated in the culture headspace and medium were analysed by GC–MS. Through the metabolic study, 30 of the STSs were confirmed to be functionally expressed in *S. cerevisiae*, generating catalytic products. By comparing mass spectra and retention indices with reference data, various sesquiterpene hydrocarbon and alcohol products were putatively identified (Table 2, see also Figures S3 and S4).

**TABLE 2:** List of products putatively identified by GC–MS analysis.

Origin	Enzyme	Phylogenetic clade	Products	
			Sesquiterpene hydrocarbon	Sesquiterpene alcohol
*Ag. bisporus*	AbSTS‐05	II	Cadina‐1,4‐diene (RI, 1537; SI, 97) Cadina‐1(6),4‐diene (RI, 1476; SI, 97) δ‐Cadinene (RI, 1523; SI, 97) Zonarene (RI, 1528; SI, 96)	Epicubenol (RI, 1633; SI, 96) Cadin‐4‐en‐10‐ol (RI, 1661; SI, 92)
	AbSTS‐07	IV	δ‐Cadinene (RI, 1523; SI, 97) Epizonarene (RI, 1500; SI, 93)	
	AbSTS‐09	IV	(*Z*)‐α‐bisabolene (RI, 1503; SI, 93)	
*Au. vulgare*	AvSTS‐01	III		Unknown compound (RI, 1614; SI, ND)
	AvSTS‐03	III	Δ6‐Protoilludene (RI, 1382; SI, 96)	
	AvSTS‐06	III		(*E*)‐nerolidol (RI, 1562; SI, 96)
	AvSTS‐07	III		(*E*)‐nerolidol (RI, 1561; SI, 96)
	AvSTS‐09	II	Cadina‐1,4‐diene (RI, 1534; SI, 97) Cadina‐1(6),4‐diene (RI, 1474; SI, 97) δ‐Cadinene (RI, 1522; SI, 96) β‐Copaene (RI, 1432; SI, 97) (*E*)‐β‐farnesene (RI, 1452; SI, 96 s) Zonarene (RI, 1526; SI, 93)	Epicubenol (RI, 1631; SI, 96) Cadin‐4‐en‐10‐ol (RI, 1659; SI, 94)
*L. nuda*	LnSTS‐01	III	Δ6‐Protoilludene (RI, 1380; SI, 96)	
	LnSTS‐02	III	Δ6‐Protoilludene (RI, 1380; SI, 97)	
	LnSTS‐04	III	Pleostene (RI, 1310; SI, 99) Isobazzanene (RI, 1441; SI, 94)	
	LnSTS‐09	II	Cadina‐1,4‐diene (RI, 1536; SI, 97) Cadina‐1(6),4‐diene (RI, 1474; SI, 97) δ‐Cadinene (RI, 1522, 97) β‐Copaene (RI, 1433; SI, 98) Zonarene (RI, 1527; SI, 93)	Epicubenol (RI, 1631; SI, 96) Cadin‐4‐en‐10‐ol (RI, 1659; SI, 94)
	LnSTS‐19	IV		(*E*)‐nerolidol (RI, 1563; SI, 96)
	LnSTS‐20	IV	β‐Barbatene (RI, 1453; SI, 97)	
	LnSTS‐25	IV	Unknown compound (RI, 1419; SI, ND)	
	LnSTS‐27	Not assigned	Acora‐3,7(14)‐diene (RI, 1417; SI, 95)	
*P. ostreatus*	PoSTS‐01	I	δ‐Cadinene (RI, 1522; SI, 96) Isobazzanene (RI, 1442; SI, 93) α‐Muurolene (RI, 1503; SI, 95)	α‐Muurolol (RI, 1651; SI, 92) Cadin‐4‐en‐10‐ol (RI, 1659; SI, 95)
	PoSTS‐02	I	δ‐Cadinene (RI, 1522; SI, 97) α‐Muurolene (RI, 1502; SI, 96) Zonarene (RI, 1527; SI, 91)	α‐Muurolol (RI, 1652; SI, 97) Cadin‐4‐en‐10‐ol (RI, 1660; SI, 96)
	PoSTS‐03	I	δ‐Cadinene (RI, 1523; SI, 96) Isobazzanene (RI, 1443; SI, 92) α‐Muurolene (RI, 1502; SI, 96)	α‐Muurolol (RI, 1651; SI, 92) Cadin‐4‐en‐10‐ol (RI, 1659; SI, 93)
	PoSTS‐05	II	Cadina‐1,4‐diene (RI, 1538; SI, 98) Cadina‐1(6),4‐diene (RI, 1477; SI, 97) δ‐Cadinene (RI, 1524; SI, 97) (*E*)‐β‐farnesene (RI, 1454; SI, 94) Zonarene (RI, 1530; SI, 93)	Epicubenol (RI, 1632; SI, 96) Cadin‐4‐en‐10‐ol (RI, 1660; SI, 95)
	PoSTS‐06	III	Pleostene (RI, 1310; SI, 100)	
	PoSTS‐11	III		(*E*)‐nerolidol (1563; SI, 96)
	PoSTS‐16	IV	α‐Cuprenene (RI, 1511; SI, 92)	
*T. versicolor*	TvSTS‐01	I	δ‐Cadinene (RI, 1522; SI, 97)	τ‐Muurolol (RI, 1647; SI, 94) Cadin‐4‐en‐10‐ol (RI, 1659; SI, 96)
	TvSTS‐05	II	Cadina‐1,4‐diene (RI, 1538; SI, 98) Cadina‐1(6),4‐diene (RI, 1476; SI, 98) δ‐Cadinene (RI, 1524; SI, 97) β‐Copaene (RI, 1434; SI, 97) Zonarene (RI, 1530; SI, 94)	Epicubenol (RI, 1634; SI, 96) Cadin‐4‐en‐10‐ol (RI, 1661; SI, 93)
	TvSTS‐06	II	Cadina‐1,4‐diene (RI, 1537; SI, 97) Cadina‐1(6),4‐diene (RI, 1476; SI, 97) δ‐Cadinene (RI, 1524; SI, 97) β‐Copaene (RI, 1434; SI, 94) (*E*)‐β‐farnesene (1453; SI, 94) Zonarene (1529; SI, 93)	Epicubenol (RI, 1634; SI, 96) Cadin‐4‐en‐10‐ol (RI, 1660; SI, 92)
	TvSTS‐07	III	Δ6‐Protoilludene (RI, 1382, 95)	
	TvSTS‐12	III	γ‐Cadinene (RI, 1517; SI, 97)	
	TvSTS‐14	IV	α‐Barbatene (RI, 1422; SI, 95) β‐Barbatene (RI, 1455; SI, 97)	
	TvSTS‐16	IV	Dauca‐4(11),8‐diene (RI, 1533; SI, 95) Isobazzanene (RI, 1442; SI, 93)	

*Note*: The retention indices (RI) and degree of similarity (SI) are indicated in parenthesis. Main products of AvSTS‐01 and LnSTS‐25 are listed but were not identified. The SI was calculated using the following equation in Shimadzu GCMSsolution; the RI and MS spectrum of pleostene, produced by PoSTS‐06, were used as reference data. ND, not determined.

$$\mathrm{SI}=1-\left[\sum |\mathrm{Iu}\left(m/z\right)-\mathrm{It}\left(m/z,|\right)\right]/\left[\sum \left\{\mathrm{Iu}\left(m/z\right)+\mathrm{It}\left(m/z\right)\right\}\right]\times 100$$
Iu(*m*/*z*), relative intensity of unknown sample; It(*m*/*z*), relative intensity of target sample.

STSs found in clades I and II of the phylogenetic tree (Figure 1) generally produced multiple sesquiterpene hydrocarbons and alcohols (Table 2). We have previously reported similar for STSs from *P. placenta* and *P. chrysosporium* (Ichinose et al., 2022; Ichinose & Kitaoka, 2018). The vast majority of products of STSs in clades I and II were cadinane‐type sesquiterpenoids and δ‐cadinene was often obtained as a relatively abundant product. STSs producing cadinane‐type sesquiterpenoids have been discovered in a wide variety of basidiomycetes (Clericuzio et al., 2013; Ichinose et al., 2022; Ichinose & Kitaoka, 2018; Liu et al., 2007; Yu et al., 2022). Thus, combining these data and earlier literature, we suggest that the majority of basidiomycetes possess sophisticated metabolic systems to produce cadinene‐type sesquiterpenoids. In addition to biological interest, using these STSs for the rational synthesis of valuable compounds has attracted great interest; for instance, δ‐cadinene, epicubenol and their derivatives show unique bioactivities (Ding et al., 2021; Liu et al., 2007; Takao et al., 2012).

Cadinane‐type sesquiterpenoids were also observed for STSs found in clades III and IV. AbSTS‐07 and TvSTS‐12 produced δ‐cadinene and γ‐cadinene, respectively. Nevertheless, the product profiles of AbSTS‐07 and TvSTS‐12 were relatively narrow compared with those of STSs found in clades I and II of the tree. Other STSs found in clade IV (though not all of them) also showed catalytic specificity. For example, PoSTS‐16 abundantly produced α‐cuprenene with small amounts of minor products. Valuable compounds, such as the antibacterial sesquiterpenoid lagopodin, could be derived from α‐cuprenene (Asai et al., 2020), highlighting the potential advantage of PoSTS‐16 for pharmaceutical and industrial applications. Although an STS (Cop6) from a basidiomycetous fungus *Coprinus cinerea* (*cinereus*) has been demonstrated to produce α‐cuprenene (Agger et al., 2009), in this study, α‐cuprenene synthase (PoSTS‐16) was identified from an edible mushroom for the first time. Interestingly, noncyclic (*E*)‐nerolidol, monocyclic (*Z*)‐α‐bisabolene and tricyclic α‐ and β‐barbatene were also observed for STSs found in clade IV. Thus, one can suggest that STSs found in this clade are functionally diversified to synthesize several types of sesquiterpene skeleton. LnSTS‐27 showed phylogenetic relationships with members of clades IV and V but appeared as an orphan STS in the phylogenetic tree (Figure 1). LnSTS‐27 was functionally characterized to produce a spiro compound, acora‐3(7),14‐diene. Literature that describes the enzymatic synthesis of acora‐3(7),14‐diene is limited. Thus, the activity of LnSTS‐27 to generate this compound is of great interest.

The STSs found in clade III of the phylogenetic tree each generated a single major product. For instance, AvSTS‐03, LnSTS‐01, LnSTS‐02 and TvSTS‐07 were shown to synthesize Δ6‐protoilludene as the major product (Table 2). An STS from *P. placenta* (PpSTS‐08), which was a member of clade III, was also demonstrated to specifically synthesize Δ6‐protoilludene (Ichinose & Kitaoka, 2018). Interestingly, Δ6‐protoilludene synthase was distributed in basidiomycetes across fungal genera and species because this compound is considered to be a key intermediate for the production of bioactive sesquiterpenoids in nature (Bohnert et al., 2014; Cadelis et al., 2020; Engels et al., 2011; Hirota et al., 2003; Nord et al., 2015; Reina et al., 2004; Wang et al., 2006). The sesquiterpene alcohol (*E*)‐nerolidol was observed as the major product of AvSTS‐06, AvSTS‐07, LnSTS‐19 and PoSTS‐11. These STSs would potentially be useful in industry because nerolidol is widely used as a fragrance in commodities such as cosmetics (Chan et al., 2016; Lapczynski et al., 2008). Furthermore, it was clear that AvSTS‐01 produces a sesquiterpene alcohol as a single major product. On the basis of NMR analysis, the sesquiterpene alcohol produced by AvSTS‐01 clearly possesses a hydroxymethyl moiety in the molecule, even though its chemical structure has not been established completely (data not shown). Although further investigations are required, unique reaction mechanisms may be involved for AvSTS‐01 to produce a rare sesquiterpene alcohol. Recently, tremulane STS from the basidiomycete *Irpex lacteus* (IlIS; Il4946) was successfully characterized as producing a novel sesquiterpene alcohol, iltremulanol A (Chen et al., 2022), increasing our understanding of tremulane sesquiterpenoid biosynthesis. Thus, intensive studies on STSs promise to increase the chemical repertoire of backbone structures found among sesquiterpenoids.

**FIGURE 2:**
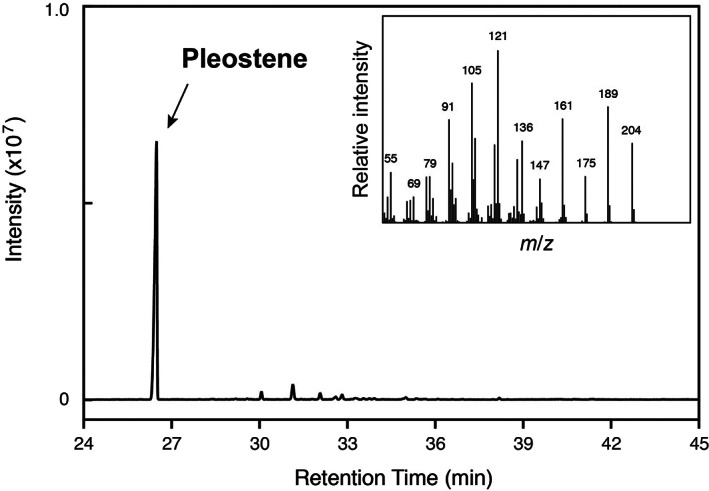
Gas chromatography–mass spectrometry analysis of metabolites produced by PoSTS‐06. Metabolic products were recovered from the culture headspace. Inset: mass spectrum of pleostene.

PoSTS‐06 produced a sesquiterpene hydrocarbon as its single major product (Figure 2). When we performed structural identification based on GC–MS analysis, mass spectra of the product showed poor similarity to known compounds. These results strongly suggested that the product was a novel compound. Therefore, we named the sesquiterpene hydrocarbon ‘pleostene’ and performed further investigations for its structural characterization. A catalytic function to produce pleostene was also demonstrated for LnSTS‐04; however, the enzymatic activity of LnSTS‐04 was slightly lower than that of PoSTS‐06. Both PoSTS‐06 and LnSTS‐04 were found in clade III of the phylogenetic tree (Figure 1).

### Structural characterization of the novel sesquiterpene hydrocarbon pleostene

To increase the production level of pleostene, a heterologous expression system was developed in which PoSTS‐06 was expressed from a pALLF‐based plasmid (detailed in Supporting Information), which was designed to overexpress tHMG1 and ERG20 to increase the FPP level in *S. cerevisiae*. Thereby, pleostene was produced at almost double the level in the normal strain. When recombinant yeast expressing PoSTS‐06 was cultured in SDL medium, pleostene was observed as the single major sesquiterpene product and accumulated in the culture headspace (Figure 2). We successfully recovered pleostene using monolithic silica adsorbents; we obtained 50 mg of purified pleostene. The purified pleostene exhibited an optical rotation value of [α]_D_^20^ = +43.2 (*c* 1.22, chloroform).

**FIGURE 3:**
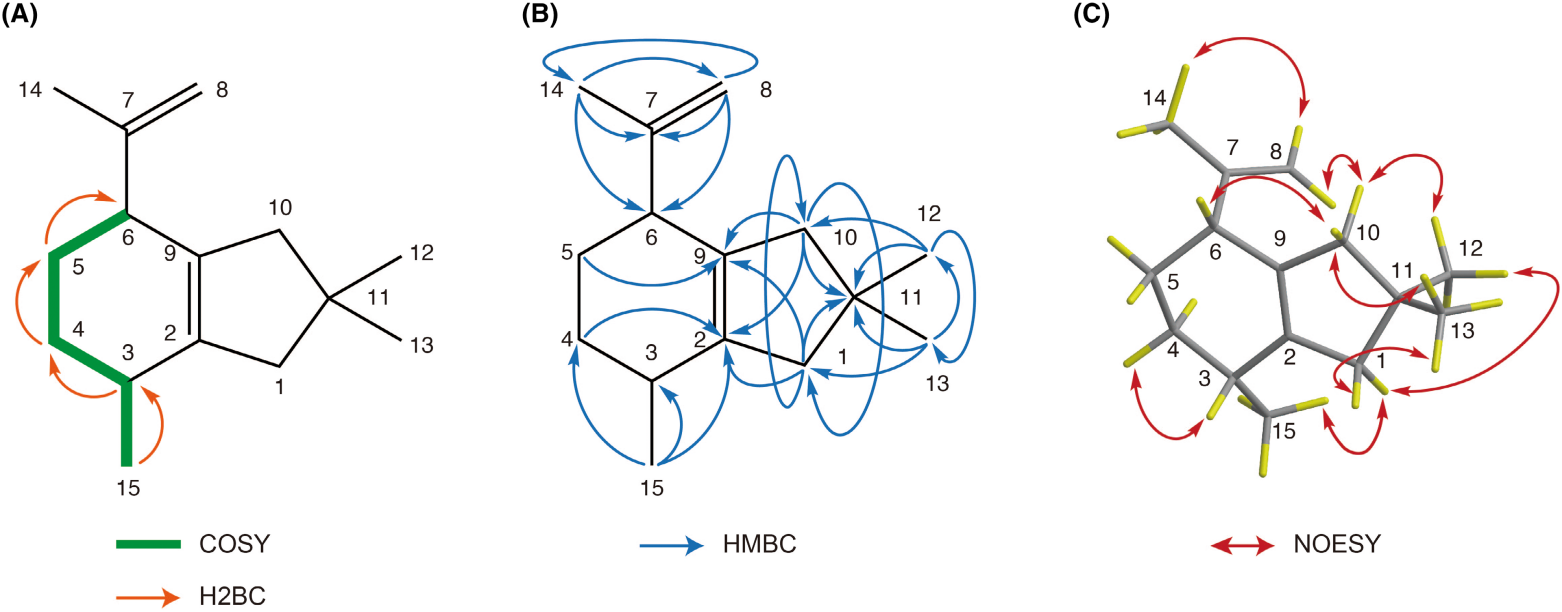
Key correlations observed in (A) ^1^H–^1^H COSY and H2BC, (B) HMBC and (C) NOESY spectra of pleostene.

**FIGURE 4:**
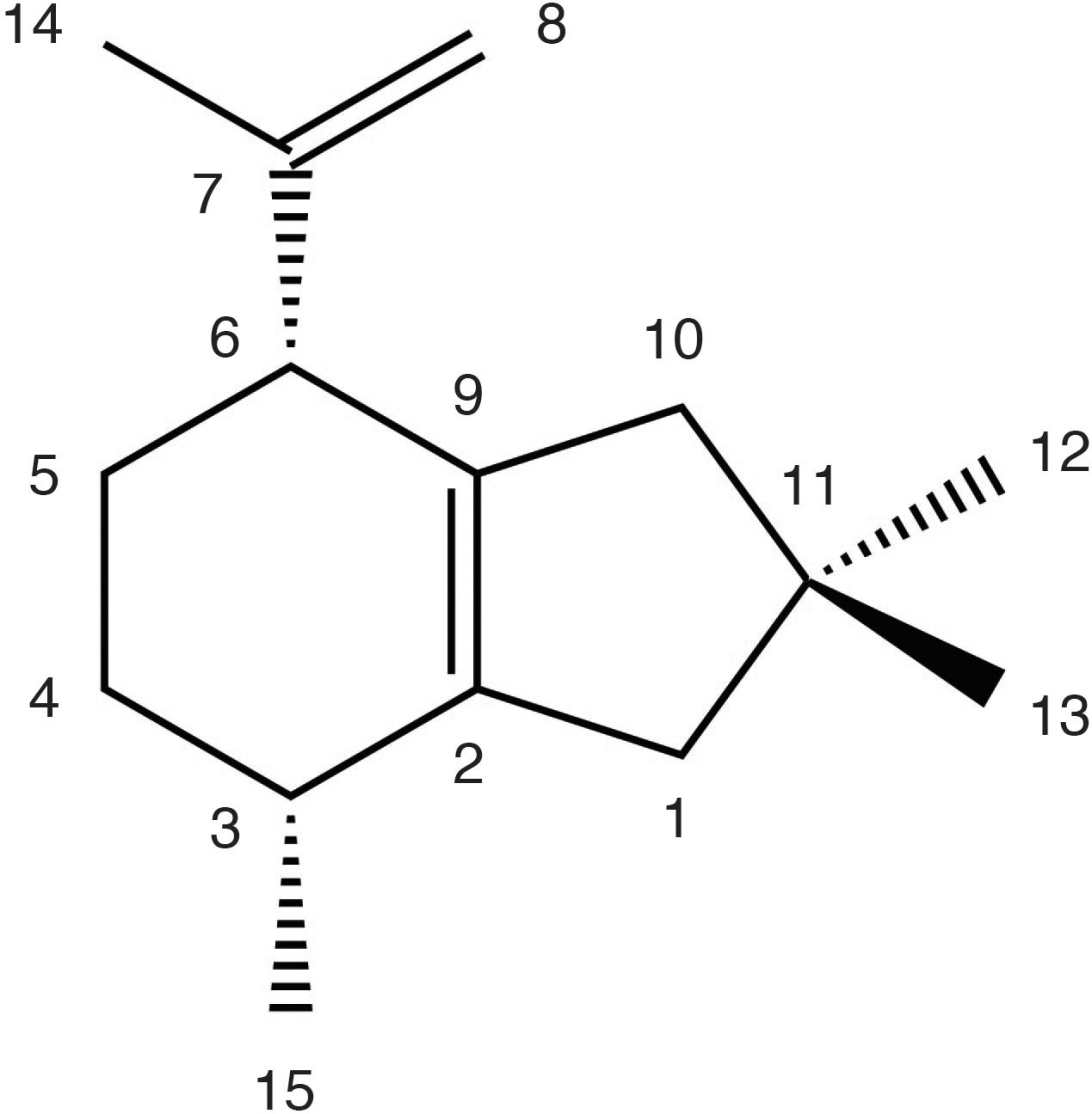
Chemical structure of pleostene.

To confirm the chemical structure of pleostene, we first carried out NMR analyses. ^1^H‐ and ^13^C‐NMR spectral data are listed in Table 3 (see also Figures S5–S12). The ^1^H‐NMR spectrum showed the presence of four methyl groups and one of them was attached to an olefinic carbon atom. The ^13^C‐NMR spectrum showed that pleostene has four olefinic carbons. Combining the data with DEPT analysis, pleostene was revealed to consist of four methyl groups, five secondary carbons, two tertiary carbons and four quaternary carbons, suggesting a bicyclic backbone structure. More detailed analysis revealed that one of the methyl groups (δ_H_ 1.67) and two of the olefinic carbon atoms (δ_C_ 147.7 and 111.2) constituted an isopropenyl moiety. In addition, pleostene clearly involved geminal methyl groups (δ_H_ 30.1 and 30.2) binding to a quaternary carbon atom (δ_C_ 36.4). Assignments were successfully achieved by analysing ^1^H‐ and ^13^C‐NMR, DEPT, COSY, H2BC, HSQC, HMBC and NOESY spectra (Figure 3; see also Supporting Information). Accordingly, the chemical structure of pleostene—an alliacane‐type sesquiterpenoid—was established (Figure  4).

**TABLE 3:** ^1^H‐ and ^13^C‐NMR spectral data for pleostene produced by PoSTS‐06.

Number^a^	Chemical shifts	
	δ_C_	δ_H_ ^b^, mult (*J* in Hz)
1	49.3	α; 2.22, bd (*J*_1α,1β_ = 15.2) β; 1.99, bd (*J*_1β,1α_ = 15.2)
2	139.6	–
3	30.5	2.06, bs
4	28.8	α; 1.29–1.38, m β; 1.57–1.64, m (overlapped)
5	25.8	α; 1.57–1.64, m (overlapped) β; 1.57–1.64, m (overlapped)
6	44.2	2.60, bs
7	147.7	–
8	111.2	a; 4.77, bs b; 4.63, bs
9	133.4	–
10	49.8	α; 2.06, bd (*J*_1α,1β_ = 15.6) β; 1.92, bd (*J*_10β,10α_ = 15.6)
11	36.4	–
12	30.2	1.04, s
13	30.1	1.06, s
14	20.7	1.67, bs
15	19.5	0.96, d (*J*_15,3_ = 7.0)

^a^ Numberings of carbons and protons are shown in Figure 4.

^b^ The following abbreviations are used to describe multiplicity: s, singlet; bs, broad singlet; d, doublet; bd, broad doublet; m, multiplet.

**FIGURE 5:**
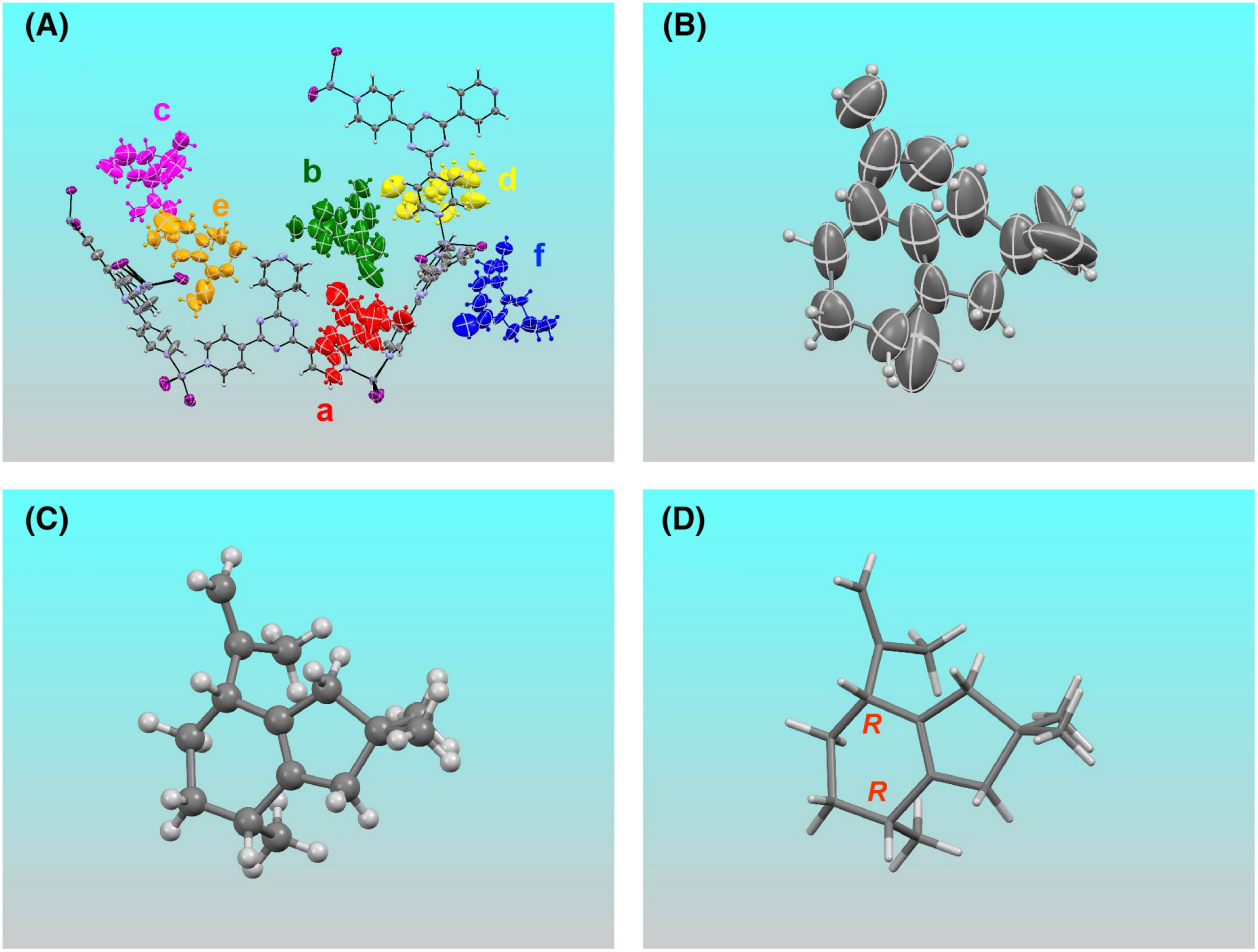
Crystal structure of the complex of pleostene and crystalline sponge. (A) Structure of an asymmetric unit containing six crystallographically independent molecules of pleostene (‘a’ to ‘f’). Panels B to D display only guest ‘a’; (B) ORTEP drawing with 50% probability, (C) ball‐and‐stick representation, (D) another view representing the absolute configuration.

To confirm the absolute configuration of pleostene, X‐ray crystallographic analysis using the crystalline sponge method, which allows direct determination of chemical structures of oily compounds such as pleostene, was performed (Inokuma et al., 2013, 2014; Matsuda et al., 2016; Sakurai et al., 2017; Urban et al., 2016). Using a single crystal of the porous complex [(ZnI_2_)_3_(tpt)_2_(solvent)*x*]_*n*_ as a crystalline sponge (Biradha & Fujita, 2002), pleostene was applied for crystallographic analysis. When the pleostene‐absorbed crystalline sponge was subjected to X‐ray diffraction study, the crystal structure of the host–guest complex (i.e. crystalline sponge–pleostene) was clearly resolved. As shown in Figure 5, six independent molecules of pleostene were observed per asymmetric unit with 75%, 67%, 33%, 33%, 50% and 40% occupancies, respectively; the latter two guests were situated on a two‐fold axis. Notably, all the guest molecules showed the same configuration (Figures S13 and S14). Thus, the absolute configuration of pleostene produced by PoSTS‐06 was confirmed to be 3*R*,6*R* (Figure 5D) based on the Flack parameter of 0.029(4).

Here, the novel sesquiterpene hydrocarbon pleostene was thus identified for the first time. These findings will attract much attention for improving our understanding of the reaction mechanisms of STSs for generating unique sesquiterpene backbone structures. Although a thorough understanding of the chemistry involved in sesquiterpene biosynthesis remains challenging, in this study, a possible reaction mechanism (Figure S15), in which 1,11‐cyclization of FPP followed by complicated skeletal rearrangement might be involved, is proposed; a similar mechanism was suggested for brasilane‐type 5/6 bicyclic skeleton synthesis (Murai et al., 2019; Sato et al., 2020). In addition, the finding of pleostene is of great interest to facilitate advanced applications of alliacane‐type sesquiterpenoids and is of importance to better understand their biosynthesis mechanisms because this compound is likely a key precursor of alliacane‐type sesquiterpenoids in nature. For example, the edible mushroom *Pleurotus cystidiosus* produces alliacane‐type clitocybulols that show cytotoxicity to human prostate cancer cells and/or inhibitory activity against protein tyrosine phosphatase‐1B (Chen & Liu, 2017; Tao et al., 2016; Zheng et al., 2015). In addition, seven alliacane‐type sesquiterpenoids have recently been isolated and identified from the basidiomycete *Clitocybe nebularis* and antifungal effects of nebucane D toward *Rhodotorula glutinis* and cytotoxic activities of nebucane G toward human epidermoid and breast cancer cells were demonstrated (Schrey et al., 2022). Although the latent potential of alliacane‐type sesquiterpenoids (e.g. their pharmaceutical activities) has attracted many researchers, few studies have succeeded in the isolation of naturally occurring alliacane‐type sesquiterpenoids, and their biosynthesis mechanisms are still veiled in mystery. Thus, the present work will open the door for better understanding and use of alliacane‐type sesquiterpenoids.

## CONCLUSIONS

Catalytic functions of STSs from five basidiomycetous fungi—*Ag. bisporus*, *Au. vulgare*, *L. nuda*, *P. ostreatus* and *T. versicolor*—were explored. The functional information obtained here will provide fundamental data that helps us to better understand the metabolic diversity of basidiomycetes responsible for sesquiterpenoid biosynthesis. In addition, the biocatalyst collection of STSs could facilitate industrial applications such as rational synthesis of value‐added chemicals. Furthermore, a novel sesquiterpene hydrocarbon, pleostene, was discovered and identified for the first time, which promises advances in basic science and biotechnology.

## AUTHOR CONTRIBUTIONS

**Natsuki Masunaga:** Investigation (lead); writing – original draft (lead). **Takuya Kitaoka:** Conceptualization (supporting); methodology (supporting). **Hirofumi Ichinose:** Conceptualization (lead); funding acquisition (lead); investigation (supporting); methodology (lead); project administration (lead); supervision (lead); validation (lead); visualization (lead); writing – review and editing (lead).

## FUNDING INFORMATION

This research was supported in part by a Grant‐in‐Aid for Scientific Research B (No. 20H03045 to HI) and for Scientific Research on Innovative Areas (No. 19H04660 to HI) from the Japan Society for the Promotion of Science.

## CONFLICT OF INTEREST

The authors declare that they have no conflicts of interest.

## Supporting information


Data S1

## Data Availability

The data that support the finding of this study are available within the article and its Supporting Information.
