# Corrigendum

**DOI:** 10.1111/1751-7915.14304

**Published:** 2023-07-24

Following the original publication of Masunaga et al. (2023), the author would like to update the optical rotation value on page 640 (left column, line 7) from +43.2 (c 1.22, chloroform) to ‘−66.1 (c 1.75, chloroform)’.

Hence, the sentence should read as follows: The purified pleostene exhibited an optical rotation value of [α]D^20^ = −66.1 (c 1.75, chloroform).

The authors apologize for this error and any confusion it may have caused.
