# Supplementary material for: Biocatalyst collection and heterologous expression of sesquiterpene synthases from basidiomycetous fungi: Discovery of a novel sesquiterpene hydrocarbon
Source: Microb Biotechnol. 2022 Dec 28;16(3):632–44. doi: 10.1111/1751-7915.14204 (PMC9948225; doi:10.1111/1751-7915.14204)
Supplement: Supplementary file 1 — Data S1 [file MBT2-16-632-s001.pdf]

Supporting Information

Biocatalyst collection and heterologous expression of sesquiterpene synthases from basidiomycetous fungi: discovery of a novel sesquiterpene hydrocarbon

Natsuki Masunaga, Takuya Kitaoka, Hirofumi Ichinose*

Faculty of Agriculture, Kyushu University, 744 Motooka, Nishi-ku, Fukuoka 819-0395, Japan

*Corresponding author:

Hirofumi Ichinose, Faculty of Agriculture, Kyushu University, 744 Motooka, Nishi-ku, Fukuoka 819-0395, Japan; Tel & Fax: +81-92-802-4666; Email: ichinose@agr.kyushu-u.ac.jp

Table of Contents

Text

1. Phylogenetic analysis of basidiomycetous STSs
2. RT-PCR conditions for cDNA isolation of STSs
3. PCR conditions for the construction of pGYRG-based expression plasmids
4. Composition of drop out supplement
5. Construction of pALLF-based plasmid and heterologous expression of PoSTS-06
6. Data analysis of NMR spectra of pleostene
7. Crystallographic data and crystal structure analysis

Tables

Table S1. List of possible STS genes

Table S2. Basidiomycetous STSs used for phylogenetic analysis

Table S3. List of primers used for cDNA isolation of STSs

Table S4. List of primers used for construction of pGYRG-based expression plasmids

Table S5. List of primers used for construction of pALLF-based expression plasmids

Figures

Fig. S1. Illustration of construction of the pALLF-based expression plasmid

Fig. S2. Multiple alignment of aspartate-rich and NSE/DTE motifs found in the STSs

Fig. S3. Total ion chromatogram analysis of products by GC-MS

Fig. S4. Mass spectra of products synthesized by STSs

Fig. S5. ^{1}H-NMR spectrum of pleostene synthesized by PoSTS-06

Fig. S6. ^{13}C-NMR spectrum of pleostene synthesized by PoSTS-06

Fig. S7. DEPT ^{13}C-NMR spectra of pleostene synthesized by PoSTS-06

Fig. S8. H–H COSY spectrum of pleostene synthesized by PoSTS-06

Fig. S9. H2BC spectrum of pleostene synthesized by PoSTS-06

Fig. S10. HSQC spectrum of pleostene synthesized by PoSTS-06

Fig. S11. HMBC spectrum of pleostene synthesized by PoSTS-06

Fig. S12. NOESY spectrum of pleostene synthesized by PoSTS-06

Fig. S13. Ball-and-stick diagrams for the crystal structure of guest molecules “a” to “f”

Fig. S14. Illustration representing restraints in pleostene

Fig. S15. Proposed reaction pathway for pleostene synthesis by PoSTS-06

Others

Nucleotide and amino acid sequences of STSs.

References

1. Phylogenetic analysis of basidiomycetous STSs

A phylogenetic tree was constructed using amino acid sequences deduced from isolated cDNAs or predicted exon sequences found in genomic databases (Table S1) and literature data (Agger et al., 2009; Wawrzyn et al., 2012; Ichinose and Kitaoka, 2018; Ichinose et al., 2022). Multiple sequence alignment was carried out using the ClustalX program with a gap penalty of 10 and a gap extension penalty of 0.2 (Thompson et al., 1994). The phylogenetic tree was constructed using the Unweighted Pair Group Method with Arithmetic Mean (UPGMA), with the Jones–Taylor–Thornton matrix using PHYLIP v3.695 (Felsenstein 1989), and visualized using the FigTree program (http://tree.bio.ed.ac.uk/software/).

2. RT-PCR conditions for cDNA isolation of STSs

Total RNA was extracted individually from 5-, 7-, 10-, and 12-day old mycelia using the acid guanidium–phenol–chloroform method and further purified using RNeasy Plant Mini Kit (QIAGEN, Hilden, Germany). The concentration of RNA was calculated from the absorbance at 260 nm. Equal quantities of RNA isolated from mycelia of different ages were then mixed and used for RT-PCR (Ichinose and Kitaoka, 2018; Ichinose et al., 2022). First-strand cDNAs were synthesized with ReverTra Ace® reverse transcriptase (TOYOBO, Osaka, Japan) in the presence of the oligo(dT) primer (5′-TTTTTTTTTTTTTTTTTTV-3′; V = A, C or G). The reverse transcription reaction was carried out in accordance with the manufacturer’s protocols. The following amplification of the target gene was performed by nested PCR using Phusion High-Fidelity DNA Polymerase (New England Biolabs Japan, Tokyo, Japan) and/or KOD FX Neo DNA Polymerase (TOYOBO) following the manufacturer’s protocols. For first-round

PCR, the reaction mixture (25 μL) contained 0.25 μL of the reaction mixture from reverse transcription, dNTP (200 or 400 μM, depending on the enzyme used), primers (1 μM each), dimethylsulfoxide (DMSO; 2%), and DNA polymerase in the appropriate reaction buffer. The PCR reaction for the first round included: denaturation at 96 °C for 2 min; 20 cycles of 96 °C for 10 s, 60 °C for 30 s, and 68 °C for 1 min; and final extension at 68 °C for 1 min. After the first-round PCR, 1 μL of the reaction mixture was used as the template for second-round PCR. The reaction mixture and thermal conditions were the same as those for the first-round PCR, but included 45 cycles for amplification. The nucleotide sequences of the primers used are listed in Table S3.

3. PCR conditions for the construction of pGYRG-based expression plasmids

PCR amplification was carried out using Phusion DNA polymerase. The reaction mixture (50 μL) contained pBluescript II KS(–) harboring STSs (1 ng), dNTP (200 μM), primers (1 μM each), DMSO (2%), and Phusion DNA polymerase (0.02 U/mL) in Phusion HF buffer. The reaction conditions were as follows: denaturation at 96 °C for 2 min; and 35 cycles of 96 °C for 10 s, 60 °C for 30 s, and 68 °C for 1 min. The nucleotide sequences of the primers used in this section are listed in Table S4.

4. Composition of dropout supplement

Complete supplement mixture consisted of adenine (10 mg), L-arginine (50 mg), L-aspartic acid (80 mg), L-histidine hydrochloride (20 mg), L-isoleucine (50 mg), L-leucine (100 mg), L-lysine hydrochloride (50 mg), L-methionine (20 mg), L-phenylalanine (50 mg), L-

threonine (100 mg), L-tryptophan (50 mg), L-tyrosine (50 mg), uracil (20 mg), and valine (140 mg). Dropout supplement was prepared by making supplement mixture that lacked the appropriate amino acid or nucleotide from the complete supplement mixture.

5. Construction of pALLF-based plasmid and heterologous expression of PoSTS-06

The experimental strategies in this section are outlined in Figure S1. Nucleotide sequences of the primers used are listed in Table S5. Briefly, promoter, terminator and coding sequence regions of TDH3, TPI1, ERG20, and tHMG1 were amplified from genomic DNA of *S. cerevisiae* (AH22) using Phusion High-Fidelity DNA Polymerase and seamless cloning was carried out using the In-Fusion HD cloning kit (TaKaRa Bio USA, Mountain View, CA, USA) following the manufacturer's protocols. In step 1, a gene fragment consisting of the TDH3 promoter (TDH3p), coding sequence, and TDH3 terminator (TDH3t) was amplified using the primer combination tdh3pF and tdh3tR and cloned into the *Sma*I/*Eco*RV sites of pBluescript II KS(−). In step 2, the plasmid obtained from step 1 was linearized by inverse PCR using the primer combination tdh3_invF and tdh3_invR. In steps 3 and 4, a gene fragment encoding tHMG1 was amplified using the primer combination thmg1_recF and thmg1_recR and ligated into the linearized plasmid obtained in step 2 by seamless cloning. In step 5, an expression cassette for tHMG1 (TDH3p–tHMG1–TDH3t) was amplified from the plasmid obtained in step 4 using the primer combination tdh3t_infF_Y2 and tdh3p_infR_Y2. In step 6, plasmid pYES2 (Thermo Fisher Scientific, Waltham, MA, USA) was linearized by inverse PCR using the primer combination f1ori_invF and f1ori_invR. In step 7, the PCR fragment obtained from step 5 and linearized plasmid obtained from step 6 were ligated by seamless cloning. In step 8, the

circular pBluescript II KS(−) was linearized by inverse PCR using the primer combination pBlue_invF and pBlue_invR. In step 9, the promoter region of TPI1 (TPI1p) and the coding sequence of ERG20 were amplified using the primer combinations tpi1p_inf_F and tpi1_infR, and erg20F and erg20_infR, respectively. In step 10, the PCR fragments obtained from step 9 and linearized pBluescript obtained from step 8 were ligated by seamless cloning. In step 11, the gene fragment consisting of TPI1p and ERG20 was amplified using the primer combination tpi1p_infF_Y2 and erg20_infR_Y2. In steps 12 and 13, circular plasmid obtained from step 7 was digested with *Spe*I/*Xho*I and then ligated with the PCR fragment from step 11. The resultant plasmid was designated pALLF. In step 14, the expression cassette for PoSTS-06 was amplified from the pGYRG-based expression plasmid using the primer combination gapdh_NaeI_F and gapdh_NaeI_R. Finally, in steps 15 and 16, pALLF was linearized by digestion with *Nae*I and then ligated with the expression cassette for PoSTS-06 amplified by PCR (obtained from step 14), resulting in construction of a pALLF-based expression plasmid for PoSTS-06. The expression plasmid was transformed into *S. cerevisiae* (InvSC1). A transformant was isolated by auxotrophic selection on synthetic dextrose agar plates containing 2% glucose, 0.67% yeast nitrogen base without amino acids, 20 mg L^{-1} L-histidine, 50 mg L^{-1} L-tryptophan, 100 mg L^{-1} leucine, and 1.5% agar. A single transformant was used for production of sesquiterpene hydrocarbon (see section 2.5 in the main manuscript).

6. Data analysis of NMR spectra of pleostene

Interpretation of ^{1}H–^{1}H COSY and H2BC spectra revealed the spin system H-15/H-3/H-4/H-5/H6. Although correlations between H-3/H-4 and H-4/H-5 were weak and/or overly

complicated in the COSY spectrum, the H2BC spectrum consistently agreed with this plausible spin system. Diagnostic long-range couplings of H-8/H-14 and H1/H6 were also recorded in the COSY spectrum (Barfield and Chakrabarti, 1969; Dexter and Silverson, 1968). Unexpectedly, however, the H2BC spectrum was observed with improper cross-peaks between H-12/C-13 and H-13/C-12, albeit weak. In addition, clear HMBC from the methyl groups and terminal vinyl protons was observed as follows: H_3-12 (δ 1.06) to C-1/C-10/C-11/C-13; H_3-13 (δ 1.04) to C-1/C-10/C-11/C-12; H_3-14 (δ 1.67) to C-6/C-7/C-8; H_3-15 (δ 0.96) to C-2/C-3/C-4; and H-8 (δ a, 4.77; b, 4.63) to C-6/C-7/C-14. These data combined with 1D-NMR and COSY spectra were indicative of the connections C-3 to C-2/C-4/C-15, C-4 to C-5, C-5 to C-6, C7 to C-6/C-8/C-14, and C-11 to C-1/C-10/C-12/C-13. Furthermore, diagnostic HMBC signals of H-4 (δ α, 1.29–1.38; β, 1.54–1.64) to C-2/C-3/C-5/C-6/C-15 were supportive of the connection of C-3 to C-2, and H-5 (δ α, 1.54–1.64; β, 1.54–1.64) to C-3/C-4/C-6/C-7/C-9 was conclusive of the connection of C-6 to C-9. Additionally, by combining with data from 1D-NMR and COSY spectra, HMBC signals of δ 2.22 and/or 1.99 (*finally assigned H-1α, β*) to C-2/C-9/C-10/C-11/C-12/C-13 and δ 2.06 and/or 1.92 (*finally assigned H-10α, β*) to C-1/C-2/C-9/C-11/C-12/C-13 were indicative of the presence of a cyclopentene moiety consisting of geminal methyl groups at C-11 and a C-2/C-9 double bond. Nevertheless, HMBC analysis only allowed interchangeable assignments for H-1/C-1 and H-10/C-10 that arose because of weak/overlapped signals representing their correlation between H-3/C-3 and/or H-6/C-6. Next, we analyzed NOESY data to achieve complete assignment and reveal the relative stereochemistry of pleostene. As shown in Figure S12, NOESY correlations between H-6 (δ 2.60) and δ 1.92 (*finally assigned H-10β*), H-8b (δ 4.63) and δ 2.06 (*finally assigned H-10α*),

and H_3-15 (δ 0.96) and δ 2.22 (*finally assigned H-1α*) made it reasonable to assign H-10 and H-1 signals (Table 3). Furthermore, NOESY correlations of H-8b/H-10α, H-10α/H-12, H−12/H-1α, and H-1α/H-15 enabled assignment of the relative configuration of pleostene as 3*R**,6*R** whose absolute configuration was finally confirmed by X-ray structural analysis (see section 7).

7. Crystallographic data and crystal structure analysis

Structural analysis using the crystalline sponge method was conducted by Mitsui Chemical Analysis & Consulting Service, Inc. (Chiba, Japan) as described previously (Inokuma et al., 2013; Inokuma et al., 2014). A single crystal of the porous $[(ZnI_2)_3(tpt)_2(solvent)_x]_n$ complex (tpt, tris(4-pyridyl)-1,3,5-triazine) was treated with 10 μl of *n*-hexane solution of pleostene (100 μg/μl) and incubated for 3 days at 50 °C to allow uptake of pleostene into the pores of the crystalline sponge. The prepared single crystal of the crystalline sponge/pleostene complex was applied for X-ray diffraction data collection. Single crystal X-ray diffractometry was performed using D8 VENTURE apparatus equipped with a CuKα X-ray source and Photon II detector (Bruker AXS, Madison, WI, USA). The sample crystal was cooled to 93 K using a cold nitrogen stream. For crystal structure analysis, crystallographic data were refined after determining the configurations of all stereogenic centers using SHELXL ver. 2017/1. The scaffold of pleostene was refined with restraints on the atomic displacement parameters (SIMU), using the default standard uncertainty strength. Six independent guest molecules were characterized as geometrically equal molecules by the restraint (SAME, FLAT; see also Fig. S14). Crystallographic data is as follows: refined formula, $C_{116.70}H_{119.52}I_{12}N_{24}Zn_6$; formula weight,

3773.30; monoclinic *C*2, a = 35.8999(9) Å, b = 14.8982(3) Å, c = 31.0604(7) Å, α = 90°, β = 102.3610(10)°, γ = 90°, V = 16227.4(6) Å^3, Z = 4, R_{int} = 0.0533, R_1 = 0.0480, wR_2 = 0.1440, Flack parameter (Parsons') = 0.029(4).

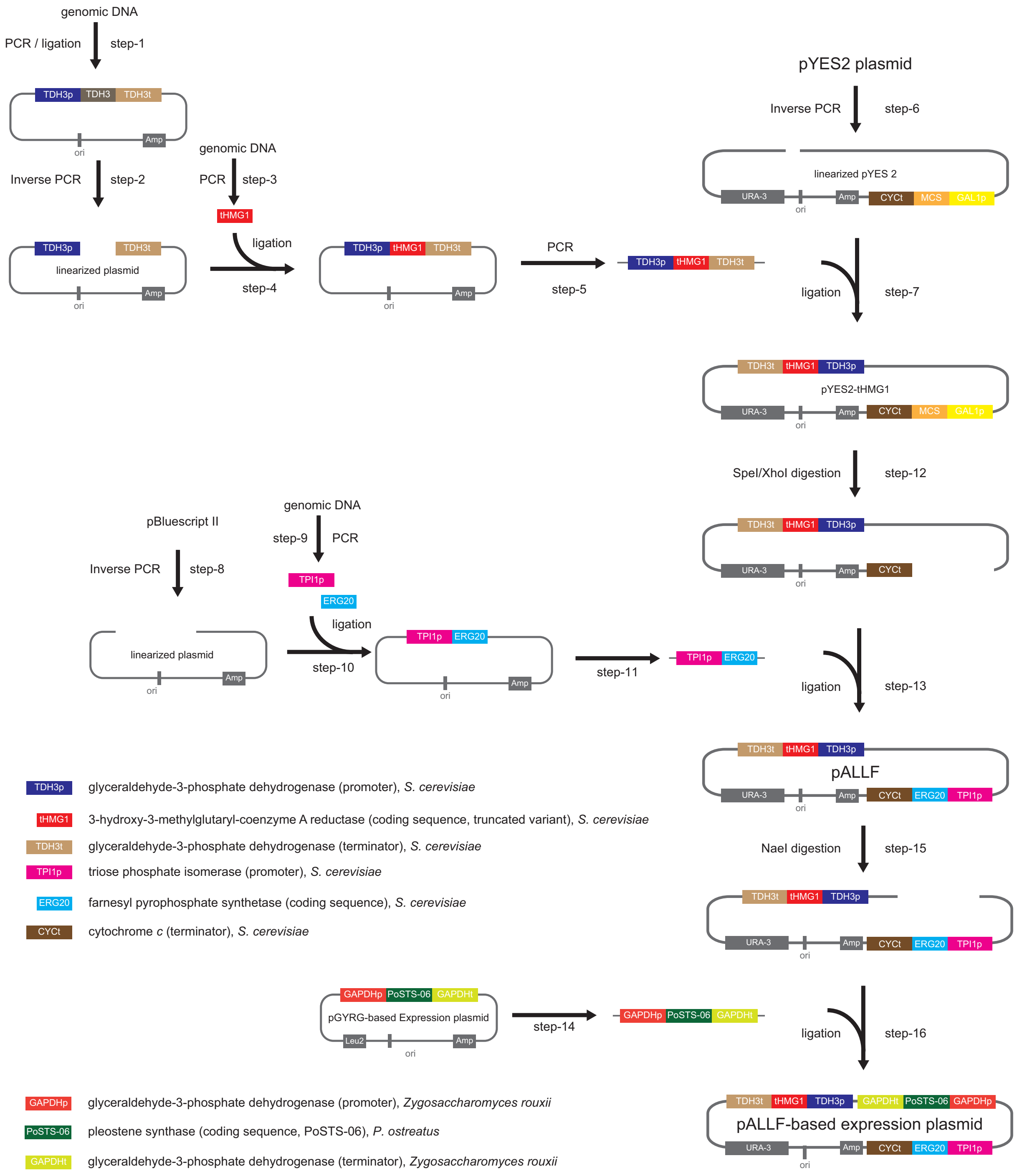

Fig. S1 Illustration of construction of the pALLF-based expression plasmid

```
             DDxxD motif                       NSE/DTE motif
                                **--*                     **--*---*
AvSTS_09     YAQFRTICDFVNLLFVVDEVSDDQSGAD      AAADMVCWANDVYSYNMEQAKGHTGNNIVTVLMKAK
TvSTS_06     YEQFRTCCDFVNLLFVVDEVSDDQNGKD      AAADMVCWSNDVYSYNMEQAKGHTGNNIVTVLMRQK
PoSTS_05     YEQFRTCCDLVNLLFVIDEVSDDQSGKD      AAVDMVSWSNDVYSYNMEQAKGHSGNNIVTVLMKSK
LnSTS_09     YDVFKICCDFVNVLFVFDELSDEQDGKD      AAVDLVCWANDVYSYDMEQSKGISGNNIVTVLMHDK
AbSTS_05     YEEFRTTCDFVNVLFVVDEISDEQSGKD      AACDHVCWCNDVYSYNVEQSKGHTGNNVVTVLMNDR
AvSTS_11     AEEYRMCCDFINLLFTIDEISDEQDHAG      ASVDMVCWANDLYSYNMELNQGLEGNNFLTVLIQKQ
TvSTS_05     YQQFRTTCDFVNLLFTVDEISDDQNGKG      STVDMVCWSNDLYSYNMEQAMGHTGNNVITVLMQHK
LnSTS_10     CPQLRVCNDFLTYLFHLDNLSDDMDNHG      AANDLVTWSNDIFSYNVEQSKGDTHNMIPVVMNEEG
PoSTS_01     APQLRVCCDFMNYLFHLDNLSDDMDNRG      AANDLVTWSNDIFSYNVEQSKGDTHNMIPVVMHEEG
AbSTS_01     CPQLRVCNDFLTFLFHLDNLSDDMDNRG      AANDLVTWSNDIFSYNVEQSKGDTHNMIPVVMYQKD
TvSTS_01     YDQFRVCCDWINYLFHLDNICDEMDDRT      AANDLVTWSNDIFSYNVEQSNGDTHNMIVVVQTQEQ
PoSTS_02     AFHLRVCVDFMNYLFNLDDWLDEFDVEG      ATNDLVTWSNDLFSYNVEQSRGDTHNMIPVIMHQRD
AvSTS_12     SERLRVVSDFMNFLFHLDNISDGMMARD      GANDLVTWSNDIFSYNVEQARGDTHNMICIFMALEG
AvSTS_13     SERLRVVSDFMNFLFHLDNISDGMMAKD      GANDLVTWSNDIFSYNVEQARGDTHNMICIFMALEG
LnSTS_08     PERLRVVSDFMNYLFHLDNISDGMMTRE      GTNDLVTWSNDIFSYNVEQSRGDTHNMIVILMKYHG
PoSTS_03     PDRLRVVSDFMNYLFHLDNISDGMMTRE      GTNDLVTWSNDIFSYNVEQARGDTHNMIVILMKYHG
TvSTS_04     FDELRVCTDFMNFLFTLDDWTDEFDVTG      ATNDLVSWANDVLSYNAEQARGDTHNLVCVLMHMNG
AvSTS_06     RQHLRTGCDLMMLFFVFDEFTDKEDGKG      LVAESVVVTNDTYSYNIEQAAGHQGHNIVTVVMREK
AvSTS_07     KERARTGCELMILFFIFDEFTDQEDGPG      LVAESIVLTNDTYSYNIEQAAGHDGHNIVTVAMREL
AvSTS_01     KARLRTGCDLMMLFFVFDEYTDLSHGND      LVTDVILLDNDLCSYNKEQANGEELHNILTIVMAEL
AvSTS_04     KARLRTGCDLMMLFFVFDEYTDLSHGND      LVTDVILLDNDLCSYNREQANGEELHNILTIVMAEL
LnSTS_01     KEGCRVGCDLMNLFFVIDEYSDVADQCG      TSIDMLIIGNDLCSYNVEQARGDDGHNLVTIVMNER
LnSTS_02     RDGCRIGCDLMNLFFVIDEHSDVADMKT      LCIDMLIIGNDLCSYNVEQARGDDGHNLVAIVCHEM
AvSTS_03     KAGARIGCDLMNMFFVYDEYSDVAPADE      LSIDMILLGNDIASYNLEQARGDDNHNIVTIVMHQE
LnSTS_03     RAGCRVVCDLMYLFGVFDEHSDVMDARS      CTVDMLCLANDMYSFNVEQAKGDD-HNIITLIMLHE
TvSTS_07     KDHLRAACDLMNVFFVFDEQTDIADTTR      CITDMLIFDNDLLSYRKEYAAGDDMHNIITLVMNEK
TvSTS_09     RKALRTSMDLINILFVVDEYTDVESAPA      CIVDMITTVNDIISYNREQATHIDDCNLLTVVMREL
TvSTS_10     RETLRTSIDLMDLFFVIDEYTDHELAPA      CIIDMITTINDILSYNREQATHTDDYNLVTVVMREL
LnSTS_04     GAHFRVACDLMNLFFVFDEYSDRADGEA      AAVDMTILANDVYSYNKEQAKGEDSHNLVAVVMKEH
PoSTS_06     GTHYRAACDLMNLFFVFDEFSDAENGDV      GAVDMTILANDVYSWNVEQCRGEDRHNLVAVAMREK
LnSTS_07     RAHFRTACDLMNILFAVDDISDRLNPTE      IAIELIAVANDIVSFNVEQARGDIHNLVIVLMDGDK
AbSTS_07     FELRLYIAYYTGLLLCVDDNFDIQSDGI      ELEDIINYVNDITSFYKEELAGEDENTVSLLAKLNN
AbSTS_09     FELKLYVAYYSGILLCVDDNYDTQSRVD      ELEDIINYVNDIASFYKEELAGEDENAVSLLARLND
LnSTS_18     MSIRVSIALYTACGIYLDDKFKTDVDAV      ELMIFINNGNDILSFFKEEADGESVNRISLLAACRG
PoSTS_16     LPTRVIIGIYTAFMIYLDDISSSDIEAV      EINVYVENTNDVLSFYKEQMAGDDINYASLLSHSYS
LnSTS_27     DALRTYIIFYIMFMFYVDDKYFEQSDKE      EICDYLCYVNDIMSFYKEELVGETENLVSLLAAVTD
LnSTS_20     LTEKTNIALYNWYLIYIDDMSSKDTGPF      DMNFWISVTNDILSFHKEELAGERANYVYNRAYIED
LnSTS_21     FEEKKSIALYIWYMFYVDDMASRDVDSF      DMIFWISLSNDLLSFHKEELAGDTNNYVHIRALMEE
LnSTS_25     LSEKTVIAIFNWFIIYIDDTSPGDVSPF      EMNFWISGVNDLMSFPKEQLAGERNNYVHVRARTEE
LnSTS_19     FEEQKVIALVNWFVIYLDDVDSEPCAAF      DMDFWICAANDLLSYHKEMLAGETNNYVSSRALVEK
TvSTS_14     YEHQRIIALYTTYLTYVDDLGGRDLDAL      EIEFYTDTINDILSFYKESLAGETDNLIHLRAAAEQ
TvSTS_16     LEHRFFVARYCAYFLYADDLPGRCLDAL      EIEYWIGATNDLLSFYKEELAQETNNYIHIRAAMEQ
TvSTS_12     AAHLETCIAFFFWAFSFDDLSDEGALQS      AVIDIMTWPNDLCSFNKEQA-DGDFQNLVFCIMIER
PoSTS_11     LGQLRTCLDYLTLAFIFEEITENTSSSQ      AAVEMVFLANDIYSYKKEKMAGATQNNVITVIIEDP
TvSTS_13     ASQLFAITKFLIVIMHWVDRPRSLYADE      DAADIIAWSTDVAAYARRPHSSNIIAVLMHERRYTA
PoSTS_09     APQLKDLADIMSAFICIQVRLVGACDMA      SALKIIVGFLQLVSYNLHAS-KKSSGNAVAVVMSLR
```

Fig. S2 Multiple alignment of aspartate-rich and NSE/DTE motifs found in the STSs

The aspartate-rich and NSE/DTE motifs are highlighted in yellow and magenta, respectively. The glutamic acid residues corresponding to the aspartate-rich region in PoSTS-11 are highlighted in cyan.

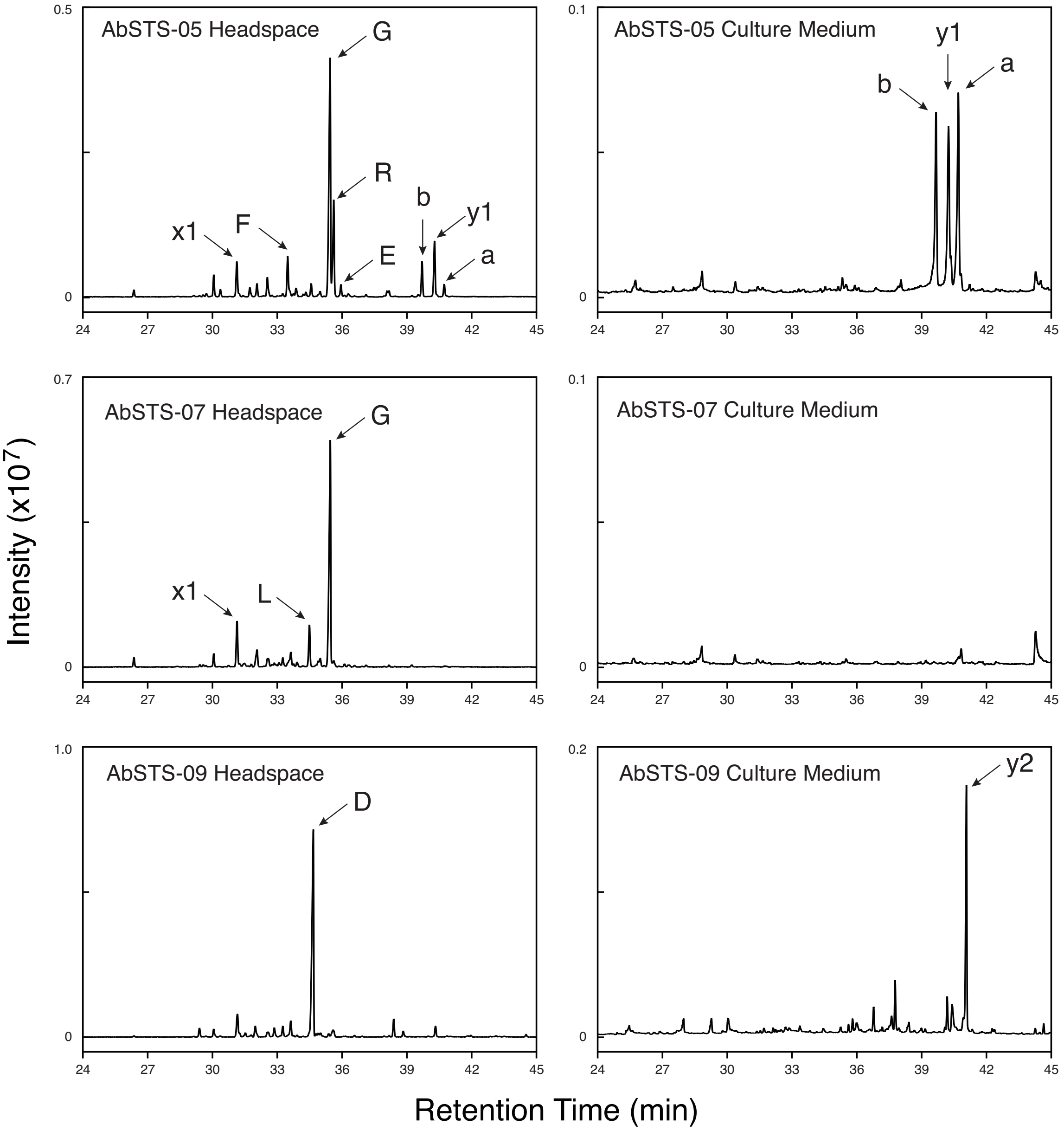

Fig. S3 Total ion chromatogram analysis of products by GC-MS

Sesquiterpene hydrocarbons: A; acora-3(7),14-diene, B; α-barbatene, C; β-barbatene, D; (*Z*)-α-bisabolene, E; cadina-1,4-diene, F; cadina-1(6),4-diene, G; δ-cadinene, H; γ-cadinene, I; β-copaene, J; α-cuprenene, K; dauca-4(11),8-diene L; epizonarene, M; farnesene, N; isobazzanene, O; α-muurolene, P; pleostene, Q; Δ6-protoilludene, R; zonarene. ***Sesquiterpene alcohols:*** a; cadin-4-en-10-ol, b; epicubenol, c; α-muurolol, d; τ-muurolol, e; (*E*)-nerolidol. Unidentified sesquiterpene hydrocarbon (*m*/*z*, 204) and sesquiterpene alcohol (*m*/z, 222) are represented with x1 to x5 and y1 to y3, respectively.

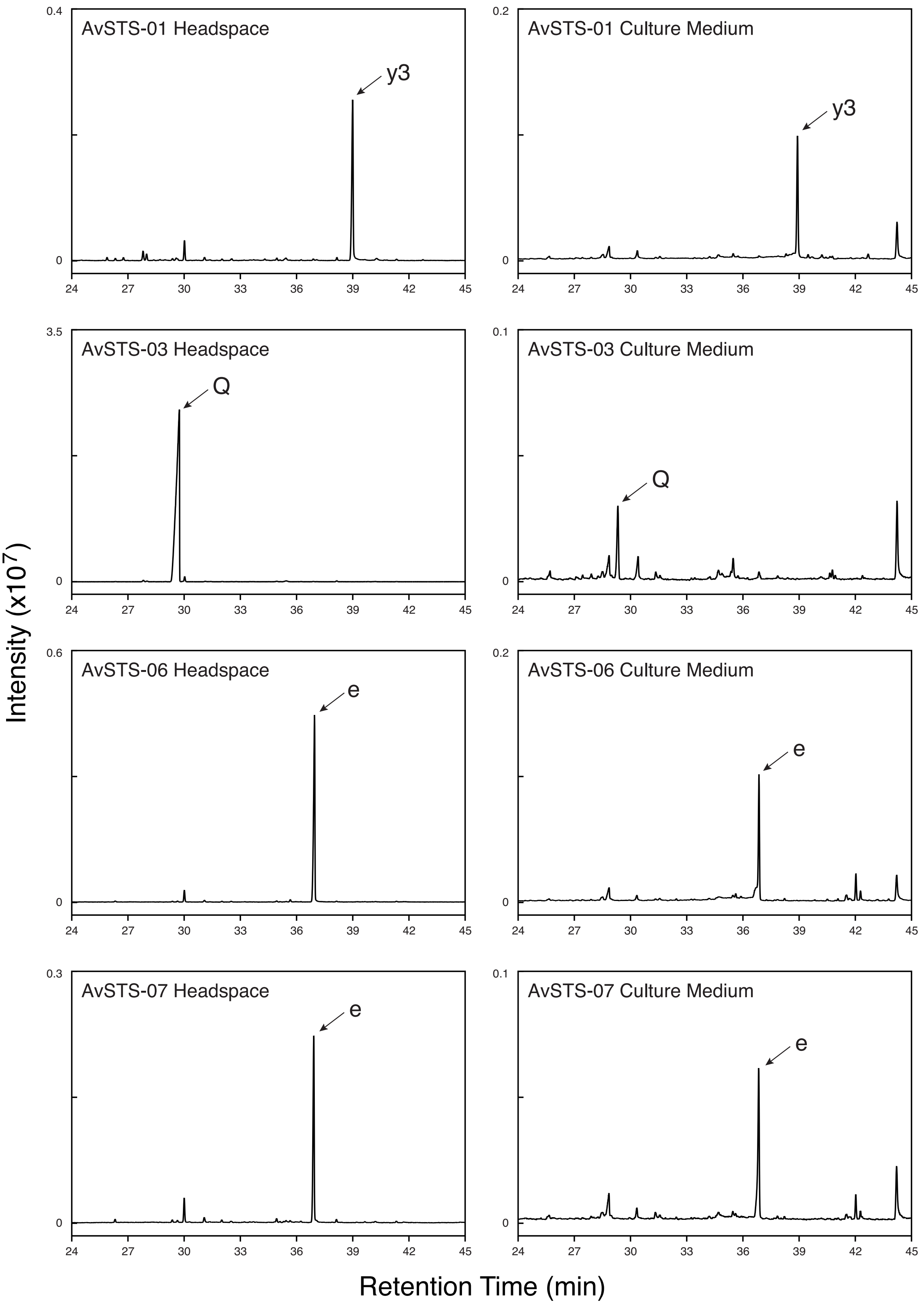

Fig. S3 Total ion chromatogram analysis of products by GC-MS (*continued*)

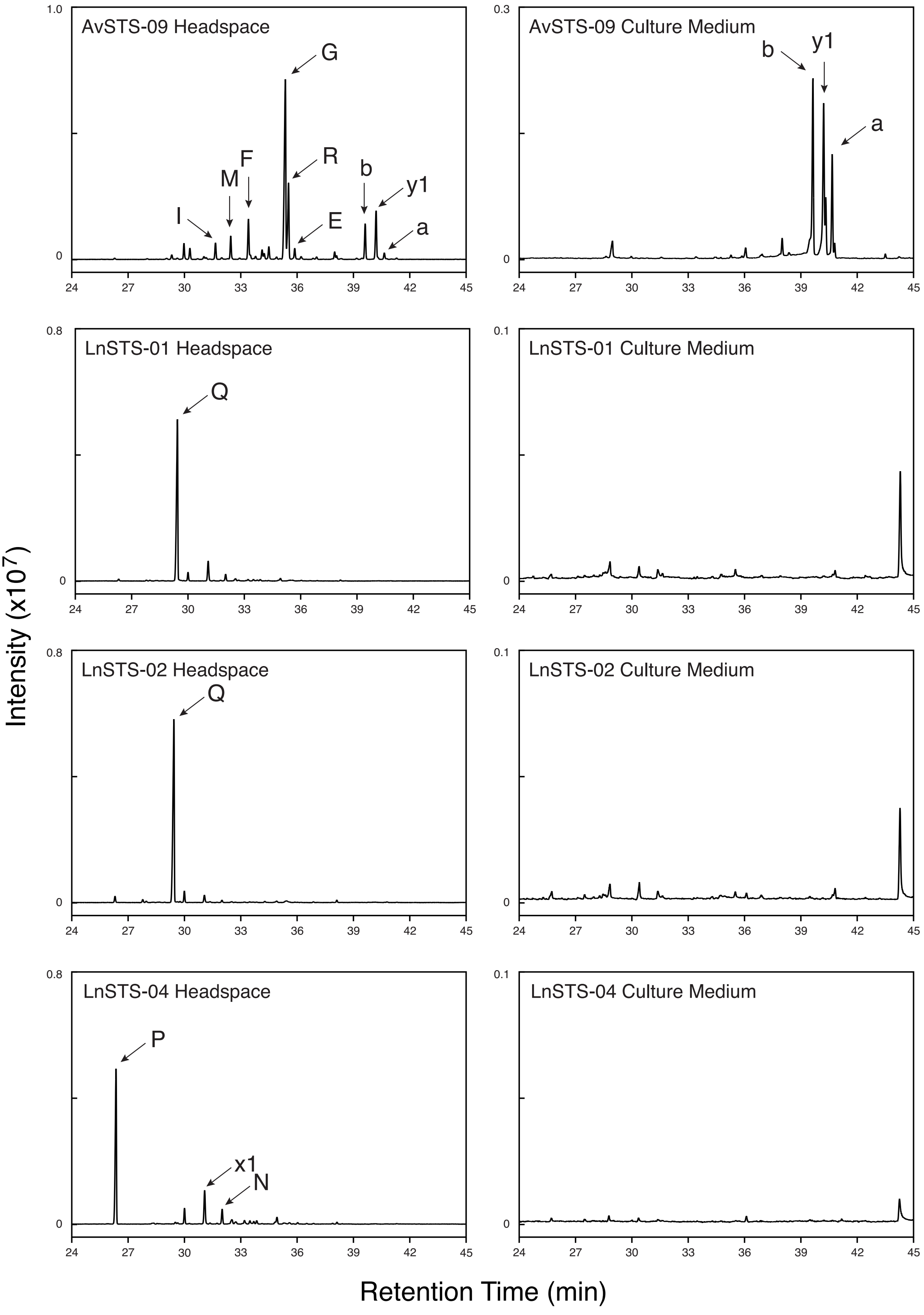

Fig. S3 Total ion chromatogram analysis of products by GC-MS (*continued*)

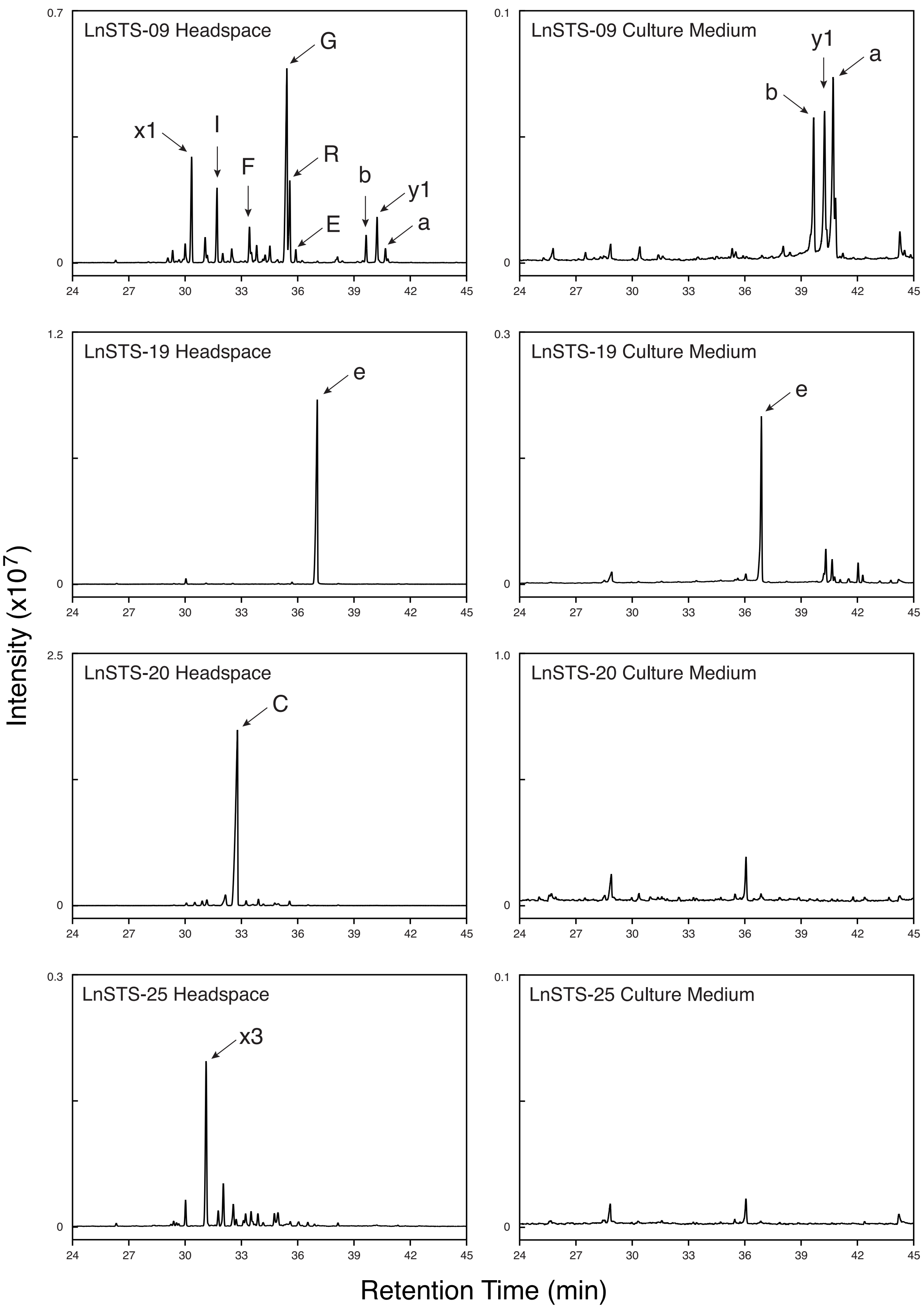

Fig. S3 Total ion chromatogram analysis of products by GC-MS (*continued*)

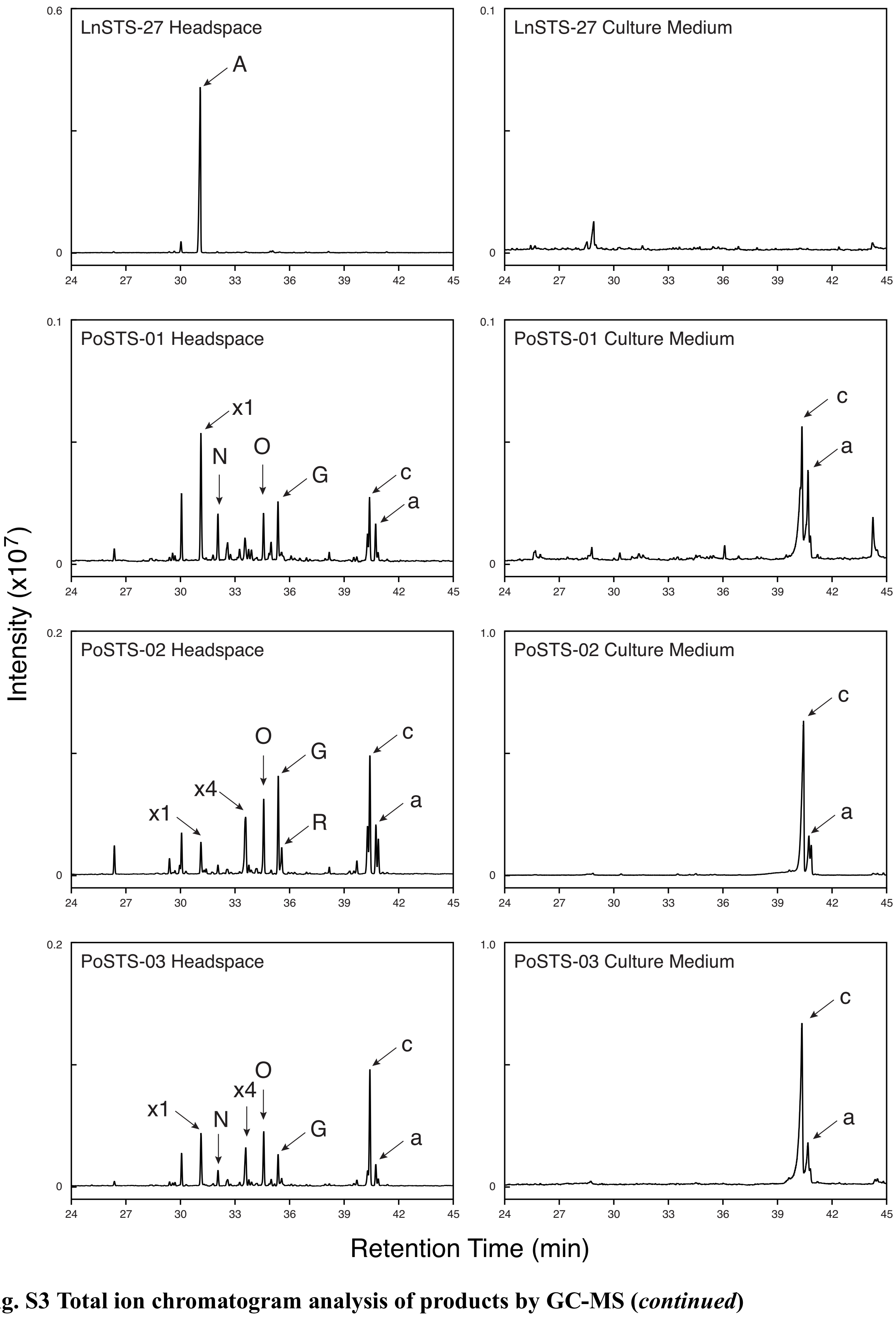

Fig. S3 Total ion chromatogram analysis of products by GC-MS (*continued*)

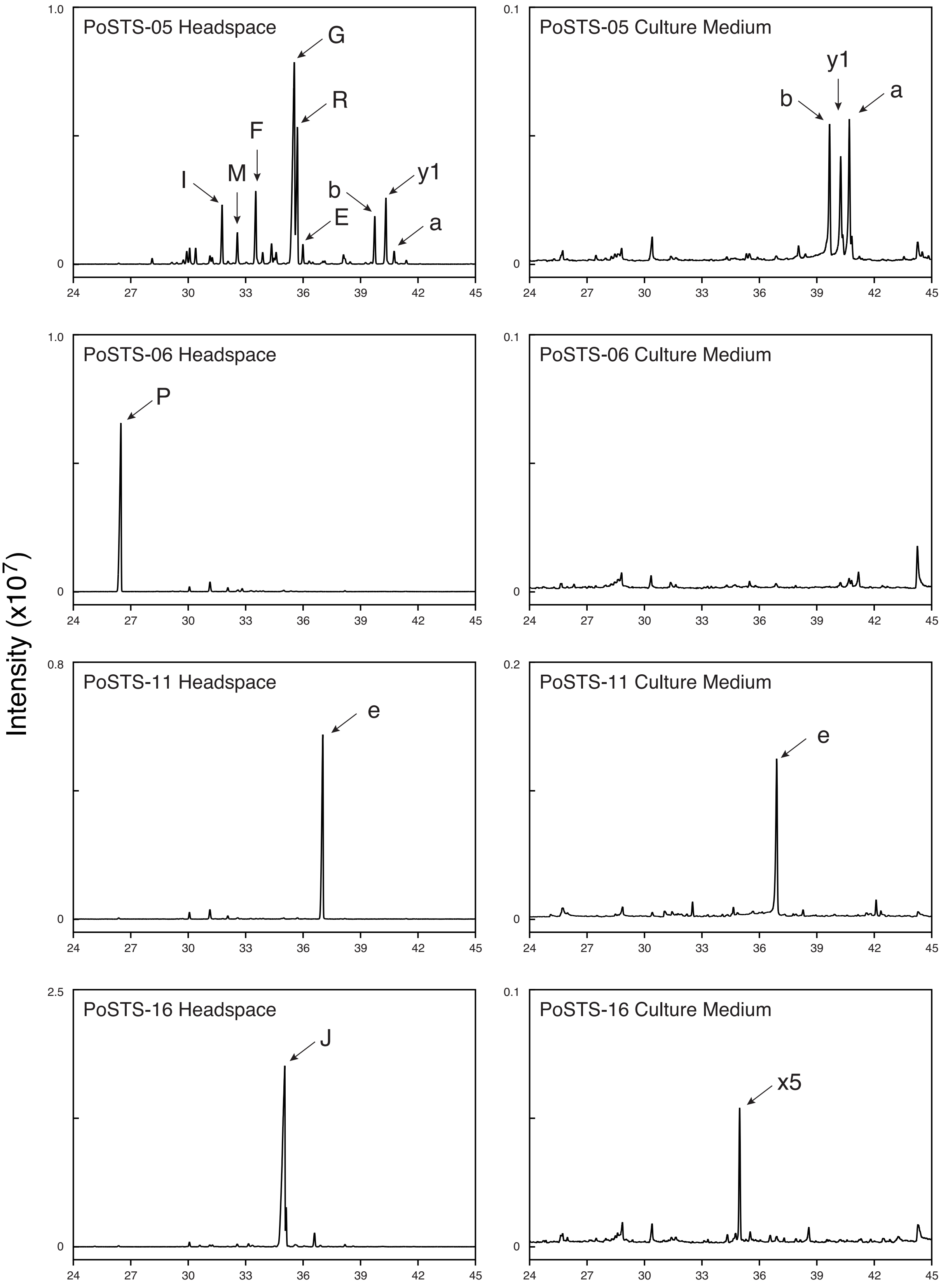

Fig. S3 Total ion chromatogram analysis of products by GC-MS (*continued*)

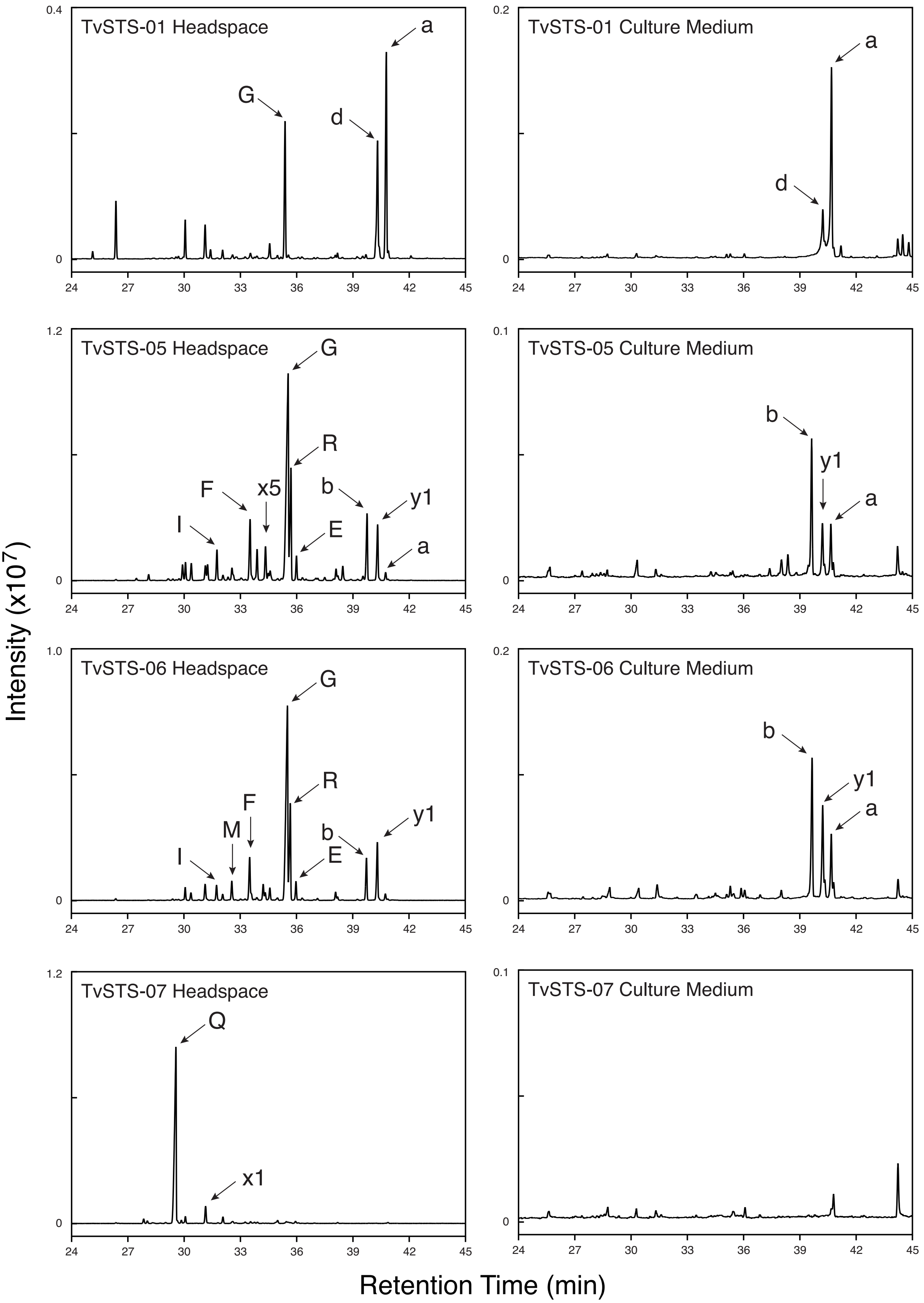

Fig. S3 Total ion chromatogram analysis of products by GC-MS (*continued*)

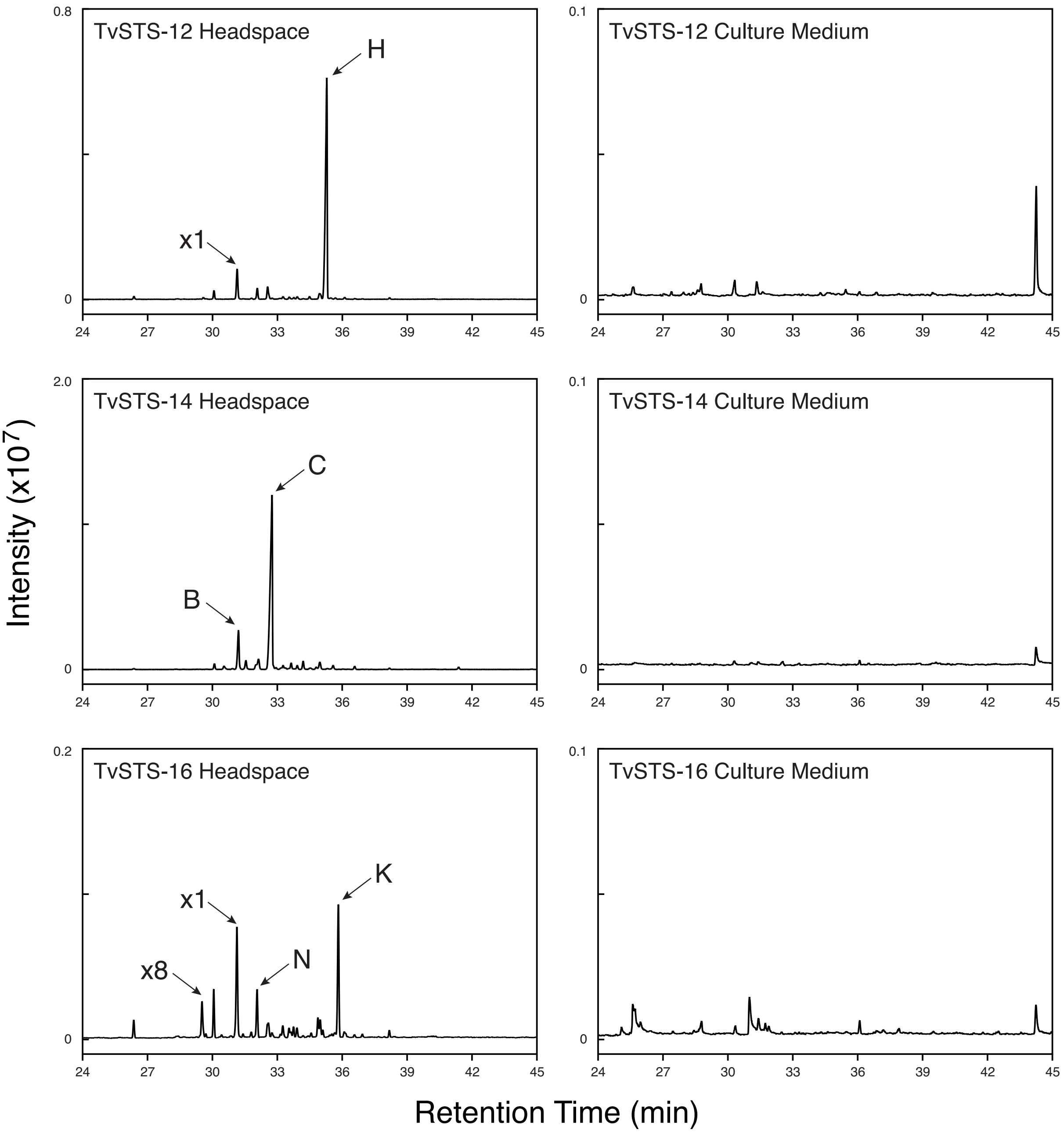

Fig. S3 Total ion chromatogram analysis of products by GC-MS (*continued*)

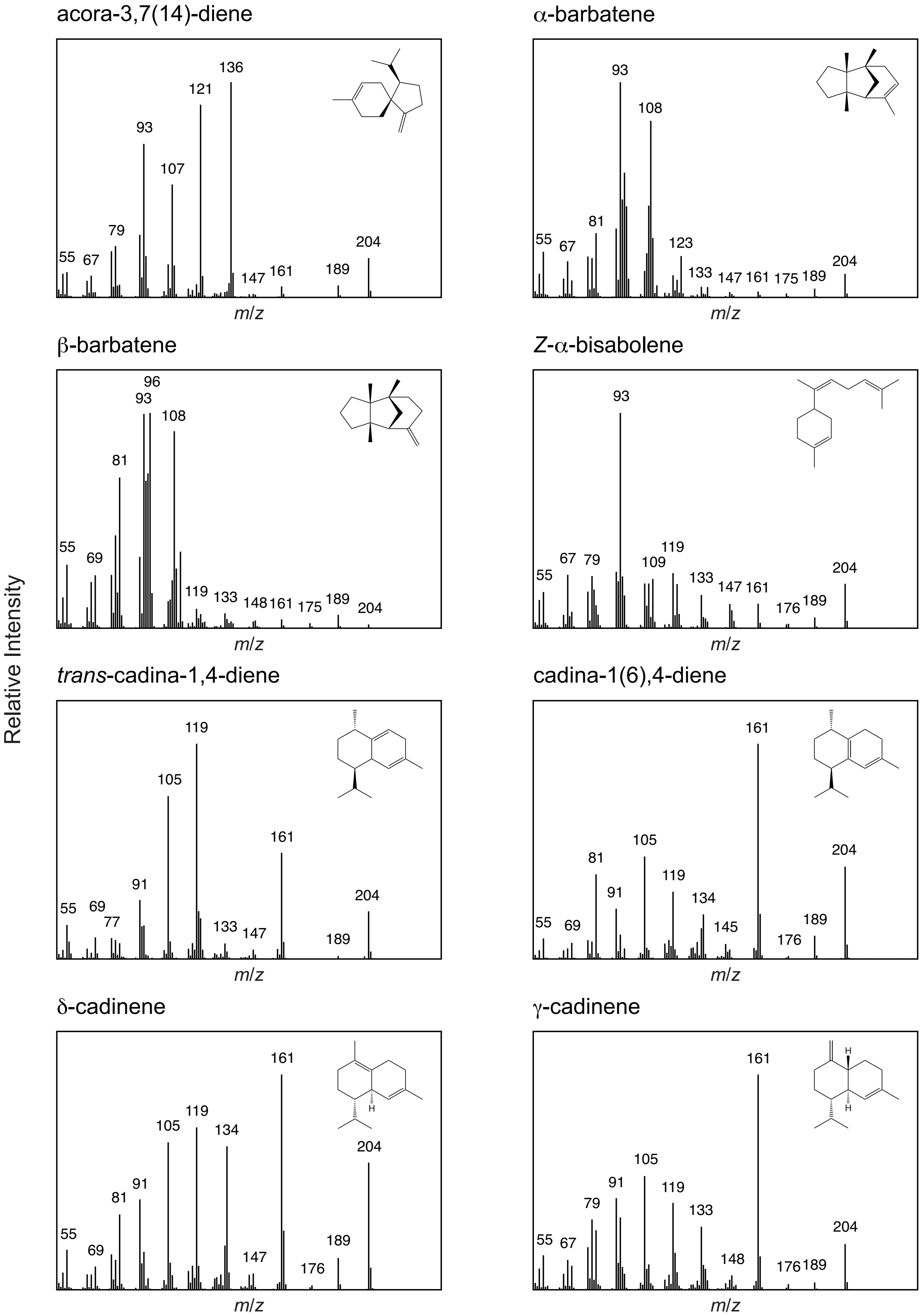

Fig. S4 Mass spectra of products synthesized by STSs

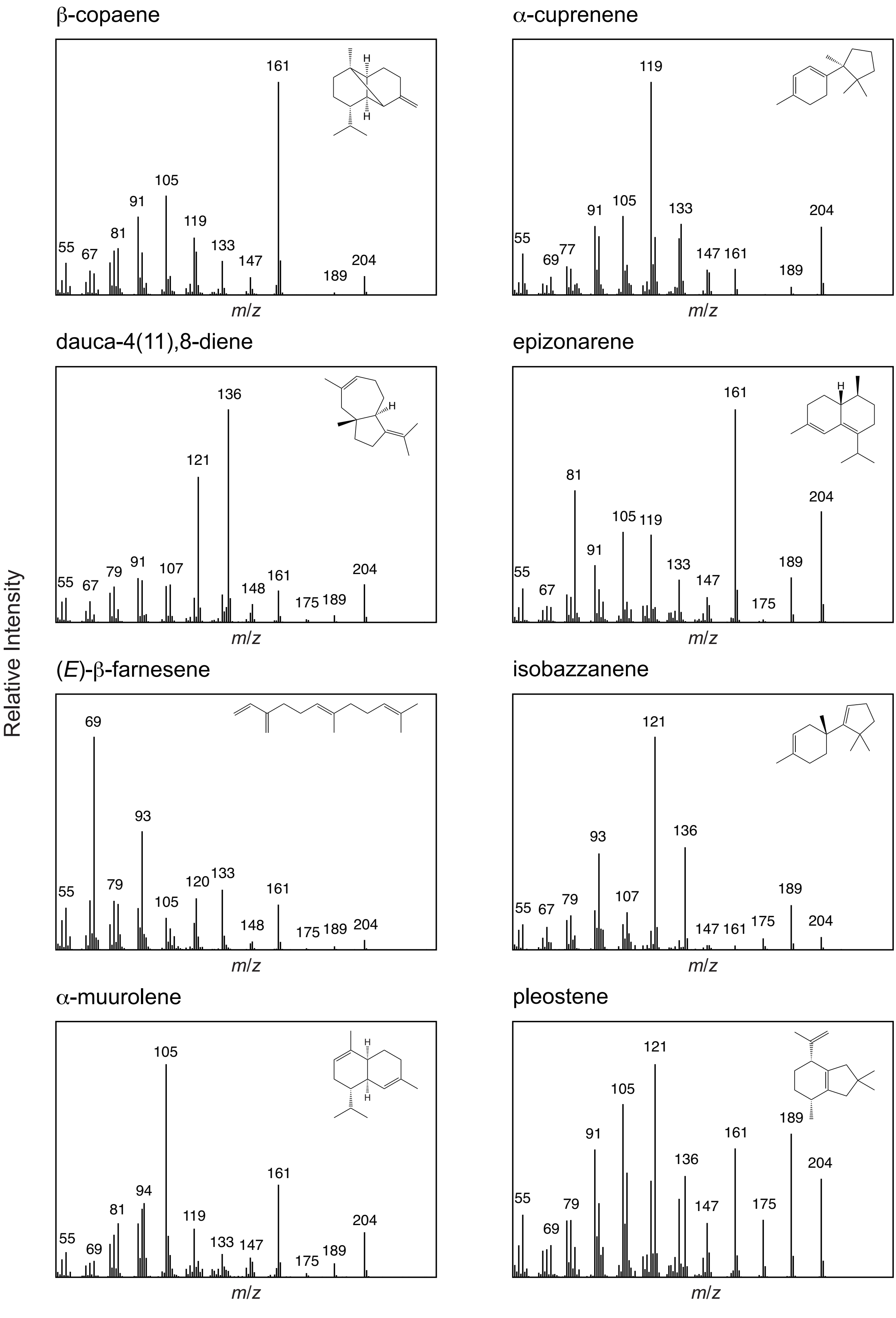

Fig. S4 Mass spectra of products synthesized by STSs (*continued*)

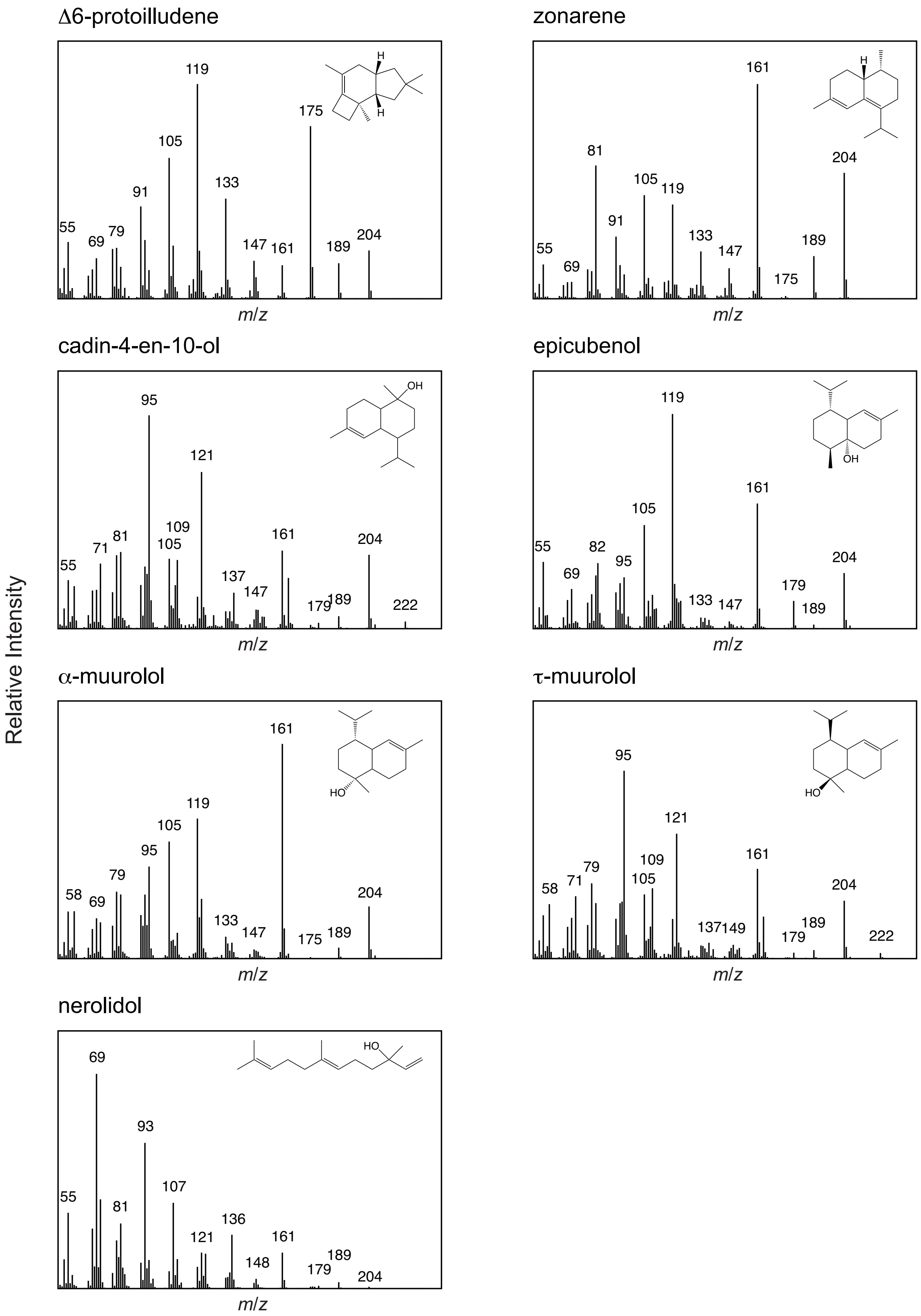

Fig. S4 Mass spectra of products synthesized by STSs (*continued*)

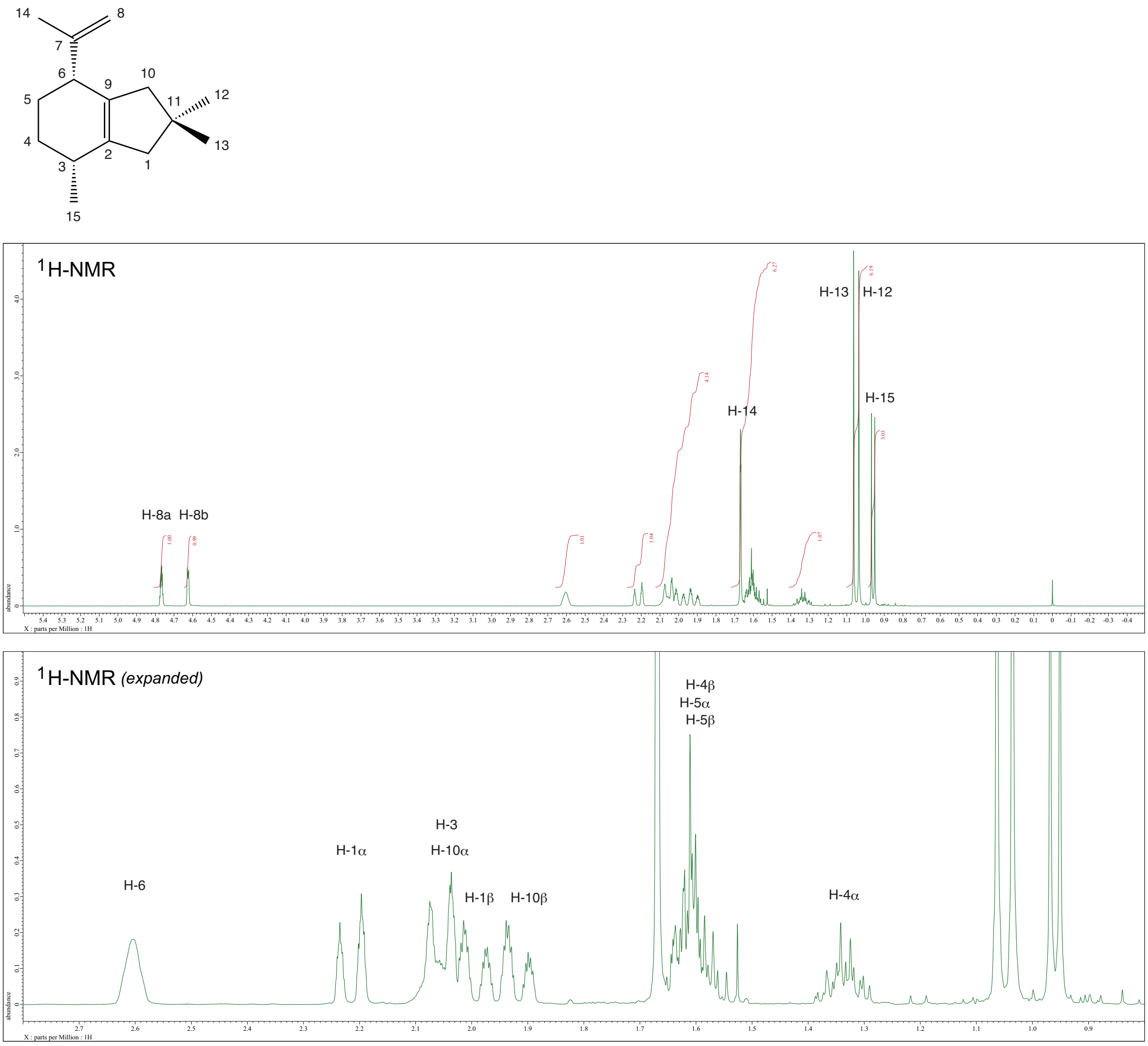

Fig. S5 ^{1}H-NMR spectra of pleostene synthesized by PoSTS-06

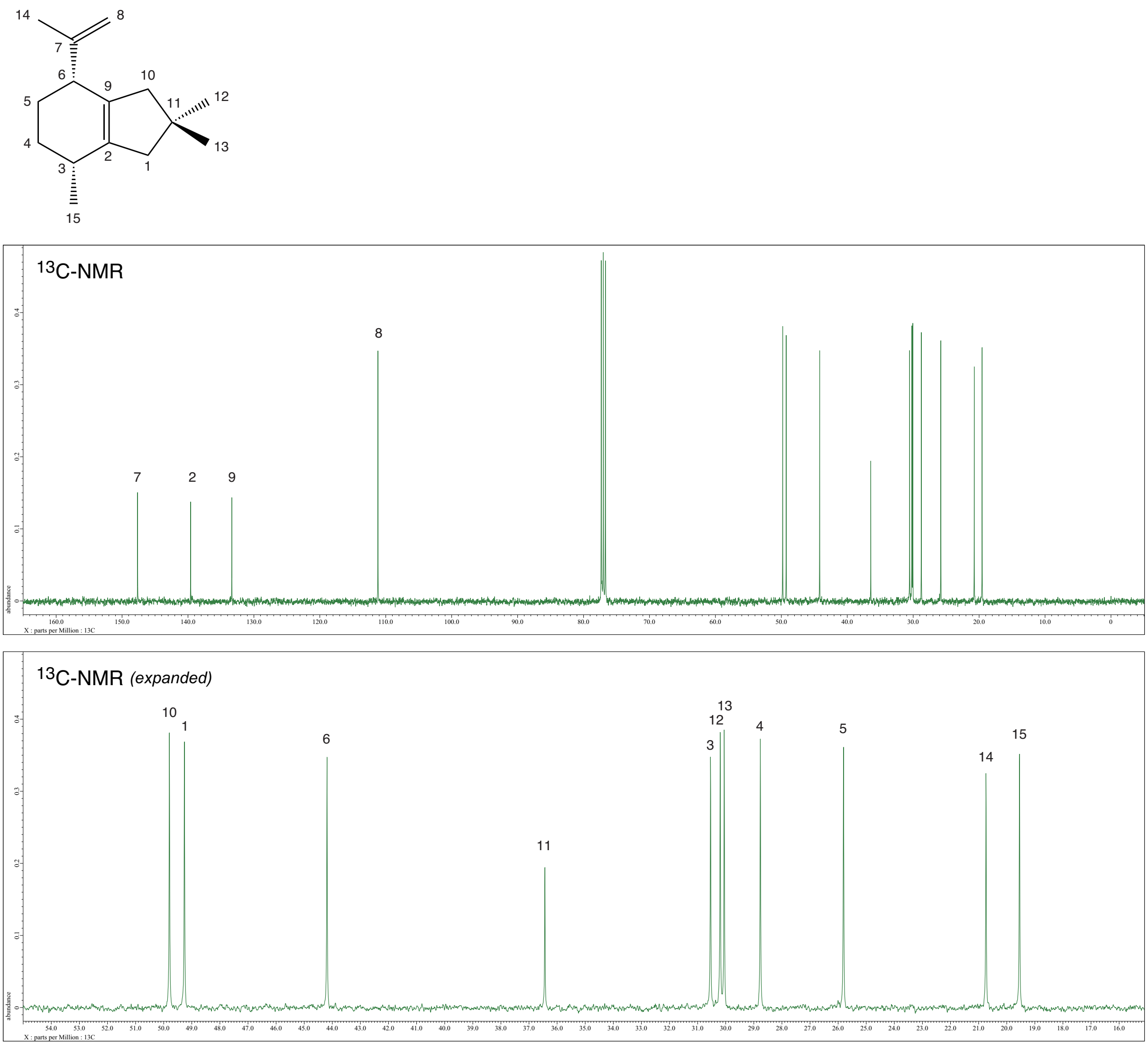

Fig. S6 ^{13}C-NMR spectrum of pleostene synthesized by PoSTS-06

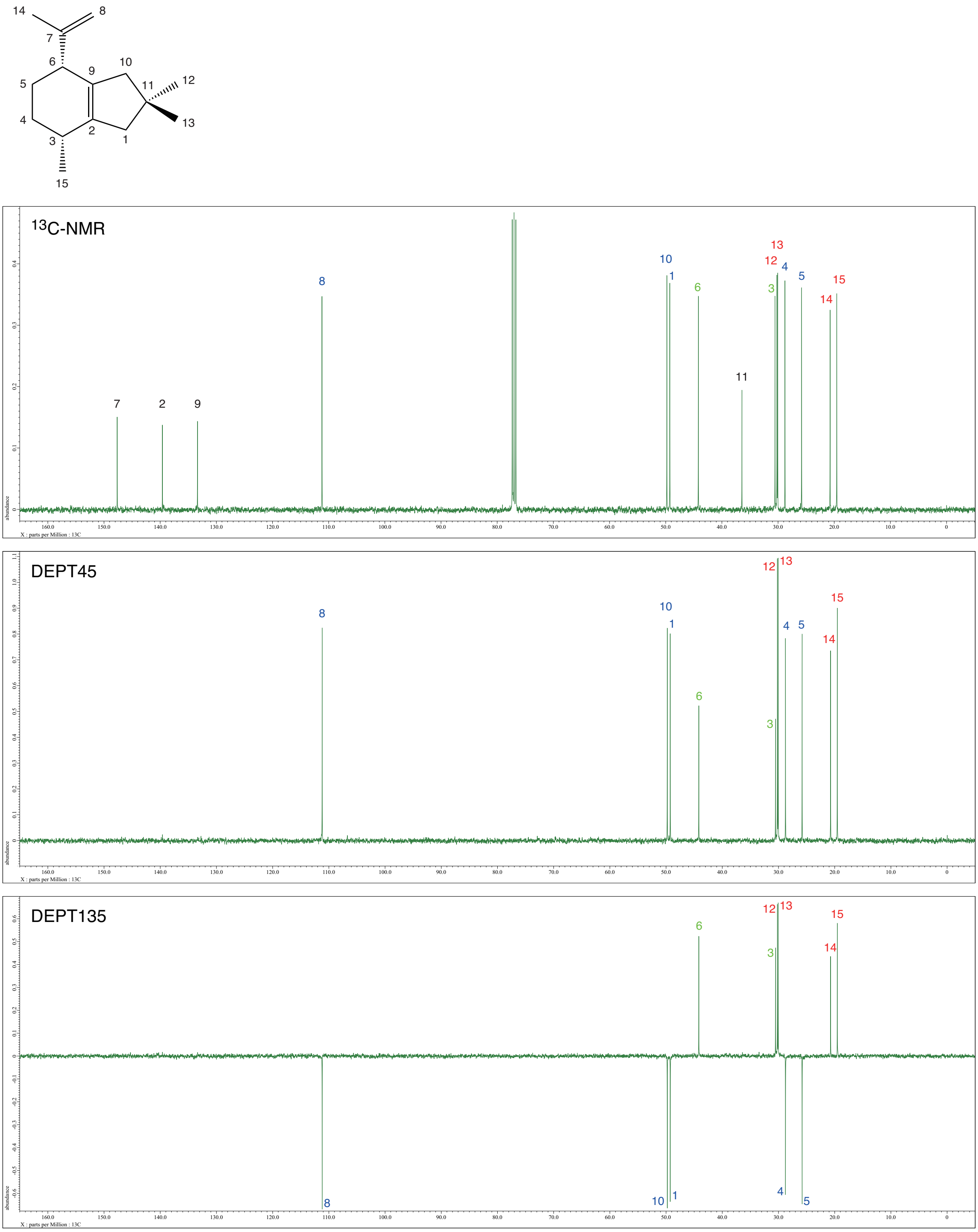

Fig. S7 DEPT ^{13}C-NMR spectra of pleostene synthesized by PoSTS-06

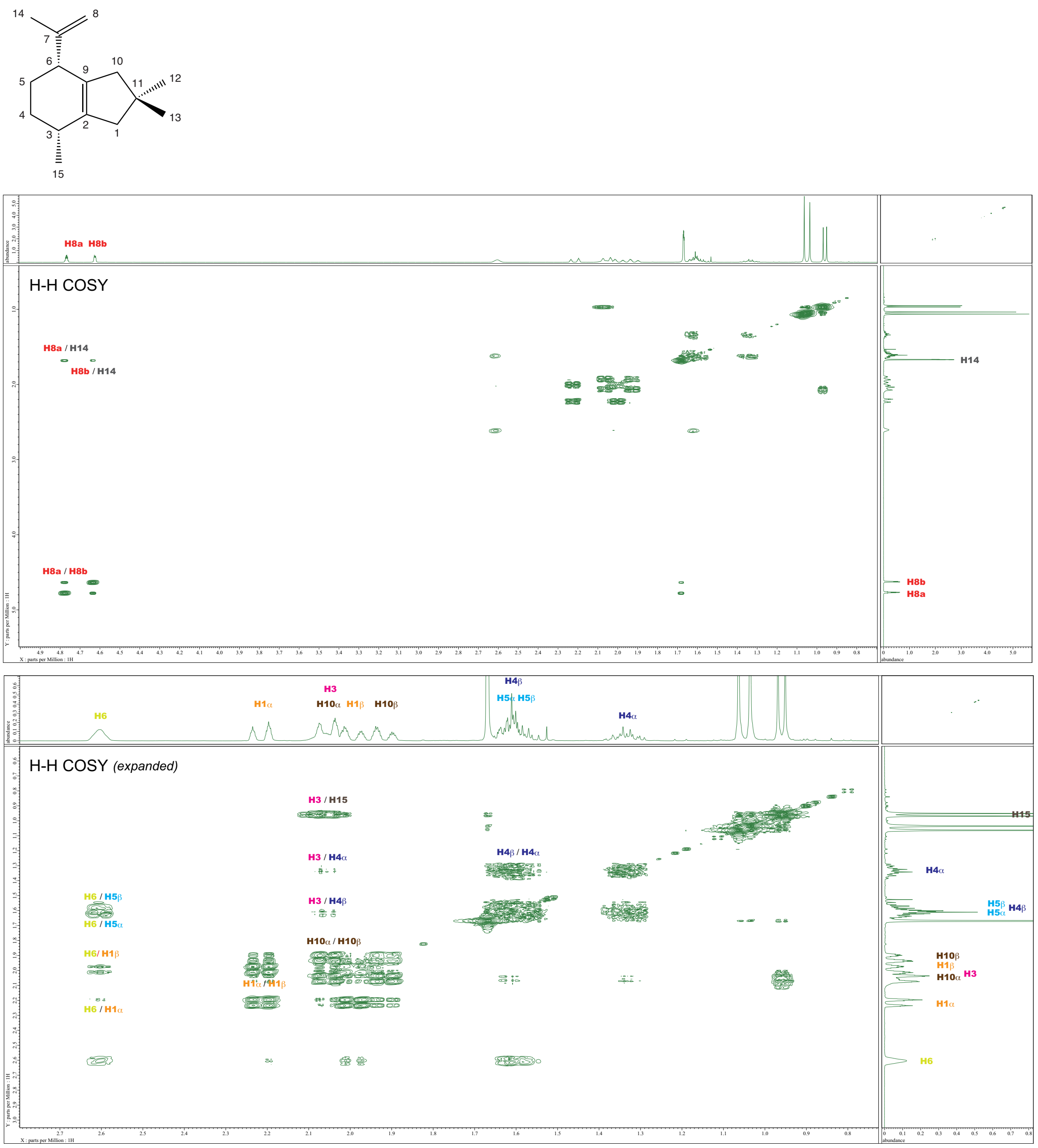

Fig. S8 H–H COSY spectrum of pleostene synthesized by PoSTS-06

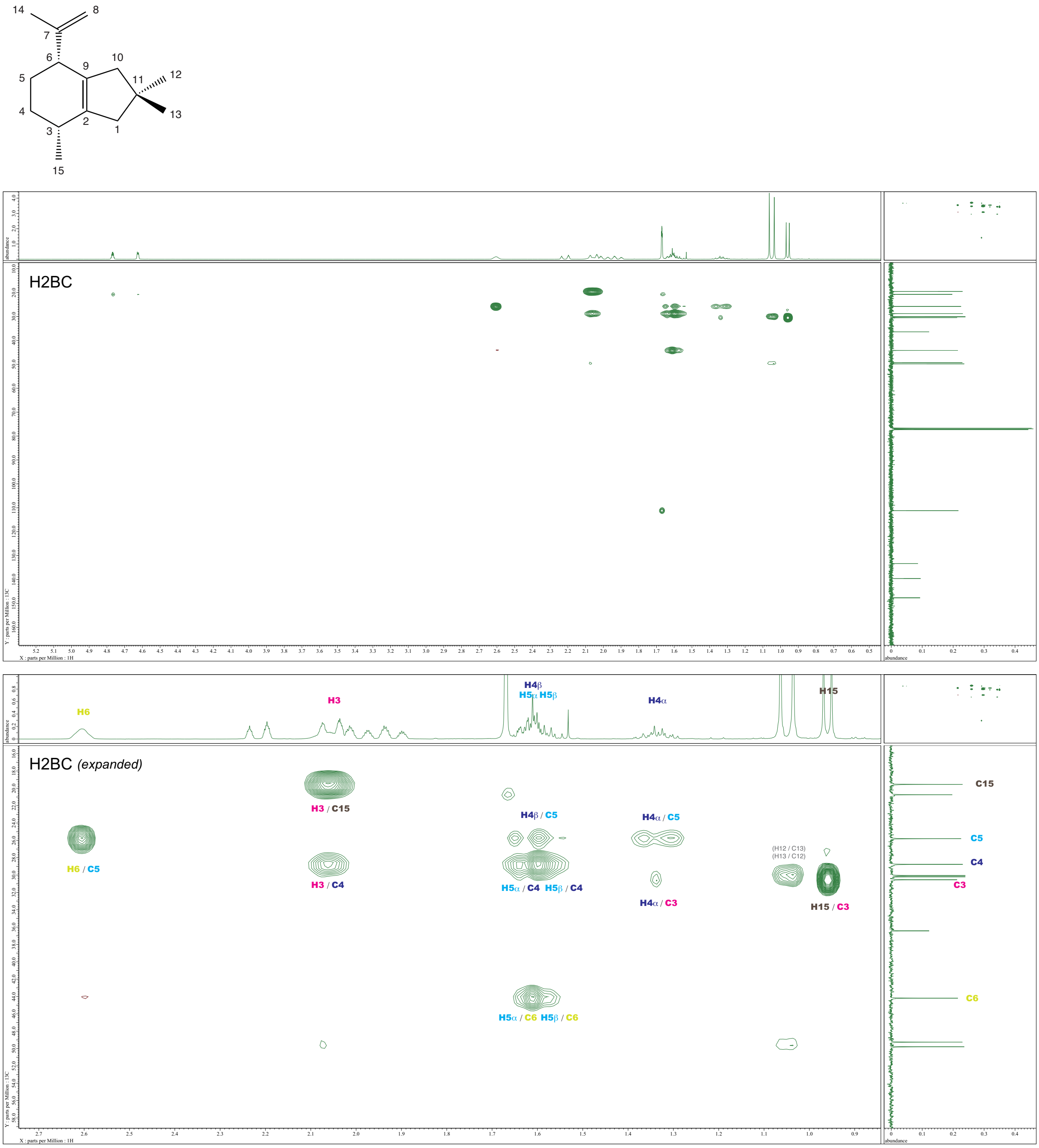

Fig. S9 H2BC spectrum of pleostene synthesized by PoSTS-06

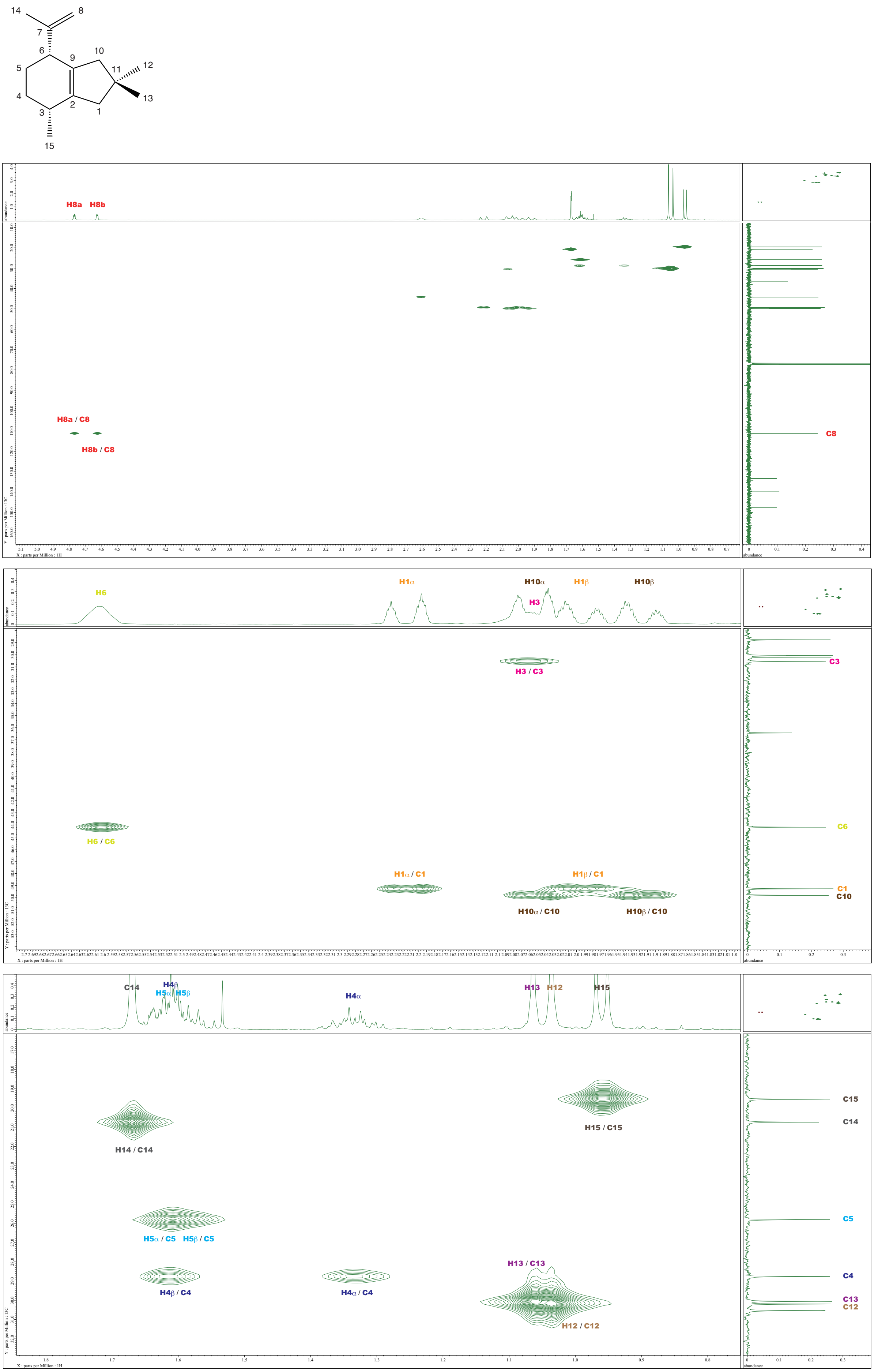

Fig. S10 HSQC spectrum of pleostene synthesized by PoSTS-06

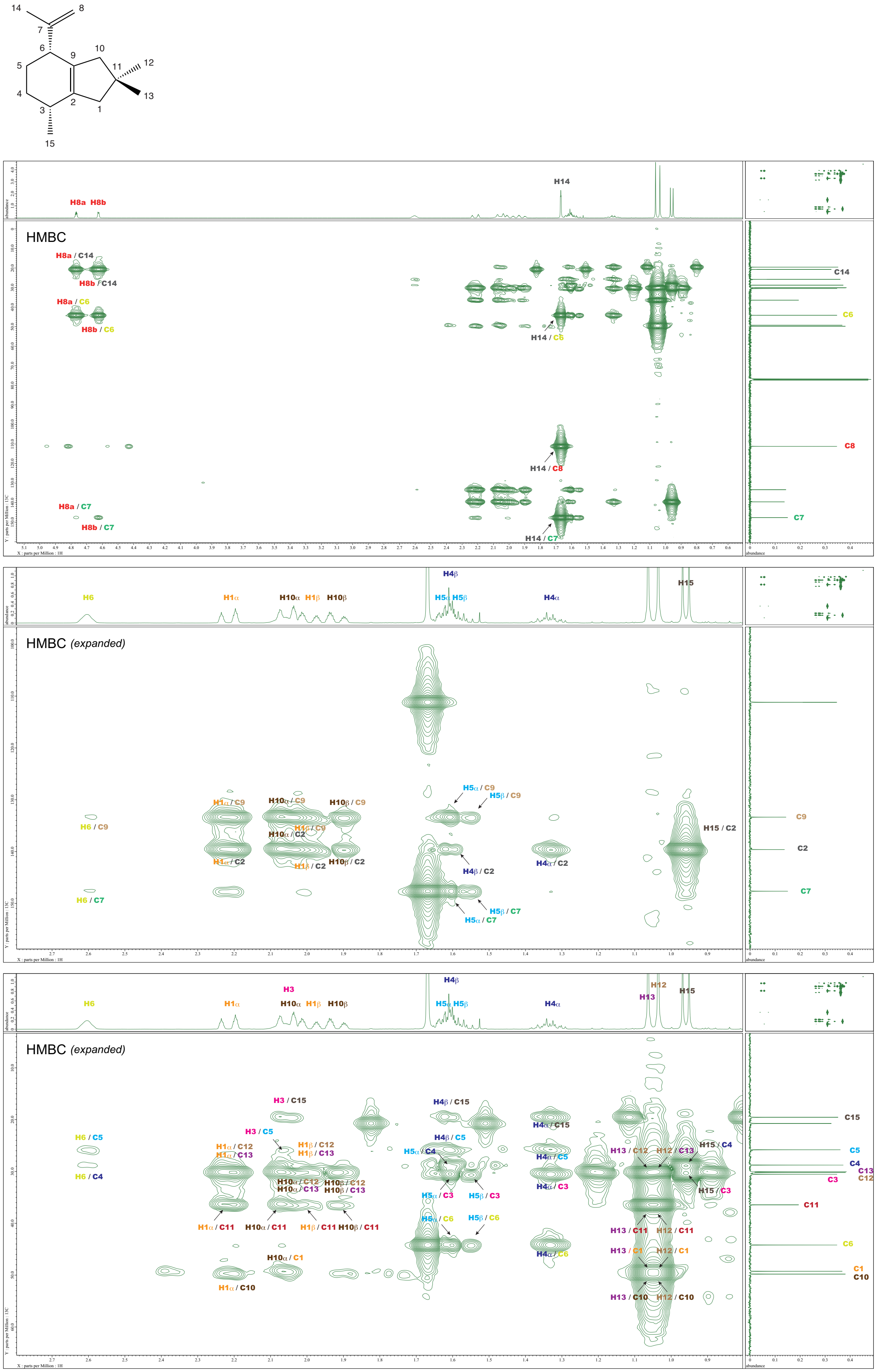

Fig. S11 HMBC spectrum of pleostene synthesized by PoSTS-06

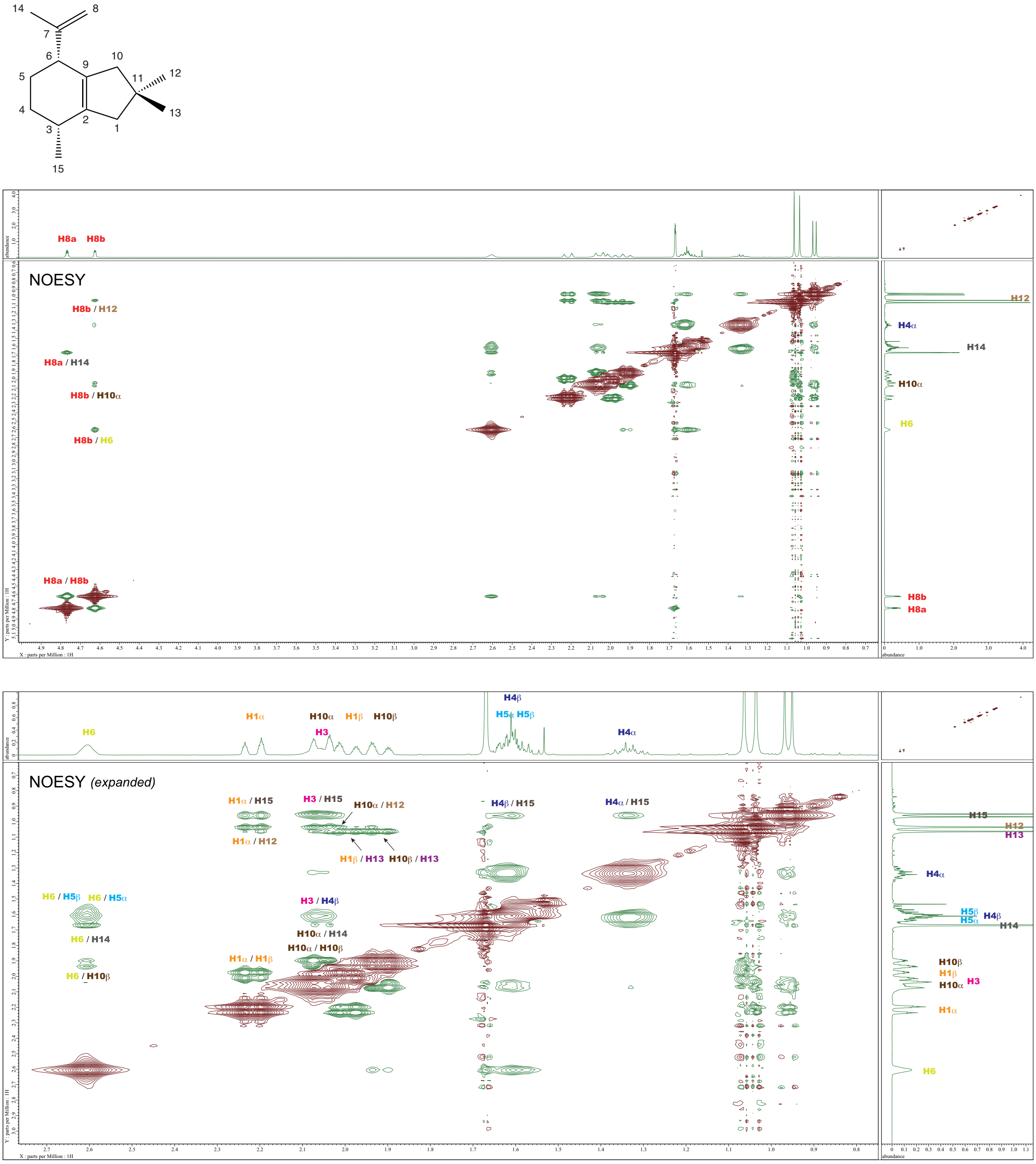

Fig. S12 NOESY spectrum of pleostene synthesized by PoSTS-06

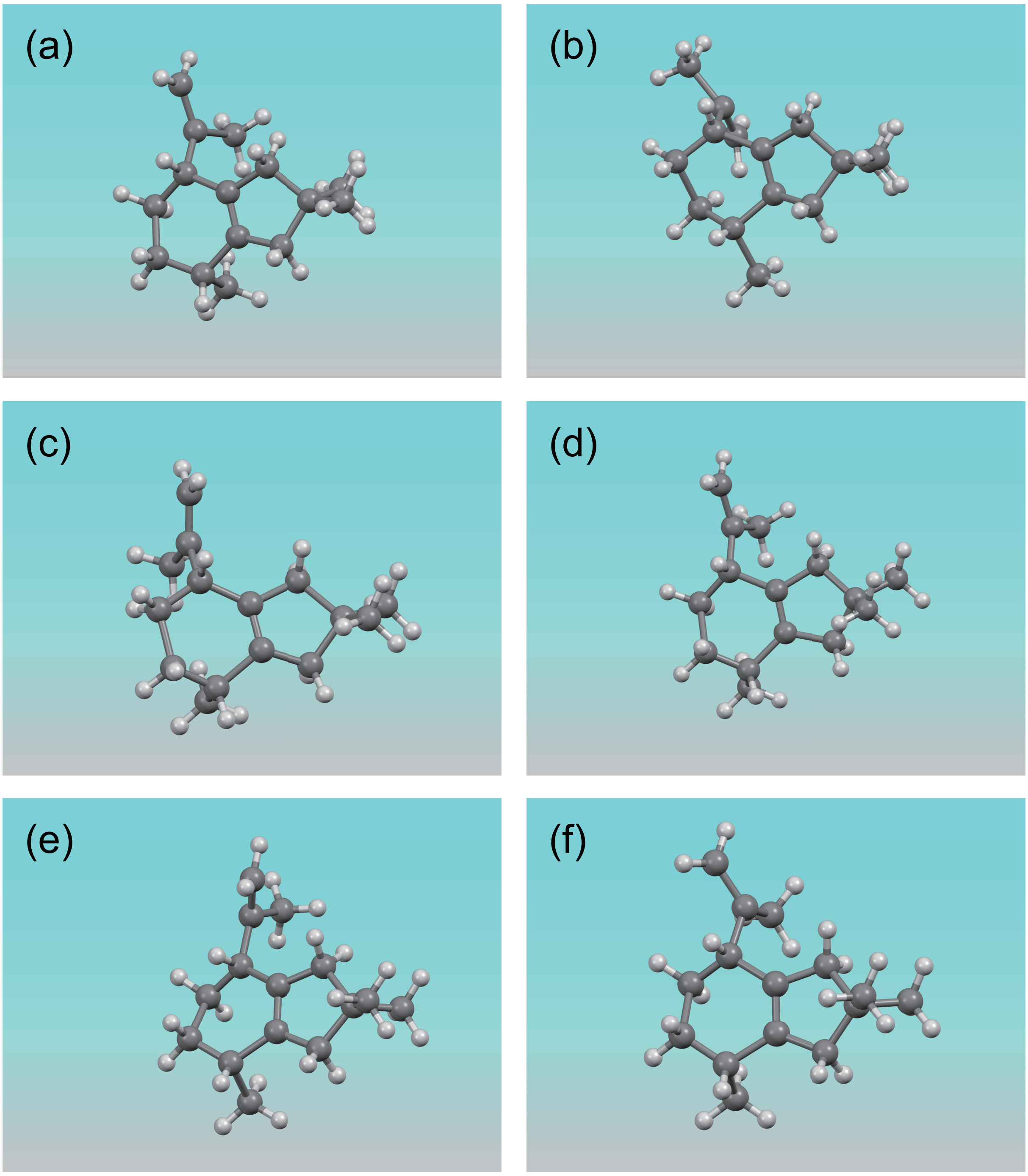

Fig. S13. Ball-and-stick diagrams for the crystal structure of guest molecules "a" to "f"

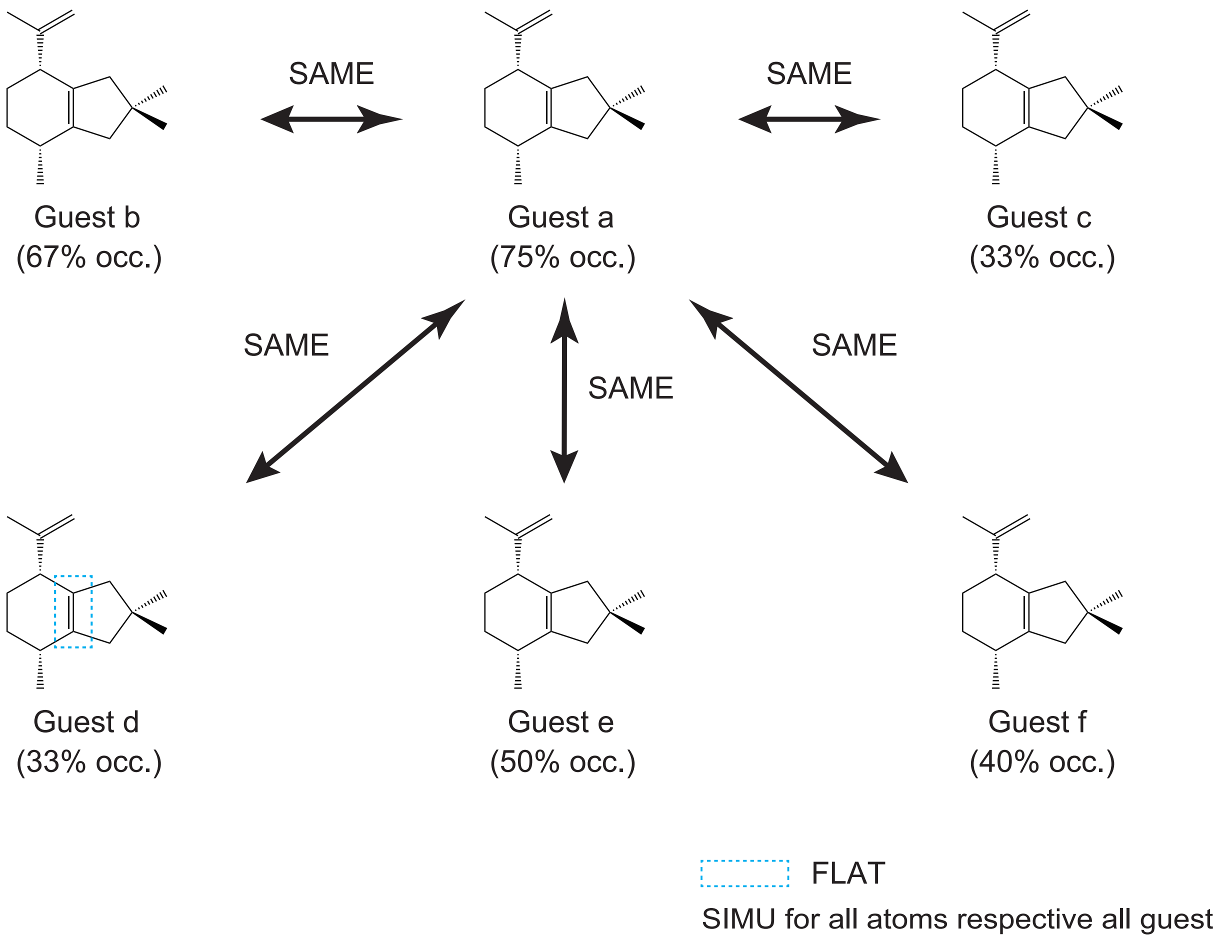

Fig. S14 Illustration representing restraints in pleostene

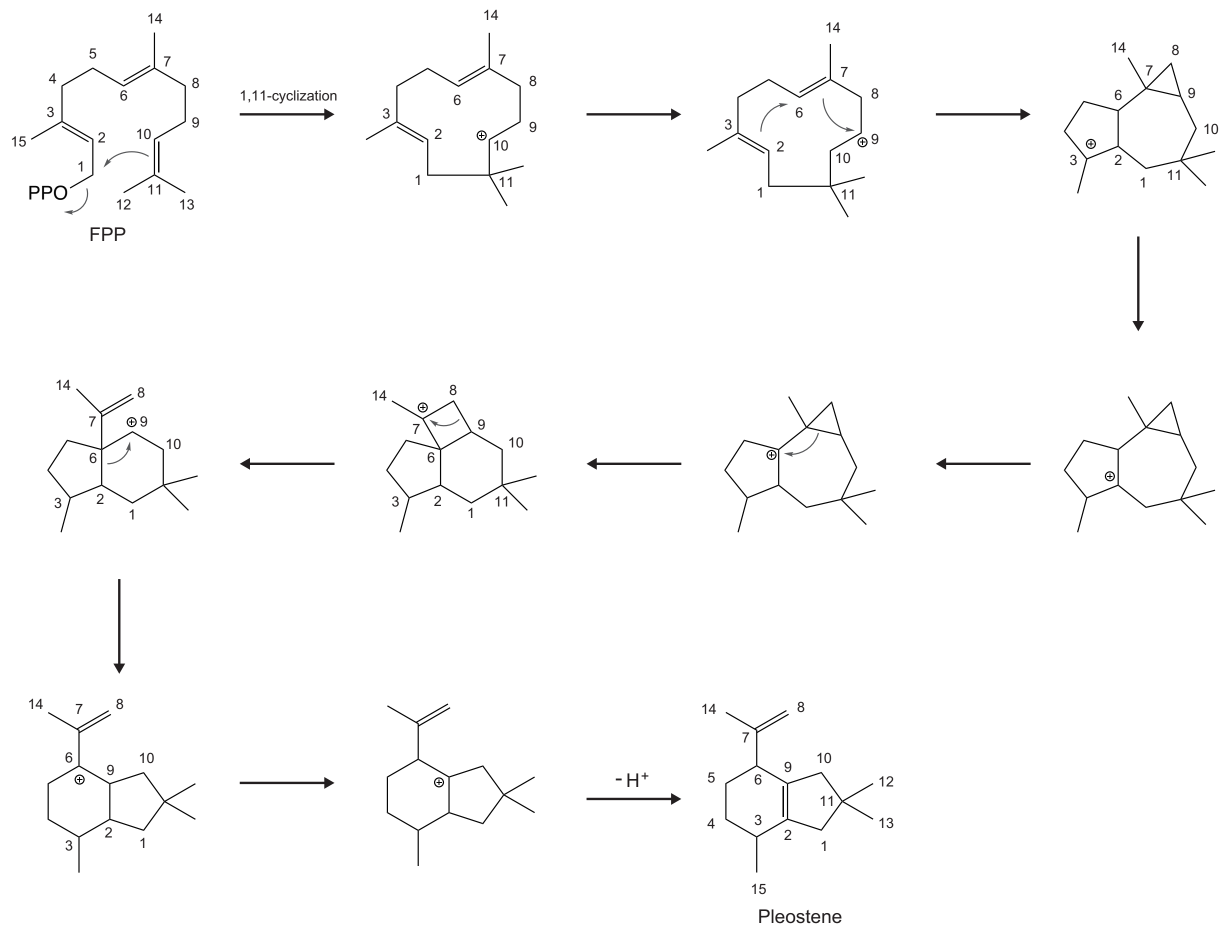

Fig. S15 Proposed reaction pathway for pleostene synthesis by PoSTS-06

Table S1 List of possible STSs genes

Name (Protein ID)	Organism	Location<br>Scaffold / position	Transcript*	Substitution**<br>NA	 AA	Accession<br>Number
AbSTS-01 (208101)	*Ag. bisporus*	8 / 1226112-1228096	Isolated	20	4	LC712878
AbSTS-02 (73543)	*Ag. bisporus*	8 / 1692342-1693815	NA	-	-	-
AbSTS-03 (183802)	*Ag. bisporus*	3 / 440073-441313	NA	-	-	-
AbSTS-04 (144791)	*Ag. bisporus*	8 / 1592201-1593665	NA	-	-	-
AbSTS-05 (149463)	*Ag. bisporus*	3 / 1350598-1351839	Isolated	7	2	LC712879
AbSTS-06 (195544)	*Ag. bisporus*	13 / 277596-279141	NA	-	-	-
AbSTS-07 (206522)	*Ag. bisporus*	6 / 2193942-2194794	Isolated	14	2	LC712880
AbSTS-08 (209957)	*Ag. bisporus*	10 / 1483405-1484256	NA	-	-	-
AbSTS-09 (119105)	*Ag. bisporus*	6 / 2189841-2190881	Isolated	41	11	LC712881
AvSTS-01 (1501365)	*Au. vulgare*	281 / 16074-17357	Isolated	115	18	LC712882
AvSTS-02 (1602279)	*Au. vulgare*	11 / 310031-311314	NA	-	-	-
AvSTS-03 (1579778)	*Au. vulgare*	6 / 26082-27343	Isolated	46	9	LC712883
AvSTS-04 (1641474)	*Au. vulgare*	28 / 124394-126049	Isolated	88	36	LC712884
AvSTS-05 (1556011)	*Au. vulgare*	28 / 54585-55920		-	-	-
AvSTS-06 (1380955)	*Au. vulgare*	9 / 208755-210324	Isolated	79	18	LC712885
AvSTS-07 (1480961)	*Au. vulgare*	2 / 130377-131688	Isolated	68	22	LC712886
AvSTS-08 (1560965)	*Au. vulgare*	109 / 523-2419	FS	-	-	-
AvSTS-09 (1602471)	*Au. vulgare*	24 / 45705-46825	Isolated	52	9	LC712887
AvSTS-10 (1676885)	*Au. vulgare*	27 / 31413-32554	FS	-	-	-
AvSTS-11 (1555884)	*Au. vulgare*	27 / 42112-43872	Isolated	58	15	LC712888
AvSTS-12 (22560)	*Au. vulgare*	1 / 318853-320684	Isolated	111	40	LC712889
AvSTS-13 (1582934)	*Au. vulgare*	82 / 15316-16591	Isolated	99	36	LC712890
AvSTS-14 (1603700)	*Au. vulgare*	27 / 34611-35752	NA	-	-	-
LnSTS-01 (1262599)	*L. nuda*	49 / 229600-231257	Isolated	32	10	LC712891
LnSTS-02 (1163589)	*L. nuda*	32 / 242575-243967	Isolated	38	11	LC712892
LnSTS-03 (1178987)	*L. nuda*	271 / 10053-11376	Isolated	15	4	LC719125
LnSTS-04 (1377465)	*L. nuda*	71 / 128964-130585	Isolated	32	12	LC719126
LnSTS-05 (1319989)	*L. nuda*	17 / 19936-21900	NA	-	-	-
LnSTS-06 (1306770)	*L. nuda*	17 / 97173-98353	NA	-	-	-
LnSTS-07 (296758)	*L. nuda*	17 / 38180-40135	Isolated	42	6	LC712893

*NA, not amplified by PCR; FS, frame-shifted cDNA. **Number of nucleotide and amino acid substitutions between strain H97 and NBRC31861 of *Ag. bisporus*, FP105234-Sp and NBRC30159 of *Au. vulgare*, CBS 247.69 and NBRC30380 of *L. nuda*, PC15 and NBRC104981 of *P. ostreatus*, and MB281625 and NBRC30340 of *T. versicolor*.

Table S1 List of possible STSs genes (*continued*)

Name (Protein ID)	Organism	Location<br>Scaffold / position	Transcript*	Substitution**<br>NA	 AA	Accession<br>Number
LnSTS-08 (377423)	*L. nuda*	2 / 595200-596832	Isolated	25	5	LC712894
LnSTS-09 (1306134)	*L. nuda*	13 / 239652-240907	Isolated	32	4	LC712895
LnSTS-10 (902535)	*L. nuda*	7 / 110573-112106	Isolated	29	5	LC712896
LnSTS-11 (1306811)	*L. nuda*	49 / 229600-231257	NA	-	-	-
LnSTS-12 (1238681)	*L. nuda*	32 / 242575-243967	NA	-		-
LnSTS-13 (1313569)	*L. nuda*	271 / 10053-11376	NA	-	-	-
LnSTS-14 (1313570)	*L. nuda*	71 / 128964-130585	NA	-	-	-
LnSTS-15 (1296610)	*L. nuda*	17 / 19936-21900	NA	-	-	-
LnSTS-16 (1365201)	*L. nuda*	17 / 97173-98353	FS	-	-	-
LnSTS-17 (1309792)	*L. nuda*	17 / 38180-40135	NA	-	-	-
LnSTS-18 (1186072)	*L. nuda*	2 / 595200-596832	Isolated	40	11	LC712897
LnSTS-19 (1313453)	*L. nuda*	13 / 239652-240907	Isolated	26	3	LC712898
LnSTS-20 (1306686)	*L. nuda*	7 / 110573-112106	Isolated	28	6	LC712899
LnSTS-21 (1234264)	*L. nuda*	16 / 319380-320443	Isolated	33	12	LC712900
LnSTS-22 (1153963)	*L. nuda*	4 / 506043-506682	NA	-	-	-
LnSTS-23 (1275887)	*L. nuda*	205 / 22702-24437	NA	-	-	-
LnSTS-24 (280020)	*L. nuda*	16 / 292048-293010	FS	-	-	-
LnSTS-25 (1137587)	*L. nuda*	102 / 44165-45328	Isolated	40	13	LC712901
LnSTS-26 (146147)	*L. nuda*	121 / 19215-20687	NA	-	-	-
LnSTS-27 (1317956)	*L. nuda*	5 / 229767-231365	Isolated	24	5	LC712902
PoSTS-01 (1061909)	*P. ostreatus*	2 / 605190-607215	Isolated	14	2	LC712903
PoSTS-02 (1046456)	*P. ostreatus*	8 / 2048717-2049878	Isolated	30	7	LC712904
PoSTS-03 (1060726)	*P. ostreatus*	1 / 1678589-1680809	Isolated	14	4	LC712905
PoSTS-04 (1106708)	*P. ostreatus*	7 / 1884217-1885482		-	-	-
PoSTS-05 (1047598)	*P. ostreatus*	8 / 1036808-1037986	Isolated	27	3	LC712906
PoSTS-06 (1098067)	*P. ostreatus*	9 / 1755062-1756402	Isolated	31	4	LC712907
PoSTS-07 (1039734)	*P. ostreatus*	4 / 3009361-3016326	NA	-	-	-
PoSTS-08 (1041418)	*P. ostreatus*	5 / 3490684-3491857	NA	-	-	-
PoSTS-09 (1069096)	*P. ostreatus*	11 / 2572809-2574153	Isolated	54	18	LC719127
PoSTS-10 (160242)	*P. ostreatus*	7 / 1362220-1363490	NA	-	-	-

Table S1 List of possible STSs genes (*continued*)

Name (Protein ID)	Organism	Location<br>Scaffold / position	Transcript*	Substitution**<br>NA	 AA	Accession<br>Number
PoSTS-11 (161354)	*P. ostreatus*	8 / 1262645-1263897	Isolated	56	25	LC712908
PoSTS-12 (1073415)	*P. ostreatus*	1 / 1791615-1792707	NA	-	-	-
PoSTS-13 (155013)	*P. ostreatus*	3 / 1263125-1264247	NA	-	-	-
PoSTS-14 (50572)	*P. ostreatus*	4 / 2985652-2988092	NA	-	-	-
PoSTS-15 (1049659)	*P. ostreatus*	10 / 629334-629921	NA	-	-	-
PoSTS-16 (1048495)	*P. ostreatus*	9 / 1314039-1315120	Isolated	38	6	LC712909
PoSTS-17 (30147)	*P. ostreatus*	6 / 1415567-1416616	NA	-	-	-
TvSTS-01 (30977)	*T. versicolor*	10 / 1559291-1563385	Isolated	23	4	LC712910
TvSTS-02 (75578)	*T. versicolor*	16 / 334993-336764	NA	-	-	-
TvSTS-03 (125681)	*T. versicolor*	7 / 1111138-1112353	NA	-	-	-
TvSTS-04 (124930)	*T. versicolor*	7 / 1124066-1125289	Isolated	17	1	LC712911
TvSTS-05 (169091)	*T. versicolor*	7 / 749823-751279	Isolated	27	1	LC712912
TvSTS-06 (20994)	*T. versicolor*	7 / 744722-745858	Isolated	23	3	LC712913
TvSTS-07 (122204)	*T. versicolor*	5 / 2758363-2759604	Isolated	41	7	LC712914
TvSTS-08 (119121)	*T. versicolor*	4 / 23363-24599	NA	-	-	-
TvSTS-09 (35003)	*T. versicolor*	3 / 1164870-1166118	Isolated	47	20	LC712915
TvSTS-10 (118176)	*T. versicolor*	3 / 1162644-1163847	Isolated	37	5	LC712916
TvSTS-11 (37122)	*T. versicolor*	5 / 2737272-2738797	FS	-	-	-
TvSTS-12 (21857)	*T. versicolor*	8 / 2056336-2058096	Isolated	21	4	LC712917
TvSTS-13 (174695)	*T. versicolor*	13 / 486019-487486	Isolated	27	0	LC712918
TvSTS-14 (47026)	*T. versicolor*	5 / 2177847-2179041	Isolated	53	15	LC712919
TvSTS-15 (47002)	*T. versicolor*	5 / 2099870-2101117	FS	-	-	-
TvSTS-16 (47003)	*T. versicolor*	5 / 2102670-2104097	Isolated	44	16	LC712920
TvSTS-17 (44143)	*T. versicolor*	3 / 560772-561902	FS	-	-	-
TvSTS-18 (167198)	*T. versicolor*	5 / 2088657-2090240	NA	-	-	-
TvSTS-19 (19935)	*T. versicolor*	5 / 1661297-1662536	NA	-	-	-

Table S2 Basidiomycetous STSs used for phylogenetic analysis

Gene	Accession Number / Protein ID	Gene	Accession Number / Protein ID
Cop1	XP_001832573	PpSTS-14	LC378434
Cop2	XP_001836556	PpSTS-15	54222
Cop3	XP_001832925	PpSTS-16	LC378435
Cop4	XP_001836356	PpSTS-17	101570
Cop5	XP_001834007	PpSTS-20	97981
Cop6	XP_001832549	PpSTS-21	106438
Omp1	MUStwsD_GLEAN_10001317	PpSTS-22	LC378437
Omp2	MUStwsD_GLEAN_10002575	PpSTS-23	91093
Omp3	MUStwsD_GLEAN_10003938	PpSTS-24	LC378438
Omp4	MUStwsD_GLEAN_10005581	PpSTS-25	LC378439
Omp5a	MUStwsD_GLEAN_10000810	PpSTS-26	95481
Omp5b	MUStwsD_GLEAN_10000811	PpSTS-27	125960
Omp6	MUStwsD_GLEAN_10003820	PpSTS-29	LC378440
Omp7	MUStwsD_GLEAN_10000831	PpSTS-30	101756
Omp8	MUStwsD_GLEAN_10000534	PcSTS-01	LC634451
Omp9	MUStwsD_GLEAN_10000543	PcSTS-02	LC634452
Omp10	MUStwsD_GLEAN_10000292	PcSTS-03	LC634453
PpSTS-01	LC378425	PcSTS-04	LC634454
PpSTS-03	LC378427	PcSTS-05	1815
PpSTS-05	87954	PcSTS-06	LC634455
PpSTS-06	LC378428	PcSTS-07	LC634456
PpSTS-07	LC378429	PcSTS-08	LC634457
PpSTS-08	LC378430	PcSTS-09	LC634458
PpSTS-09	LC378431	PcSTS-10	768
PpSTS-10	LC378432	PcSTS-11	LC634459
PpSTS-13	LC378433		

The protein IDs of PpSTSs and PcSTSs were matched with the database for *Postia placenta* (https://mycocosm.jgi.doe.gov/Pospl1/Pospl1.home.html) and *Phanerochaete chrysosporium* (http://genome.jgi.doe.gov/Phchr1/Phchr1.home.html), respectively.

Table S3 List of primers used for cDNA isolation

Target	Nucleotide sequence (5'-3')			
	1st round PCR		Nucleotide sequence	
	Forward Primer	Reverse Primer	Forward Primer	Reverse Primer
AbSTS-01	TTTTCTCGTCACTACCTCTTCA	AAAGACTAAAAAGACGAGGAGAGAT	ATGGCTTTTGCAGTCGCTTC	TTACAGATACCCGTAGTGACAGAG
AbSTS-02	CTTGTACTTCTTTCATTCTCTAGTGTATC	GAGAGAGATGTGTGTGGTTTTAAAAA	ATGGGTCGTGCTCAACTTAG	TTAGAGAGGAATGGATATAGTAGATCTTGAG
AbSTS-03	TCGCATTCGTCGCCGCAACA	ACGAAAATGAAATAGGGATAACTTAGGTT	ATGCCCAACCAGATGGTCTT	TCAAGCCTGAAAAACGACAAGG
AbSTS-04	CACTTGTACTTCTTTCATTCTATTGTGTATC	CAAAGAAAGGAAAAGATTTTTCGGAAATTA	ATGAGTTACGCTCAGCTTAAACAAG	TCAGAAACTAATATGGTCGATCTTGAAAG
AbSTS-05	CACACTACTACCTCAATACCTACG	GAAAGATGAGAGAGAATATGCGTTC	ATGCCTCGCCCTCAACAATT	TCACTCGCAATCTGAATCTGAGTC
AbSTS-06	CTCCTCTATCACTTGTCTACTATCATATATC	CAAATTAATAGATTCAAATACTAATTATATACAGCCA	ATGGTTCAAACCTCCGCTCA	TTATACCAACAACTCATTTGTATGTGTATAC
AbSTS-07	GACCGCCGCGGCTACGAC	GCAGGTCTGTTCAACGAGAT	ATGCACTATCTTTCGAAGCATCT	CTAATGAATGCCTAGCTCCATGA
AbSTS-08	CTTATCGTCGCGGCTACGAT	TTGGGCTATATGATCCATTCAATGATAT	ATGCACTATCTTTCGAAACATCTCCA	CTATTGAATGCCTAACTCCATGAGG
AbSTS-09	TTTGTATCTGCTTTTCACTGCC	GATGCTGCGTTCAATGTGAT	ATGGGAGCCGTTGGACAACT	CTAGTTAAAGCCTAGCTCCATGAGG
AvSTS-01	CGCTTCAATCGCCACCCTACT	GCCTAAAATGTTTCTCCGACGTTCGAG	ATGCAGTCGCCAACGCAAG	TCAAACCATCAACTCCTGCAGCG
AvSTS-02	CTGAATCAACCGCTTGCCCTACT	CGAAGATGTGCACGATCTACAACTTCC	ATGCAGTCGCCAACGCAAG	TTAGATCATCAACTCCTGCAGCGG
AvSTS-03	ACCTACTCAATTGCCACCGCC	CGAAGATGTGCACGATCTACAACTTCC	ATGCCTGAGAAGTTCTACATCCCTAACT	CTACAACATGGAACCGTCCACGAT
AvSTS-04	GCTTCAATCGCCACCCCACT	AGGCTCCGACGTCAGACCA	ATGCAATCACCAACGCAAGATTCTTATC	TCAACGTTTCAACTCCAGCAGC
AvSTS-05	CCATCCAACTTCCATGGCAGCA	GACATCTAGCAATCGAAGCAAACTATCAGTAAT	ATGTCGCCAATACCGCTTTCTTACA	CTACACAATCGGGCGTCCCGA
AvSTS-06	GCGGCGCTCTCTCCTCTC	CTATAGCACACATGTCTAACTCTACACACAATAAG	ATGTCCACCCAAATCCAATTCCGC	TTACAGGTACATCCAACGGCCC
AvSTS-07	TCCTCCTCTCCGAACAATCCAAC	CTCTCAGTCGCATAGGGCGATTC	ATGTCCACCCAAATCCAATTCCGC	TCAAACCACCACCGAATTCCCC
AvSTS-08	TCTCCACTCGCCGCTACC	ATCATCCCAAGGTACATCCCGC	ATGCAGTCGCCAACGACAGT	TCAAACCATCAATTCCTGCAACCCAT

Table S3 List of primers used for cDNA isolation (*continued*)

Target	Nucleotide sequence (5'-3')			
	1st round PCR		Nucleotide sequence	
	Forward Primer	Reverse Primer	Forward Primer	Reverse Primer
AvSTS-09	TAGCGTTCTGCATCACTTTCATCCAAC	GGGTTGGACTGCGTCTCTACC	ATGCTCACTTCTCCCGGACATTTCTA	TCATGTGTCCTCCTCCCGC
AvSTS-10	CGCCATCCAAACTCCTTCGGA	GATAATGTGCCATTCGATGCTCCG	ATGTCTTCTCCCACTCACTTCGTC	CTAATCGTCCTCCAGTTTCTCCGACT
AvSTS-11	CTATCAATACTTCTTCCATTCGCACGGA	GGTAATGTGTTCGATGCTCCGCTC	ATGTCTTCTCCCACTCACTTCGTC	TCAATCTTCCTCCAGTTTCTCCAACT
AvSTS-12	AGCGCCCACTTTCCGACC	CACATACACTACACATACACCAAAGTCATACT	ATGGCCCTTTCTCAGTTTTCGACC	TCACGCCTTCTCCAACGGC
AvSTS-13	GCAGGTCCCACTTTGCGACC	ACCAACACATATTCAACACATACTCAACACATAAA	ATGGCCCTTTCTCAGCTTTGGAC	TCACGCTGAGTTCTCCAACGG
AvSTS-14	GACTCCAACACTTTATTCTATTCGAACGGA	GTGTGCCATTCAATGCTCCACTC	ATGTCTTCTCCCACTCACTTCGTC	TCAATCTTCCTCCAGCTTCTCCCTAT
LnSTS-01	CTCTTCACTGTCGCTCATCTCCA	TCGATAATACAGGAGAATCTATTTTGCAGCTT	ATGTCACCTTCGTCCGAGATATACTTCA	CTAAAATAATTCGTAGCGATGTAGTATAATTATGCTACGTA
LnSTS-02	GAAATACAGACCCTACCCGTGCC	GCAAGTAAGGTGAAATTTACTAATACATTGACTTTTTCAAAAAG	ATGGCGGACGTCATTGTGTTACC	CTATAGTACAAAATAGCATTGTATCCCTGCAGAAA
LnSTS-03	CATGGACCCTACCAATACAGATCCAAC	CGTGATATAAACCGTAAACGTGATTGTGGATG	ATGTCCACCCAATTTGTACTCCCC	CTATATGTCTCGAGGTAATTCAGGGTGC
LnSTS-04	TTTCACCTAGGGACAGTTCGCC	TCAATTTTTTGGAGCCGTTTGGGT	ATGGCTTTAATCACCAGTCTTAAATCCACAG	CTATTTAAACAGTTTATCTACCTTTGGAAGTAAAGTAACAG
LnSTS-05	TCCCCCTCTCGACTCCAACA	GGAAAGGGAAATTATGTAAATATATAAATGGACGAGAAGG	ATGTCCACCCGCACACCC	TTATTGGCATATGGCCACGGGA
LnSTS-06	CCGCCCAACTTCACTGGACC	TTTTATACTGATTATATAGCTTTATTACATAGCCGAAAGCTAC	ATGTCTTTCCACCTTCCGGATATCCA	TCAGACGTCTACAAGCTGGGG
LnSTS-07	GGTAGAACGTCGTTTTCGTCAGTCTAAAAT	GCACGAGGCTCTACTGCCTAC	ATGTCTTTCCACCTCCCGGATATC	TCAAACGGTTACGAGCGGGG
LnSTS-08	CGATCTCACCCTTCTTCTAAACACACAAG	GCACGAGGCTCTACTGCCTAC	ATGCTCACCTCAAGTCACCCTACTT	TCATTGTTCACGGGGTAGGAGTTTTA
LnSTS-09	ACACTCTCTTCCATCTCTCTTACTTACCA	AAGTGATTATGATGGAAAGATGGAACGGAAAAA	ATGAATCATACTACAACACAGATCCTCCTTCC	TCACTCAGTATCAGATTCTGAGTCTGTTTCT
LnSTS-10	TACCTTCGCCCTCCCTCACC	GAAGGAGATAGAACATAAACGAACATAATACTAAGAAGTT	ATGTCTGCCTCGACTTCAACAACA	TTAGACTCGGCGGGGGAGG
LnSTS-11	CGGGGGAAAATTATTCAATCATCTCAAAC	TAAAATAGGAATCCGCTGTCGACGAAAA	ATGGCCATCGACGTCCAGC	CTACTCAGTGATGGTTTTGGCCGG

Table S3 List of primers used for cDNA isolation (*continued*)

Target	Nucleotide sequence (5'-3')			
	1st round PCR		Nucleotide sequence	
	Forward Primer	Reverse Primer	Forward Primer	Reverse Primer
LnSTS-12	CCCCCGCCCCTTTCTTCATCTATATA	AGGTTGAGGCAGCTGGTTATGC	ATGTCTCTTCCGAGCAGGCC	CTACTCAGTGATGGTTTTGGCCGG
LnSTS-13	ACACCTATGTCAAGTTATCTGTACCCACA	AAAAAAAAAAAAATACATCAGTTATTGTTGGGAATATAAGGGC	ATGGACAGTCGACCATCTCTTTCCT	TTATACAGGCTTATACGATGGGAGGAGTT
LnSTS-14	GCCGTGGCCTTCCTCGAAC	GCGCCAATATGAATCTATGAAAATCATTGCATAATT	ATGGAAGATCACCTCACTTCTTCATCAG	CTACACCCGAGTAGGCTTAGAGGAA
LnSTS-15	ATATGTATTCCTATGATCATACTCCCCAATC	TAGTATTTTACGGCACATAGACCATTGGTTT	ATGGCAGGGAGATTTGAGTTGCCTGA	TCATCGAGCCCTCCTAACAGGGC
LnSTS-18	TTATAAATGAGCAACGACATTACAGAGTCAGTAAAA	TATCGCTTATAAGAGTACAGCTGGTAATACGA	ATGTCCCCTGCAAGAATTAAGACATTGC	TTAGAGCTCTAGTTCGTCCAAACGGTAT
LnSTS-19	TTTCAACACACTCCACCATTCCTATCG	ATATTATTAATGATCGAGTCAAAGAGTCCGGTAGA	ATGGCTTTGTACTCTTTGCGTTCATT	TCAAACTGCACCAGCGTCCC
LnSTS-20	TAGAACTCCTACATTTCCACAACCTTCTAAC	TACAACGTTATACAAATTCGATAAACCCCCATAAT	ATGGCCCCAGTTTCTCTTATTCGC	TCACCGTCTCGCAGTAACGT
LnSTS-21	CCAGAATTGTGAAGACTTTCCTTCACAAC	AAATTATAAAGTAACATTATTTGAATTTCCGAGGCCCAGAAA	ATGGATTTCTCCACACCGAACATTAACG	CTACAAAGATAATTCGCTTAATTTATACCGTTTCTGGT
LnSTS-23	CATACGTTTTCAATCGTTGGTTTCAGAGT	TTATCAGTACACGTTGTATCCGAGTCAAGTAT	ATGATGTCTGGAACTATTAGCACCATTATTGAG	TTAGATATTTAATTCTTCGAGTCGATAACGACCTT
LnSTS-24	CATCAAGTCCGACACGAAAAATGTCTT	CTGCCCACTCTAGCCTTTATATATGATTTACTATCTAT	ATGCCCTCATTTGACCTTATCGCG	TCATAAAGGAAACGGATATTCTAAAGTACTATAACGCG
LnSTS-25	ATATAAAGGGAGATGGGGTAGCTCCA	AAATCATAACACAGCCTTGTATGTGACAC	CCACTTTACCTCAGTATTTTCTGATCCAT	GACACTAAAACAACACTAAATGTCTACCCATAT
LnSTS-26	CAAACTTTTTTCTCAAGCTGATTTGAGAAAGCATATAAA	AGCATTTTGTACGTCTATTTCCTTGCATGATA	ATGGTTGTAGCTGCAGCACTGG	CTACAAATTTAGGTCGGCTAATTTGTAGCGTTT
LnSTS-27	TTTTCACCTTCGCCCTTCTTTGAAAC	GAACAATAATATTCATAGATTGTTATACACGCAATTCCAAATTTGTA	ATGAGTACAGTCACCCAACTTTTGAATAACTTC	TTACGCCTTATCGCCAAAACGAAG
PoSTS-01	TACTGAGCTCCACTGCACAC	TAACAGATCCGCTATGCGCA	ATGTCAGCCATCGCATCCTC	TTATGACCGTCGGGGAAGGA
PoSTS-02	CCTTAATTCCTCTTTAGCTTATTATCACC	GGTTTTTGAACACGTGTCGT	ATGTCGACGTTAATCTCTCCAGA	TCATGATGAAACCTTTGGAAGGA
PoSTS-03	GACTCTTCCATCGCTCTGCC	GGGGCTGCGTGTTTCCGAGG	TGGTCCCAGTCGCTTCCTT	TCACTTCGTGGCCTCGCCG
PoSTS-04	CCATTACTCTACCTCTATATCCCACA	GTCAGGTACAACCAATAGAAGAAGA	ATGGCCCCCACCGCCT	CTAGGCCACCGTGGGTTC

Table S3 List of primers used for cDNA isolation (*continued*)

Target	Nucleotide sequence (5'-3')			
	1st round PCR		Nucleotide sequence	
	Forward Primer	Reverse Primer	Forward Primer	Reverse Primer
PoSTS-05	CCACTGCTTCCCTCTCCACG	GAAGACGTGGACTCTCCGAT	ATGTCTTCCCAGCCAACTCA	CTATTCGTCGAAATCCTCGGGC
PoSTS-06	GCCTGCCTATATGTCGCTTTCTCG	GCCAGAGTCTTTGCTCGTGTGT	CCACCTCAGCAAGTTCCAGAGTC	GTCTAATAAGCGGTCAAGCAGATGATAAA
PoSTS-07	ATGTGTATCCGACTTGAAGACC	GGATCATTCGTTATTCACATCTCAA	ATGTCGACAACTTCCCTTCG	CTACCCACGACAGCTAGTGC
PoSTS-08	GCACCGAAGCCACCCCCATC	TGAGCCTAGTCCCTGGCTGC	ATGCCTCCCAGTGGAATAGCA	TCAAATAATTGATGCCTTGGTCTGTAC
PoSTS-09	GCTCGATTATACTTGAAGACGCC	CTTTGTCTACTTCTAATTGAGGGTAC	ATGAACAGAGGTGCAAGCTACC	CTACGATGAAACTACGGGTAGGAAT
PoSTS-10	GTTTCTCTACTATCTCGCCGTC	CATCTGAAGTTGAATGTTCCGT	ATGGTCTTTGTCCAATATCGCCT	TCACAATGGGGCATAAAATTCGAT
PoSTS-11	GAGACAGCACTCATCGATCTAC	CGGTCTATCAATGATCGAGCGC	ATGGCCTCGCATTACCGTTT	TCATATTTTCACCACCTCATTATTCCTC
PoSTS-12	CACCAGATACCCTCCAAGCA	AATCATAGATGCTGATGTTCTGATATG	ATGACATCTGCAACTGCGAC	TCACAATAGCTCTTCTAGTCTATACCT
PoSTS-13	AATCTGACCCAAACCTCGATA	ACAACCTTAGTACGCCTCTC	ATGAAGGTACTCAATACAACGCCC	TCAGCAGAAAAGGTCGTTTAGC
PoSTS-14	TCGTACGCAAGTAGAGACCTTAAC	ACATGCACCGCCAACTCTG	ATGAGTATGAATCAAGAGATTCGCC	TTACGATGTCAGGTTCTGAGTCAC
PoSTS-16	ACACCTCCAGTTGTCCAGAC	GCAATATCACATTGTTGTGACTCTG	ATGCCTGTCAATATCCGTGAC	CTAATTACACAAATTACGTTGACAAACTT TACA
PoSTS-17	CGATGCACTTACTATCCCTCTAG	CCACAATGTACAGAACGTTTACG	ATGCCATCCGACGACGAGAG	CTATACGCTTCGACCTGCCA
TvSTS-01	CTCCTCTCCTTAACCGAGCC	ACGTGCCGGACAGACAGCAG	ATGGCCGTCATCGCCGC	CTACTTGCGCCGCGGGAGAA
TvSTS-02	ACGTGCCGGACAGACAGCAG	GCAGCGAGAGCCGAGAGAGG	ATGCTTCAAGCAAGCTCT	TCAACGAGTGAAGATGCCGGC
TvSTS-03	TTCTGTAATTCCCGACCGCC	GGGATCTGGCTGGATGAAAG	ATGCCGCGGGCTCCTCG	TCAACTTGACAGACCGGTCC
TvSTS-04	GCAGCGAGAGCCGAGAGAGG	CGCGCGCAGGGCGC	ATGCCCGTCATCCTCGCTTT	TCATGCGCGGACGCGCT
TvSTS-05	CCAGGACGATCGCTCTCGAT	GCGCTGTGGGCCCGCT	ATGTCTTCCCCGTCTTCGTT	CTAGTTGTCCTCCTCGTCGG

Table S3 List of primers used for cDNA isolation (*continued*)

Target	Nucleotide sequence (5'-3')			
	1st round PCR		Nucleotide sequence	
	Forward Primer	Reverse Primer	Forward Primer	Reverse Primer
TvSTS-06	GGGATCTGGCTGGATGAAAG	GCGGGCGTGCGCACC	ATGTCTGCCCAACAGTTCACC	TCAGTTTTCGTCCGCGGGGC
TvSTS-07	CCCCGCCGACGCCCAG	TAAACGGACGATCGCCCTCT	ATGTCTCGCTCTCTTCGTCT	TTAATCTCTAGTCTTGAGCTCGATGAC
TvSTS-08	CGCGCGCAGGGCGC	TCAAATCACGTCTCCTAACCAT	ATGGCTCAGAGATATCTCCACA	CTACGCTGGGGTCGGTTGG
TvSTS-09	CTTTCACCCGCACTAGCACA	CCCGTTGTTACCTACGCTGA	ATGCCTCAGCAGTATATCCACA	TCATGAGGTCGGCTGGGCAA
TvSTS-10	GCGCTGTGGGCCCGCT	TAGCTAACTGGATTCATGGTCAT	ATGGCGACCTGGCCGTGG	CTATGCTGGGGTCGGCTG
TvSTS-11	AAACCACCTCCACCAACGTC	GCGTCTTCGCACGCATGATC	ATGACCCACCTTTTTCACATTCC	TCACGCGACGCGTGGCTG
TvSTS-12	GCGGGCGTGCGCACC	AAGAGGAATAGCGTAGCGGT	ATGAGCGTCGAGCAGCTCCG	TCACGGGAGAGCGAGACGAA
TvSTS-13	CACCCCGCACTACACTACCA	GAGGGCGTCAGCATCATTAA	ATGGGGAGCATCCGCATCCC	TCACGCGTTCATGAACACCC
TvSTS-14	TAAACGGACGATCGCCCTCT	ACCTGCACCGTCGCCCTGT	ATGCCCTACATCCAAGATGC	TCAATACTCCATCTCCAAATCCTC
TvSTS-15	TCAAAGCATTTCCTCCGCTC	ATCGCAGGAACCCTTCTCCT	ATGATGCAAAACGCCAACAC	TCAAGCGCTGAACCCCA
TvSTS-16	TCAAATCACGTCTCCTAACCAT	ACGTTCAACGCCACCATCTC	ATGGTCGCAGAAGTACTCCC	TCATTCCGTCAACCCCAGG
TvSTS-17	CTTCAGAAGGCCCGCTCCGC	ACCTTCGCGAGACCTCGCTA	ATGAGCCTCCCTAAGATACTCGT	TCACGCTGTGAATCCAAGTT
TvSTS-18	CCCGTTGTTACCTACGCTGA	GGTATCGCGAATGGACACGT	ATGCAGACTGCGGTGATCAC	TCAGGCAAGTCCAAGATCAGCA
TvSTS-19	TCGTACAGCCGCCTCAGACC	CCAGATTGTCTCCAAGTCATAGAC	ATGCCGTCCGCCGTCAGT	TCAGAACTCTATCTGCAGCTCG

Table S4 List of primers used for construction of pGYRG-based expression plasmids

Target	Nucleotide sequence (5'-3')	
	Forward Primer	Reverse Primer
AbSTS-01	TGGAAAAAATGACCGCCGCTTTTGCAGTCGCTTCC	TTCTCGAGGGACTAGTTACAGATACCCGTAGTG
AbSTS-05	TGGAAAAAATGACCGCCCCTCGCCCTCAACAATTC	TTCTCGAGGGACTAGTTATACCAACAACTCATT
AbSTS-07	TGGAAAAAATGACCGCCAGAGCCATTGAACAACTCTCTAACCA	TTCTCGAGGGACTAGCTAATGAATGCCTAGCTCCATGAGGC
AbSTS-09	TGGAAAAAATGACCGCCGGAGCCGTTGGACAACTC	TTCTCGAGGGACTAGCTAGTTAAAGCCTAGCTCC
AvSTS-01	TGGAAAAAATGACCGCCCAGTCGCCAACGCAAGCC	TTCTCGAGGGACTAGTCAAACCATCAACTCCTG
AvSTS-03	TGGAAAAAATGACCGCCCCTGAGAAGTTCTACATC	TTCTCGAGGGACTAGCTACAACATGGAACCGTC
AvSTS-04	TGGAAAAAATGACCGCCCAATCACCAACGCAAGAT	TTCTCGAGGGACTAGTCAGACCATCAACTCCTG
AvSTS-06	TGGAAAAAATGACCGCCTCCACCCAAATCCAATTC	TTCTCGAGGGACTAGTTACAGGTACATCCAACG
AvSTS-07	TGGAAAAAATGACCGCCCAGTCTCAAATCCCTCTT	TTCTCGAGGGACTAGTCAAACCACCACCGAATT
AvSTS-09	TGGAAAAAATGACCGCCCTCACTTCTCCCGGACAT	TTCTCGAGGGACTAGTCATGTGTCCTCCTCCCG
AvSTS-11	TGGAAAAAATGACCGCCTCTTCTCCCACTCACTTC	TTCTCGAGGGACTAGTCAATCTTCCTCCAGTTT
AvSTS-12	TGGAAAAAATGACCGCCGCCCTTTCTCAGTTTTCG	TTCTCGAGGGACTAGTCACGCCTTCTCCAACGG
AvSTS-13	TGGAAAAAATGACCGCCGCCCTTTCTCAGCTTTGG	TTCTCGAGGGACTAGTCACGCTGAGTTCTCCAA
LnSTS-01	TGGAAAAAATGACCGCCTCACCTTCGTCCGAGATA	TTCTCGAGGGACTAGCTAAAATAATTCGTAGCG
LnSTS-02	TGGAAAAAATGACCGCCGCGGACGTCATTGTGTTA	TTCTCGAGGGACTAGCTATAGTACAAAATAGCA
LnSTS-03	TGGAAAAAATGACCGCCTCCACCCAATTTGTACTC	TTCTCGAGGGACTAGCTATATGTCTCGAGGTAA
LnSTS-04	TGGAAAAAATGACCGCCGCTTTAATCACCAGTCTT	TTCTCGAGGGACTAGCTATTTAAACAGTTTATC
LnSTS-07	TGGAAAAAATGACCGCCTCTTTCCACCTCCCGGAT	TTCTCGAGGGACTAGTCAAACGGTTACGAGCGG
LnSTS-08	TGGAAAAAATGACCGCCCTCACCTCAAGTCACCCT	TTCTCGAGGGACTAGTCATTGTTCACGGGGTAG
LnSTS-09	TGGAAAAAATGACCGCCAATCATACTACAACACAG	TTCTCGAGGGACTAGGAATCTGATACTGAGTGA
LnSTS-10	TGGAAAAAATGACCGCCTCTGCCTCGACTTCAACA	TTCTCGAGGGACTAGTTAGACTCGGCGGGGGAG
LnSTS-18	TGGAAAAAATGACCGCCTCCCCTGCAAGAATTAAG	TTCTCGAGGGACTAGTTAGAGCTCTAGTTCGTC
LnSTS-19	TGGAAAAAATGACCGCCGCTTTGTACTCTTTGCGT	TTCTCGAGGGACTAGTCACCCCCTCGCAGTAAC

Table S4 List of primers used for construction of pGYRG-based expression plasmids (*continued*)

Target	Nucleotide sequence (5'-3')	
	Forward Primer	Reverse Primer
LnSTS-20	TGGAAAAAATGACCGCCGCCCCAGTTTCTCTTATT	TTCTCGAGGGACTAGCTACAAAGATAATTCGCT
LnSTS-21	TGGAAAAAATGACCGCCGATTTCTCCACACCGAAC	TTCTCGAGGGACTAGCTAAAGTCCCCGGTCTTGG
LnSTS-25	TGGAAAAAATGACCGCCTCTCAAGACACTAGCAAA	TTCTCGAGGGACTAGCTAAATGTCTACCCATAT
LnSTS-27	TGGAAAAAATGACCGCCAGTACCGTCACCCAACTT	TTCTCGAGGGACTAGTTACGCCTTATCGCCAAA
PoSTS-01	TGGAAAAAATGACCGCCTCAGCCATCGCATCCTCC	TTCTCGAGGGACTAGTTATGACCGTCGGGGGAG
PoSTS-02	TGGAAAAAATGACCGCCTCGACGTTAATCTCTCCA	TTCTCGAGGGACTAGTCATGATGGAACTTTTGG
PoSTS-03	TGGAAAAAATGACCGCCAGTGAAGCACCGGAGCGG	TTCTCGAGGGACTAGTACCAGGGTCAGCGAAGG
PoSTS-05	TGGAAAAAATGACCGCCTCTTCCCAGCCAACTCAA	TTCTCGAGGGACTAGCTATTCGTCGAAATCCTC
PoSTS-06	TGGAAAAAATGACCGCCACCTCGACTACGAAACACCCC	TTCTCGAGGGACTAGTCAAAAGGACGACGGAAAAGAAGAATG
PoSTS-09	TGGAAAAAATGACCGCCAACAGAGGTGCAAGCTAC	TTCTCGAGGGACTAGCTACGATGAAACTACGGG
PoSTS-11	TGGAAAAAATGACCGCCGCCTCGCATTACCGTTTACCCAA	TTCTCGAGGGACTAGTCATATTTTCACCACCTCATTATTCCTCGC
PoSTS-16	TGGAAAAAATGACCGCCCCTGTCAATATCCGTGAC	TTCTCGAGGGACTAGCTAATTACACAAATTACG
TvSTS-01	TGGAAAAAATGACCGCCGCCGTCATCGCCGCCTCA	TTCTCGAGGGACTAGCTACTTGCGCCGCGGGAG
TvSTS-04	TGGAAAAAATGACCGCCCCCGTCATCCTCGCTTT	TTCTCGAGGGACTAGTCATGCGCGGACGCGCTG
TvSTS-05	TGGAAAAAATGACCGCCTCTTCCCCGTCTTCGTTT	TTCTCGAGGGACTAGCTAGTTGTCCTCCTCGTC
TvSTS-06	TGGAAAAAATGACCGCCTCTGCCCAACAGTTCACC	TTCTCGAGGGACTAGTCAGTTTTCGTCCGCGGG
TvSTS-07	TGGAAAAAATGACCGCCTCTCGCTCTCTTCGTCTT	TTCTCGAGGGACTAGTTAATCTCTAGTCTTGAG
TvSTS-09	TGGAAAAAATGACCGCCCCTCAGCAGTATATCCAC	TTCTCGAGGGACTAGTCATGAGGTCGGCTGGGC
TvSTS-10	TGGAAAAAATGACCGCCGCGACCTGGCCATGGCCC	TTCTCGAGGGACTAGCTACGCTGGGGTCGGCTG
TvSTS-12	TGGAAAAAATGACCGCCAGCGTCGAGCAGCTCCGC	TTCTCGAGGGACTAGTCACGGGAGAGCGAGACG
TvSTS-13	TGGAAAAAATGACCGCCGGGAGCATCCGCATCCCG	TTCTCGAGGGACTAGTCACGCGTTCATGAACAC
TvSTS-14	TGGAAAAAATGACCGCCCCCTACATTCAAGATGCA	TTCTCGAGGGACTAGTCAATACTCCATCTCCAA
TvSTS-16	TGGAAAAAATGACCGCCGTCGCAGAAGTACTCCCT	TTCTCGAGGGACTAGTCATTCCGTCAACCCCAG

Table S5 List of primers used for construction of pALLF-based expression plasmids

Name of Primer	Nucleotide sequence (5'-3')	Reaction step*
tdh3pF	GAATAAAAAACACGCTTTTTCAGTTCGAGTTT	Step-1
tdh3tR	ATCCTGGCGGAAAAAATTCATTTGTAAACT	Step-1
tdh3_invF	GTGAATTTACTTTAAATCTTGCATTTAAATAAATTTTC	Step-2
tdh3_invR	TTTGTTTGTTTATGTGTGTTTATTCGAAACTAAGTTC	Step-2
thmg1_recF	ACATAAACAAACAAAATGGCAGACCAATTGGTGAAAACTGAAGT	Step-3
thmg1_recR	TTAAAGTAAATTCACTTAGGATTTAATGCAGGTGACGGACC	Step-3
tdh3t_infF_Y2	GGCGATGGCCCACTAATCCTGGCGGAAAAAATTCATTTG	Step-5
tdh3p_infR_Y2	GGGTGATGGTTCACGGAATAAAAAACACGCTTTTTCAGTTCG	Step-5
f1ori_invF	CGTGAACCATCACCCTAATCAAG	Step-6
f1ori_invR	TAGTGGGCCATCGCCCTGA	Step-6
pBlue_invF	CAGCTTTTGTTCCCTTTAGTGAGGG	Step-8
pBlue_invR	CAATTCGCCCTATAGTGAGTCG	Step-8
tpi1p_infF	CTATAGGGCGAATTGGATCTACGTATGGTCATTTCTTCT	Step-9
tpi1p_infR	TTTTTCTGAAGCCATTTTTAGTTTATGTATGTGTTT	Step-9
erg20F	ATGGCTTCAGAAAAAGAAATTAGGAGAGAGAG	Step-9
erg20_infR	AGGGAACAAAAGCTGCTATTTGCTTCTCTTGTAAACTTTGTTC	Step-9
tpi1p_infF_Y2	GGGATGATCCACTAGGATCTACGTATGGTCATTTCTTCTTCAG	Step-11
egr20_infR_Y2	TAGATGCATGCTCGACTATTTGCTTCTCTTGTAAACTTTGTTC	Step-11
gapdh_NaeI_F	TTGACGGGGAAAGCCGAAATGCAGTTCTCCATGCTGGCAC	Step-14
gapdh_NaeI_R	CTCGCCACGTTCGCCCTGCAGAAAGCCCTAAGATGCTCCT	Step-14

Nucleotide sequences of isolated STSs

```
>AbSTS_01
ATGGCTTTTGCAGTCGCTTCCTCGTCCCCCAAGTCTTTTATTCTTCCTGATCTCGTGTCCCATTGTGACTTCAAACTACGAATCAGCCGACACA
GGAAGCAGGTCACCGTTGAGACGAAGAAATGGCTTTTTCAAGGGGATAGCCTTGACGCAAATAAACGCAAGGCCTTCCATGGGCTCAATGCTGG
TTTACTGACCGCAATGACCTATCCCAATGCTGCTTGCCCCCAGTTGAGAGTTTGTAATGATTTCCTCACTTTTTTATTCCATCTCGACAACCTT
TCCGATGATATGGACAATCGAGGAACTCACAACATTGCCGATGTCGTGTTGAACTCCCTCTATTTCCCTTACTCTTACCCTTCCACCGCCCGCA
TTGGGAGGATGACCAAACATCTATACAAACGAGTGATCCCGACCTCGTCTCCTGGAACGCAGCAACGTTTCATTGAAACAATGGATTTCTTTTT
CCAGAGCGTAACCCAACAAGCTCTGGATCGCGCTAATGGTGTAATACCCGATCTTGACTCATATATCGCTCTACGTCGGGATACTTCCGGATGC
AAACCGTGCTGGGCTTTAATTGAGTATGCAAACAATCTTAAAATTCCCGATGAAGTGATGGATCATCCCACCATCCTCGCCCTCGGTGAGGCTG
CCAACGACCTTGTCACATGGTCTAATGATATCTTCTCCTATAACGTAGAACAGTCGAAGGGCGACACGCACAATATGATTCCGGTCGTTATGTA
TCAGAAGGATCTCAGCCTCCAGGAAGCCGTGGACTTTGTCGGTCAAATGTGCAAGTCGAGTATCGACCGTTTCAAGGCGGAGAGGGATAATCTT
CCTTCCTGGGGACCACACATCGACCGGCAAGTGAATGTATACGTTCAAGGCCTGGCCGATTGGATTGTTGGTTCGTTGCATTGGTCATTCGAAT
CAACAAGATATTTTGGGTCAATTGGACGCAAGATCAAGTCAACCCGTGTTGTTGAACTCCTTCCTCTGCGCCAAGAAGCTAGGGCAAGGGCGGC
AACTGTCACTTTGCGTCCCACCCTCTCGCCAATCACGGTCCCCCCTCGGGCTCCAAGTCCCGCTACCCCAACGGAATCGGAACCGGTCACACCC
TCCAGCAGCGTTCACAGCATCCCTGTACCCAAATCAGCCGATTCAGAGGAGTCTATTCCTCACTCACCTTGGTCTGTCCAACGTTCTCAAGACG
TCATTGTCAGTCCGATTCCCAACTCGCCCAAACCAACTCTTGGGCGTATTCAAACCGAGTCGCTTTTCGTGTCAGAGACTCAAGAGCAGACACC
GGAGAGTGGTGTGCTCGTACAATATCCAGAAATTCTTTCTCCAGCAATGGCCAATCCTATGGTGAACCAGAGAATCGTCAGGAACGAACCTCTC
GGCCTCTACTCACTTACCATTTGGCTTTTCTACACTTTGATCTATGCGCCTACTATGACGCTTCTTCGTGTTTTCCTCAATCAAGATGTGCATC
CACATCCTCTCTGTCACTACGGGTATCTGTAA
//
>AbSTS_05
ATGCCTCGCCCTCAACAATTCATTCTCCCAGACTTACTCTCTAGTTGTCCTCTCGAGGATGGTTTGAATCCCCACTACCGAGAAGCAGCCGCAG
AGTCTCGTGCCTGGATCAACTCTTTCAATATTTTCTCCAACCGAAAACGTGCCGACTTTATCCAAGGTCTTAACGAGCTCCTTTGCTCTCACGT
ATATTGCTACGCTGGATACGAAGAATTCAGAACTACATGTGACTTTGTTAACGTGTTGTTCGTCGTTGATGAGATCAGCGATGAGCAAAGTGGC
AAAGATGCTAGAGCGACCGGCTTGAGCTATGCTGAATCTATGAGAAATGCCGACTGGGACGACAATTCCGTAGTTGCTAAGATCACGAAAGAGT
TTCGTGCGCGTCTTATCCGACGGGCTGGACATAATAATTTTCGTCGCTTTGTCGCGTCTTCTGATGCGTACACACGATGTGTAGGCAGAGAAGC
CGAGCTCCGAGAGGCAGGAGAGGTACTATCGTTGGAGGAGTACATGCCCCTTCGACGGAATAATAGCGCCGTCTTGTTATGCTTCGATTTGGTG
GAGTATATCCTCGGGGTTGACCTCCCTGAGAGTATATATCAAAACGCTACCTTCTTGAAGGCATATTGGGCAGCGTGTGACCACGTCTGCTGGT
GTAACGATGTCTACTCATATAACGTGGAGCAATCAAAGGGACACACCGGCAACAACGTCGTTACCGTTCTCATGAATGACCGTCAAATCGGACT
CCAGGAGGCATGCGACTACATCGGTGACCGTTGCCGGCAATTTATGAATGATTATCTTGCCGCGCGGGATGAGCTCCGAGCGACTGTTGGCGGT
GACGCTTCACGGTTCATCGATGCTCTAGGTTATTGGATAATTGGTAACATGGAGTGGAGCTTCGAATCTCCTCGTTACTTTGGCCATGAACATG
ACGAGATCAAACGCACCCTGACTCTGACTTTGAAACCCAGCGAGGTGCCCGAGGAGGTAGACTCAGATTCAGATTGCGAGTGA
//
>AbSTS_07
ATGAGAGCCATTGAACAACTCTCTAACCAACCTCTTTCGCACAAGGCTCTCGAAGATGGCGTTCGCGACGTCATCAGCGACTTTCTCTTGAAAA
CCAACCAGACACCCAAAGCCCCGGAGCAACCTGACGCCCTCGTCTTTGAGCGGGAATGTTGGGCTGAGGCTGACCGTCGCGGCTACGACATGCA
CTATCTTTCGAAGCATCTCCCTGTCGGGATACTCGTCTCCAATGCTGCATATTTCTACCAGTCCTTTGAACTCAGGCTCTACATAGCATATTAT
ACCGGCCTCCTGCTTTGCGTCGATGACAATTTCGACATTCAATCCGATGGTATTGCCCGCTTTATGGAACGATACCAGAGGGGCGAATGTCATC
CCACTGACGTCCTCAACAACCTTGCGGACTTACTGGCCGAGACCTCCACCTTCTTTGACCCTGTCGCCACAAATCTGATAATGTGTGCTACATT
CTCATTCATGAACGCTATGGTCATCGAAAACATCACCGCTGGAACTAAGATTCCCTCACAAGCGAAACGGTACCCCGATTATTTGCGTCGGTTC
TCTGGAATATCCAAGGCCTATGCTACTTTCATTTTTCGGCCGTCAGTCAGTGCCGCTCAGTATATTCATGCCCTCCCAGAACTTGAGGATATCA
TCAACTACGTCAATGATATAACGTCATTCTATAAAGAAGAGCTCGCTGGAGAGGATGAGAACACTGTTTCGCTATTAGCAAAATTAAACAACCG
GTCGAAACTCGACCAGCTGCGTGTACTTTCCGACAGCGTCGCCGAATCTCACCGCCGAACCTTAGCAATTCTCAAAGGTAGAGACGATGCCGAG
GGTGATTATTTACTCTTCTGGTCTGGTTATATACCTTTCCATGTTTCGGCAAAGAGATACCGCCTCATGGAGCTAGGCATTCATTAG
//
>AbSTS_09
ATGGGAGCCGTTGGACAACTCTCTAACCAACCTCTTTCACGCGAGGCTCTCGAAGATGGCGTTCGTGACGTCATTAGCGACTTCCTCTTGAAAA
CCAACCAGACCCCCAAAGCCCCGGAGCAACCTGACGCCCCCGTTTTTGAGCGGGAATGTTGGGCCGAGGCTGATCGTCGCGGCTACGACATGCA
CTATCTTTCGAAGCATCTCCCGGTCGGGATCCTCATCTCCAATGCTACATTTTTCTATCATTCCTTTGAACTCAAGCTCTATGTGGCGTATTAT
AGCGGCATCCTGCTTTGCGTCGACGATAATTACGACACTCAATCCCGGGTCGATGGCATCGCCCGCTTTATGGAACGATACCAGAGGGGTGAAC
GTCATCCCACTGACATCCTCAACAATCTCGCGGACTTACTAGCCGAAACCGCCACTTTTTTTGACCCTGTCGCCACAAATCTGATAATGTGTGC
TACGTTCTCATTTATGAACGCCATGGTCATCGAAAACATCACCGCTGGAATGAAGATTCCATCACAAGCGAAACGGTACCCCGATTGTTTGCGT
CAGTTCACTGGAATTTCCAAGGCCTATGCTGCTTTTATTTTTCGGCCATCAGTCAGCGCCGCTCAATACATCCACGCCCTCCCAGAACTTGAGG
ATATCATCAACTACGTCAATGATATAGCATCATTCTATAAAGAAGAGCTCGCCGGAGAGGATGAGAACGCTGTTTCGTTATTAGCAAGATTAAA
CGACCGGTCGAAACTCGACCAGTTACGTGTACTTTCCGACAGCGTCGCCGAATCTCACCGCAGAATCTTAGCAATTCTCAAAGGTAGAGATGAT
GCCGAGCGCGACTACTTGCTTTTCTGGTCTGGTTATATACCTTTCCATCTTTCAACGACGAGATACCGCCTCATGGAGCTAGGCTTTAACTAG
//
>AvSTS_01
ATGCAGTCGCCAACGCAAGCCTCTTATCGTTTGCCTGAGACACTTGCGGGATGGCCGTGGCCACGCATGATTAACCCACATTACGAGGAGGTCA
AGGCTGAGAGTCGAGCATGGTTTCACAGCTTCCGCGCATTCGGTCCCAAGTCTCAAGATGCCTTCGACAAATGCGACTTTTGGCTGCTTGCGTC
TTTGTCGTATCCATTCACTGACAAAGCGAGGCTTCGTACGGGCTGTGATCTGATGATGCTGTTCTTCGTCTTTGACGAGTACACCGACCTTTCC
CACGGCAACGACGTCCGCGTTTATGCGGACATGGTCATGGACGCCCTTCGTAACCCCCACAAGCCCCGTCCTGCGGGGGAGATCCTCCTCGGCG
```

AGGTGGCCAGACAGTTTTGGGAGCTTGCTGTCAAGACGACGACCCCCACAGCACAGAAGCGCTTTGTGGATGCTTTCACGAGGTACACGGACGC
CGTGGTTGCGGAAGCGCGGGACCGAGATGAGACACATGTCCGGAGCATCGATGAATACTTCAACATTCGTCGGTACACCATCGGCGCCGAGCCT
TCGTACGTTCCGATGGAGCTCGCGATGGACATTCCGGACGAAGTGTTCTTCCACCCAACGGTCGTGAAGCTGACGCAGCTTGTCACCGATGTCA
TCCTTCTCGACAATGACCTATGCTCATACAACAAGGAGCAAGCGAACGGCGAGGAGCTTCACAATATCCTCACGATCGTGATGGCAGAGCTCAA
GGTCGACCTCAACGGCGCACTAGACTGGCTGGAACGGCGCCACGCGGAACTGAACGAGGCAATCATCGAGACGTGGAACAGTCTGCCCGTGTGG
GACGAAGACATCCGCGACGACGTGGACGAGTACCTCCTCGGTGTTGTCGGGTGGGTGCGCTCGAACGACTCGTGGAACTTCGAGAGCCAGCGGT
ACTTCGGGACAGACGGCCTCGAGATTCAGAAGCATAGGATGGTCACCATGAGACCAAGAAAGGCGGGATTGGGGTGCCCAGTAGAGGGTGACCC
GCTGCAGGAGTTGATGGTTTGA
//
>AvSTS_03
ATGCCTGAGAAGTTCTACATCCCTAACTGCCTCGAGAACTGGAAGTGGCCTCGCGCACTGAACCCATACTACGAGGAGGTGAAGGCCGAGTCCG
CAGCATGGGCAAGGAGCTTCGGCGCCTTCAGCCCGAAGGCCCAACATGCCTACGACCGCTGCGACTTCAACAAGCTTGCCTGCCTGGCGTACCC
GCTGCTCGACAAGGCCGGCGCGCGTATAGGATGCGACCTCATGAACATGTTCTTCGTCTACGACGAGTACTCCGATGTCGCGCCGGCGGATGAG
GTCCAAGTGATGGCCGACATCATCATGGACGCCCTCCGCAACCCGCACAAGCCCCGGCCAGAGGGCGAATGGGTCGGCGGCGAGGTGACCCGGC
AGTTCTGGGAGCTGGCAATCAAGACAGCCAGCCCGCAGTCGCAGAAGCGCTTCATCAAGACGTTCGGGACATACACCCAGTCGGTCGTCCAGCA
GGCCGCAGACCGCGACCACCACTACGTCCGCACTGTCCAGGAGTACCTCGAGGTGCGCCGGGACACGATTGGCGCCAAGCCGTCGTTTGCCATC
CTGGAAGTGGGCATGGATATCCCCGACGAGGCCATCGAGCACCCTATCATTCAAGAGTTGACGATCCTGTCCATCGACATGATCTTGCTTGGCA
ACGACATCGCATCGTACAACCTTGAGCAAGCCCGGGGCGATGACAACCACAACATCGTCACGATCGTCATGCACCAGGAGAAGACGGACATCAA
GGGGGCGATGGACTGGGTCGTCCAGTACCACAAGACACTCGAGGACCGGTTCATGGAACTGTATGCGCAGGTGCCGTCCTTGGACTTCGGCGAG
CGTGTGAACAAGGAGCTCGCCGTCTACGTTGATGGCCTGGGTAACTGGGTGCGGGCCAGCGACCAGTGGGGCTTCGAGAGCGAGCGGTACTTCG
GAAAGAAGGCCCCGGAGATCCAAAAGACGCGGTGGGTAACCCTGATGCCGAAGGAGCGCACAGACGAGATTGGCCCCCAGATCGTGGACGGTTC
CATGTTGTAG
//
>AvSTS_04
ATGCAATCACCAACGCAAGATTCTTATCGTTTGCCTGAGACACTTGCGGGATGGCCGTGGCCACGCATGATTAACCCACATTACGAGGAGGTCA
AGGCTGAGAGTCGAGCATGGTTTCACAGCTTCCGCGCATTCGGTCCCAAGTCTCAAGATGCCTTCGACAAATGCGACTTTTGGCTGCTTGCGTC
TTTGTCGTATCCATTCACTGACAAAGCGAGGCTTCGTACGGGCTGTGATCTGATGATGCTGTTCTTCGTCTTTGACGAGTACACCGACCTGTCC
CACGGCAACGACGTCCGCGTTTATGCGGACATGGTCATGGACGCCCTTCGTAACCCCCACAAGCCCCGTCCTGCGGGGGAGATCCTCCTCGGCG
AGGTGGCCAGGCAGTTTTGGGAGCTTGCTGTCAAGACGACGACCCCCACAGCACAGAAGCGGTTCGTGGATGCTTTCACGAGGTACACGGACGC
CGTGGTTGCGGAAGCGCGGGACCGAGATGAGACACATGTCCGGAGCATCGATGAATACTTCAACATTCGTCGGTACACCATCGGCGCCGAGCCT
TCGTACGTTCCGATGGAGCTCGCGATGGACATTCCGGACGAAGTGTTCTTCCACCCAACGGTCGTGAAGCTGACGCAGCTTGTCACCGATGTCA
TCCTTCTCGACAATGACCTATGCTCATACAACAGGGAGCAAGCGAACGGCGAGGAGCTTCACAATATCCTCACGATCGTGATGGCAGAGCTCAA
GGTCGACCTCAACGGCGCACTAGACTGGCTGGAACGGCGCCACGCGGAACTGAACGAGGCAATCATCGAGACGTGGAACAGTCTGCCCGTGTGG
GACGAAGACATCCGCGACGACGTGGACGAGTACCTCCTCGGTGTTGTCGGGTGGGTGCGCTCGAACGACTCGTGGAACTTCGAGAGCCAGCGGT
ACTTCGGGACAGACGGCCTCGAGATTCAGAAGCATAGGATGGTCACCATGAGACCAAGAAAGGCGGGATTGGGGTGCCCAGTAGAGGGTGACCT
GCTGCAGGAGTTGATGGTCTGA
//
>AvSTS_06
ATGTCCACCCAAATCCAATTCCGCCTCCCGGACCCGGTGGAAATATGCCATTGGCCGCTGCCCCGCATGCTCAACGCCCACTACGCCGAAGTAA
AGGCCGAATGTGTTGCGTGGATTCACAGCTTCAACGCGCTGAGCCCAAAGGCGCAGAAGGCTTTTGACAAGTGCGACTTCTCCTTATTAGCATC
CCTCATATACCCGTCGCTGGATCGACAACACCTGCGCACAGGATGTGACCTCATGATGTTGTTTTTCGTGTTCGACGAATTCACGGACAAGGAG
GACGGCAAAGGCGTCCGGAAGTATGTGGACATTGTTGTAGATGCGATAGAACACCCCGACCGTCCGCGGCCTGCCGGCGAGCATGTTCTGGGCG
AAATCACTCGACAATTCTGGGAGCGTGCAATACAGACCGCAAGTGCCTCTTCTCAACGGCATTTTGTCAAAACGTTCAAGGAATACGCGGAAGC
TGTCATTGAGGAGGCGTCCGACCGCGTCAGCGACCGTGTTCGCAGTATCGATGATTATCTCGCACTCCGCCGCCTGACTGCTGGTCCATACCCC
GGATTCTTCCCATGCGAAATCCGCGTGGATCTTCCGGACGATGTCTTCTATCACCACTCTGTCGCCAACCTCACACGGCTTGTCGCCGAGTCGG
TCGTGGTCACAAACGATACCTACTCCTACAACATCGAGCAAGCCGCTGGGCACCAAGGCCACAATATCGTCACTGTCGTCATGCGCGAGAAACA
TCTCTCCCTCCACCAGGCCCTCGAGTGGGTGGGCAATTATCACGCCGGCATCCTCACGGAGTTTCTCGAGTCCCGAAAGAAGCTGCCATCCTTC
GGCGAAGAGCTTGACGCACAGGTCGCAGACTATGTCGAGGGCCTAGCGCATGGGGTACGGGGGCTGGACAACTGGTGCTTCGAAAGCGGGAGGT
ATTTCGGCAGCAAGGGTTTGGAGGTTCAGAAGCATCGGACCGTTGGCCTGCTGCCAAAGGTCGTGCAGCACGACGTCGCCACTCCGATGATGGC
GCTGCCCGTATCGGATCTCGATAAGATGGAGGTGGGAGAGTCCGACCTCGAGAAGCATCAGTCGTCGGGGTGGAGATACTTCGACTGCTTCAGT
TGCCTTTCGGGCCGTTGGATGTACCTGTAA
//
>AvSTS_07
ATGCAGTCTCAAATCCCTCTTACACACTTCCGTCTTCCTGATCCGGCCGCCAACTGGCCATGGCCACGAACTCTCCATGTCAATTATGAAGAGA
TCAAAGCTGAGGCCGACGCTTGGCTTCATAGCTTTAATGCCCTGAGCTCCAAGGCTCAGAGGGCTTTTGACAAGTGTGACTTCTCACTACTCGG
ATGCCTGCTCTATCCTCACCTCGACAAAGAGCGCGCCCGCACTGGCTGCGAACTGATGATCCTCTTCTTCATCTTCGACGAGTTCACGGACCAA
GAAGACGGGCCCGGCGTCCGCAGATACGTCGACATCGTCCTCGACGCGCTCCGCAACCCACATGTGCCACGTCCGGTCGGCGAGCACGTCCTCG
GGGAGATAACGCGATCATTCTGGGAGCGCGCGATCAAGACTGCGACGGCAGCGTCGCAGCGGCACTTTATCCAGACGTTCTCCGAGTACGCAGA
GGCGGTGATCCTCGAGGCGGCCGACCGCGCGAGCGAGCGCGTGCGCGGGATCGAGGACTACCTCGCGCTGCGCCGTCTCACCGCGGGACCGTAC
CCCGGCTTCCTGCCGTGCGAGCTGCGCATCGACCTGCCCGAGGCCGTGTACAACCACCCCGCGCTCGCGAACATGCGCCGCCTCGTCGCGGAGT
CGATCGTCCTGACCAACGACACGTACTCGTACAATATCGAGCAAGCCGCAGGGCACGACGGGCACAACATCGTCACAGTCGCCATGCGTGAGCT
GCATCTCGCGCTGCCCGCCGCGCTCGAGTGGGTCGGCGCGTACCACGCGAACATCCTCGCCGAGTTCGTCGAGTGCCGTGGCGAGCTGCCGTCC
TTCGCAGCCGAGGATGCGCAGATCGTCGAGTACGTCGAGGGCCTCGCGATGGGCGTGCGTGGGCTCGACGCGTGGTGTTTCGAGAGCGCGCGCT
ACTTCGGTACGCGCGGGCGCGAGATCCAGCGCGAGCGCGTCGTGGGGCTGCTGCCCAAGACGGTTATTGTTGGAGGGCTGGCGACGCCGATGAT
GGCGGGGCCTGCGGTGGAGGGGAATTCGGTGGTGGTTTGA

//
>AvSTS_09
ATGCTCACTTCTCCCGGACATTTCTATCTCCCCGACTTGCTCGCCCTCTCCACCCCCTTCCATTCTTCCACAAACCCCCACTACAAGGAGGCAG
CTGCCGAGTCGAGGGCTTGGATTAACAGCTACAATGTCTTCACCGACCGCAAGCGTGCCTTCTTCGTCCAAGGCTGCAACGAGCTTCTAGTCTC
CCACACTTACCCGCATGCGGGGTATGCACAGTTCCGCACCATCTGCGACTTTGTGAACCTTCTCTTCGTTGTCGATGAGGTCAGTGATGACCAG
AGCGGTGCCGACGCCCGGAAGACCGGGGAGGTGTATCTCAATGCCATGCGCGACCCCGAATGGGACGACGGCTCGCCGCTGGCCAAGATGACAA
AAGAATTCCGCGCTCGCCTTCTTGGCTCCTGTGGCCCGCGCTCGTTCGCCCGCTTCCTTAGGCATAGCGAGGACTACATCAACTGCGTAGCCCT
AGAGGCGGAATACCGCGAGCGCGGTGAGGTACTCGACATGGAGGCATTCAAGCATCTTCGCCGCGAGAACAGCGCCATCAGGCTTTGCTTCGGC
CTTTTTGAGTTTTCGCTCGGCATCGACTTACCAGACGAAGTATTCCAAGAGACCACGTTCTCCAGCATCTACTGGGCTGCCGCGGACATGGTTT
GCTGGGCAAATGACGTGTATTCGTACAACATGGAGCAAGCCAAGGGACACACCGGCAACAACATCGTCACCGTCCTCATGAAGGCCAAGGGTTT
CGACCTCCAGGCGGCCAGTGACTACATCGGCATCTACTACGCCGAGCTCATGAATCGCTACATCGCTGACAAGGCCCGCCTTCCATCATTCGGC
CCCTCTATCGACACCGACGTCCAGCGCTACGTAACCGCCATGGAAAATTGGCCAATCGGCAACCTGGTCTGGAGTTTCGAAACAAACCGCTACT
TCGGCCCTCGGCACGCGGAGATCAAGCGGACGCGTCTGGTCATACTCAAGCCGCGGGAGGAGGACACATGA
//
>AvSTS_11
ATGTCTTCTCCCACTCACTTCGTCCTCCCCGACCTTTTGTCGTTGTCAACACCATTCCAGGACGCGACAAATCCCCATTGGAAACGCGTCGCGG
CCGAGTCCAGGAACTGGGTCAACAGCTACAAAGTCTTCTCGGACAGACGTCGTGCTTTCTTCTTCCAAGGACAGAGCGAGCTCCTCACGTCGCA
CTGCTACCCGTATGCCGACGCAGAGGAGTATCGCATGTGCTGTGACTTCATTAACCTGCTCTTCACCATCGACGAAATCAGCGACGAGCAAGAC
CATGCTGGCGCCCTTCTCACCGGCAATACCTACCTTCATGTCTTGCGCGACCCGAGCTGGACCGACGGGTCCAAGCTAGCCAAAATGACTGCAG
ACTTCCGCAGTCGCTTTACGCGTAAAGCGAAGCCGAACACATTTCGCCGCTTCCTCGAAAGCAGCCAGCGATACATCGACAGGGTCGTAGAGGA
AGCTGATCTCCGGGAGCGAGGGGAGATCCTCGACCTCGACGCTTACCTCCTTCTGCGCCGTGAGAACAGCGCGGTCCGGCTGTGCTTTGATCTT
ATCCCCTATTGCCTCGGCATCGACCTGCCCAACGAGGTTGTGGAAGAGCCGACGTTCCAAAAAGCCTACTACGCATCCGTCGACATGGTCTGCT
GGGCCAACGACCTCTACTCGTACAACATGGAATTGAACCAAGGCCTCGAGGGAAACAACTTCCTGACGGTCCTCATCCAGAAACAGGGCATGGG
CATCCAGCGCGCCAGTGATTTTGTCGGGGAGCACTACAAGCGGCTCATGGACGATTTCCTGGACGCTAGCGCGCACATGCCCTCATTCGGGCCG
GCAGTCGACAAAGACGTGCAGCGCTACTTGCAAGCGTGCACGCACTGGCCCGCGGGGAATTTAGCGTGGAGCTTCGAGACGCCACGCTACTTCG
GCGCCCGTCGCGACGAGATCAAGCGCACGCGCATTGTGCCGCTCAAGCCGCTGGAGTTGGAGAAACTGGAGGAAGATTGA
//
>AvSTS_12
ATGGCCCTTTCTCAGTTTTCGACCCTCGTCTTCAACGCATTTTCTCCCACCAGAGATGTCTCTCCCGTATCATCCCTTCCGCCAACTAACTCAA
CTCGGACGGTATCCCCCACGCCGACGGTCGTAGAGCCCGGATCCTTCATTCTCCCCGACCTCGTTTCGCACTGCACGGTTCCTCTAATCTACCA
CTCAAATGGCGATGCCGTGGCGAAGCATTCGCTTGACTGGATGCTGTCGTTCGTCCCACACTTCACCCCCCGCAAGATTGCCGCATCGCGTGGC
TTGCTGGCCGGCGAGTTGACCGCGTTCTGCTACAGCAACTGCTCGTCGGAGCGCCTGCGCGTTGTCAGCGACTTTATGAATTTTCTCTTCCACC
TGGACAACATCTCCGACGGGATGATGGCGAGGGACGCCAAGGGCCTGAGTGACTGGGTGATGAACGCCTTTGAATGGCCTGAAGGCTTCAGACC
TCTCCCAGGGCAGAAGGGCGAGATAGAAGAGATCAGCGCAGCCAAGCTAGCCCGCGACTTCTGGTCGCGCTGCATCGTCGACTGCCCGCCAGCC
GTCCAGCAGCGATTCAAGTCATCGATGAATATGTTCTTTCAGGCGGTGCATCAGCAGGCAAGTGACCGCGCGAACGGGAACATCCCCGACCTCG
AGTCATACATCGACGTCCGTCGTGATACCAGTGGTTGCAAACCCGTCTTCGACTTGATCGAGTACACGCATGGCTTCGCGTTGCCTGATGCAGT
CGTCGAGCACCCGACCATGGTGGCCCTCAATCAAGGCGCCAATGATCTTGTGACGTGGTCGAATGATATATTCTCGTATAATGTGGAGCAGGCC
CGCGGTGATACGCACAACATGATCTGTATCTTTATGGCCCTTGAAGGCGCCACGCTGCAGGAGGCGGTAGACCGCGTTGGGAGTTTGTGCAAGC
AGACGATCGACGCGTTTGTGGAGAAGCAAAGGCGCCTGCCGTCTTGGGGAGAGGAGATTGACAGGATGGTCGATCTCTATGTGTTTGGATTGCA
GGAGTGGATTGTAGGGGCCCTGCACTGGAGTTACCAGACGCAAAGGTACTTCGGCGCGGACGGTGAGGAAGTGAAGAGAACTCGTGTCGTGAAC
ATATTGCCGTTGGAGAAGGCGTGA
//
>AvSTS_13
ATGGCCCTTTCTCAGCTTTGGAACCTCGTCTTCAACGCATTTTCTCCCACCAGAGATGTCTCTCCCGTATCATCCCTTCCGCCAACTAACTCAA
CTCGGACGGTATCCCCCACGCCGACGGTCGTAGAGCCCGGATCCTTCATTCTCCCCGACCTCGTTTCGCACTGCACGTTTCCTCTAATCTACCA
CTCAAATGGCGATGCCGTGGCGAAGCATTCGCTTGACTGGATGCTGTCGTTCGTCCCACACTTCACCCCCCGCAAGATTGCCGCATCGCGTGGC
TTGCTGGCCGGCGAGTTGACCGCGTTCTGCTACAACAACTGCTCGTCGGAGCGCCTGCGCGTTGTCAGCGACTTTATGAATTTTCTCTTCCACC
TGGACAACATCTCCGACGGGATGATGGCGAAGGACGCCAAGGGCCTGAGTGACTGGGTGATGAACGCCTTTGAATGGCCTGAAGGCTTCAGACC
TCTCCCAGGGCAGAAGGGCGAGATAGAAGAGATCAGCGCAGCCAAGCTAGCCCGCGACTTCTGGTCGCGCTGCATCGTCGACTGCCCGCCAGCC
GTCCAGCAGCGATTCAAGTCATCGATGAATATGTTCTTTCAGGCGGTGCATCAGCAGGCAAGTGACCGCGCGAACGGGAACATCCCCGACCTCG
AGTCATACATCGACGTCCGTCGTGATACCAGTGGTTGCAAACCCGTCTTCGACTTGATCGAGTACACGCATGGCTTCGCGTTGCCTGATGCAGT
CGTCGAGCACCCGACCATGGTGGCCCTCAATCAAGGCGCCAATGATCTTGTGACGTGGTCGAATGATATATTCTCGTATAATGTGGAGCAGGCC
CGCGGTGATACGCACAACATGATCTGTATCTTTATGGCCCTTGAAGGCGCCACGCTGCAGGAGGCGGTAGACCGCGTTGGGAGTTTGTGCAAGC
AGACGATCGACGCGTTTGTGGAGAAGCAAAGGCGCCTGCCGTCTTGGGGAGAGGAGATTGACAGGATGGTCGATCTCTATGTGTTTGGATTGCA
GGAGTGGATTGTAGGGGCCCTGCACTGGAGTTACCAGACGCAAAGGTACTTCGGCGCGGACGGTGAGGAAGTGAAGAGAACTCGTGTCGTGAAC
ATATTGCCGTTGGAGAACTCAGCGTGA
//
>LnSTS_01
ATGTCACCTTCGTCCGAGATATACTTCACTATCCCAAACACCCTCCAAAATTGGCCCTGGCCTCGCCATATCAATCCCAACTATAATGTATGTA
AGGCGGAATCTTCTGCTTGGTGTGAAGGATTCAAGGCCTTCACACCCAAGGCCCAGAAAGCCTTCAATAAATGTGATTTTAACCTTCTCGCATC
TCTTGCCTATCCTTTGTTGAACAAAGAGGGATGCCGAGTGGGATGCGACCTTATGAACCTGTTCTTCGTCATCGATGAATACTCCGATGTTGCA
GATCAATGTGGGGCCCGTGAACAGGCCGACATCGTTATGGACGCCCTTCGCCACCCACTCAAGCCCAGACCAGAGGGCGAATGGGTCGGTGGTG
AAATCGCACGACAGTTTTGGGAAAATGCTATCCGTACAGCGACGCCTTCAGCCCAGCGCCGCTTTATTAGAATGTTCGATTCATATCTCAACTC
CGTTGTCCAGCAAGCCGAGGATAGAACCCATAATTATATTCGCGATATACAAAGCTATTTCGACGTTCGAAGAGACACAATCGGAGCGAAGCCG
TCTTTTGCCATCAACGAGATACACTTAAATATTTCGGACGAGGTCATGGAGGACCCGATCATCAAGACCCTCACATCTACTTCTATTGATATGT

TGATCATTGGAAACGACTTGTGTTCTTACAATGTCGAACAAGCTCGAGGCGATGATGGCCACAATCTCGTGACCATCGTGATGAACGAACGAAA
AATTGGGCTACATGCCGCCCTCCAATGGATATCCGACCTCCATGACAGGTTAGCGGCTGAGTTTCTGGAAGCATACAAGAAACTCCCCTCCCCG
ATTGACCCTGATGTCGCCACCTATGTAGATGGGCTTGGCAACTGGGTCCGGGGCAATGATGTCTGGAGCTTTGAGAGCGAGAGGTATTTCGGGG
TCAGGGGTCTCGAAATACAGAACAGAAGAATTGTACAGCTTCTTCCAAAGGAGAAGGGTGAGCAAGGAAATACGGGGTTGATTCCCAGCACCCA
GGGTATAAGCGTATCAACAATAAAACCCTCAGGGTTTATACAAAACATACTCTCAAGCATCTGGATAAAATGGGGCGTTACATTCCTAGTGCTC
ATGCTTTCAGTTGCATCTTTACGTAGCATAATTATACTACATCGCTACGAATTATTTTAG
//
>LnSTS_02
ATGGCGGACGTCATTGTGTTACCTGACACACTCAGAAACTGGCCATGGCCACGCTCCATAAACCCTTTCTACGAGGTGTGCAAGGCGGAATCAT
CCAAGTGGTGTGAAGGATTCCGAGCGTTCAGTCCGAGTGCACAACGAGCTTTTAACAAGTGCGATTTCAACCTCCTAGCATCTCTTGCATATCC
ACTACTCAATAGAGATGGATGTCGTATTGGCTGCGACCTTATGAACCTGTTTTTCGTTATTGATGAACATTCCGATGTTGCCGACATGAAGACT
GCGCGTTCTCAAGCAAACATTATCATGGATGCCCTCAGAAATCCCCTGAAGCCTCGGCCAAAAGGCGAATGGATTGGTGGTGAAGTCGCAAGAC
AATTCTGGGAGAATGCTATCAGAAGCACTACACCCACAGCTCAGTCCCGATTTGTCGAGACTTTTCAAAGTTATACCGATGCGGTAGTTGAACA
GGCCAAAGACAGACACACCAATCACATCCGTGATATAGGGAGCTATATGGATGTCAGACGTGACACCATTGGCGCGAAACCATCTCTTGCAATC
TGCCAAGTGCATATGAATATTCCATCTGAGATCCTGGAGCATCCCATCATCGCTGAACTCACTAGACTTTGCATTGATATGCTGATTATTGGTA
ATGATTTGTGCTCCTACAATGTTGAGCAAGCCCGAGGAGATGATGGTCACAACCTCGTGGCAATCGTCTGTCATGAGATGAAATTTAGCCTTGA
GAGGTCATTTCACTGGATTTCCGTTCTTCACGACGATTTAGTAGAAAGGTTCCTCAGGGTGTGGAGAGATATTCCAACCTTCGGGGGCCCGATC
GATCGTGAAGTTCGCACATACGTCGACGGTCTGGGAAACTGGGTACGTGCAAATGATCAGTGGAGCTTCGAGAGTGAACGCTATTTTGGGAAGA
TGGGCTTGGAAATCATGCAAACCCGACGCGTCAAGCTTTTACCCAAACAGCACTGCCGCGATGTCAACCTTCCGCTCACGGAGAGAAGTTTGGA
AAAGCAGCTTCTCGAAAAGCGGCCAAAGGGGCCGATCGCTAAAGTAATTACTCTTTTTTTCTTCATTTTGATCGTATTTTCTGCAGGGATACAA
TGCTATTTTGTACTATAG
//
>LnSTS_03
ATGTCCACCCAATTTGTACTCCCCGATATCCTTGCCAGGTGGCCTTGGCCACGCGCTATCAACCCTTACTATGCTGAATGCCGCCTCTCATCAC
AAGAATGGGGAGCTAAATTTCAAGCTTTCACTCCAAAGGCCCAGTATGCATTTGATCTTTGCGACCCAAGTCTTTTAGCATCACTAGGATTTCC
GCGTACGTCGCGAGCGGGTTGCCGTGTTGTTTGTGACCTCATGTATCTTTTTGGGGTATTTGACGAACATTCAGATGTTATGGACGCAAGGTCC
GTTCGAAGATGTGCTGATGTTGTCATGGATGCTCTACGAAATCCTCAAGTTCCTCGTCCTAAGGATGAGGAAATAATCGGAGAGATAACACGGT
CCTTTTGGTGCAATGCTATCAAGACTGTGGGTCCCACAGCCCAGAGACGCTTTATTGAAGAGTTCTCAGTTTATACCGAATCCGTTGTTGAACA
AGCTCAGGACAGGGATCACTTTGTTTTTCGTGATATTAAGAGTTATATGGATATTCGACGCAATAACGTCGGCGCAAAACCCGCTTTTGCTCTT
CTTGAAATGGATATGGAATTGCCACACGAAATTTTTGATCATAAATCTTTAATTACTCTTCGCGAATGTACTGTTGACATGTTATGCTTGGCAA
ATGATATGTACTCATTCAACGTGGAACAAGCAAAGGGGGACGATCACAATATAATAACGCTAATCATGCTCCATGAAAAGCTGAACATCCAACA
GGCAATGGAATATGTTGCAGCCTTACACCAAATTTTTGCGGATAAATTCCTTGGCACTTATAATTCCCTTCCCTCGTTTGGCTCCCCAGTGGTC
GATGCAATGGTCCATCGCTACGTACAAGGACTAGGAAATTGGATTCGTGCCAATGACAGCTGGAGCTTCGAAAGTTGGCGATATTTCAAGGATG
AGGGTTTGAAGATTCAGAAGGAGCGTGTTGTAAAATTATTGCCCAAAACTAAATCCCTGGGCAGGTCAAACTGTGCGCACCCTGAATTACCTCG
AGACATATAG
//
>LnSTS_04
ATGGCTTTAATCACCAGTCTTAAATCCACAGAACGGACTACATTCGTCCTTCCTGACCTCCTTGCTAATTGGCCGTTTAATCCAGAGCCAAACC
CACGTCAAGATATTGTTGCTCAAAGCGCCGCATGGGTTGAATCTTTCAATCCTTTCGACGCCAAAGCTCAGAATGCGTTTAACCGGTGCAAATT
CGGGATATTCGCATCGCTGGCCTACCCCCATGCGACTGGGGCTCACTTTAGGGTAGCCTGCGACCTGATGAACTTGTTTTTCGTTTTTGATGAA
TATAGCGATCGCGCAGACGGTGAAGCTGTTGGAAAGCAAGCCGCCGACATAATGAATGCACTCAGATACCCAGATACCATACCCCCAGAAGGTG
ATAGTTTGTTAGGGGCTATGACCCGAGATTTCTGGCTTCGAACAATGAAATGTGCCAGCGAATCATCTGCACAGCGTTTTATTCGTAATTTCGA
CGAATATACGGACGCGGTCAGACAAGAAGCAGCAGACCGAGATGCGGGATGTATTCGAAGCGTCCCCGAATATTTCAAAATACGCCGTGGTACC
ATTGGAGTTCATCCATCATTTGATTATTACTTGCTGCGGGACGATCTCCCTGACGAATGTGTTAACCATCCTGACGTTCAAAGGTTGGCTAGCG
CCGCTGTGGATATGACCATCCTCGCTAATGACGTCTATTCGTATAACAAGGAACAAGCCAAGGGCGAGGACTCTCATAATCTTGTTGCGGTCGT
CATGAAAGAACATGACTTAACGGTTCAGGAGGCTATGGATTATATTGGCGACCTCTATAACCATATCCGCAAACAATACTGCGAAAAATTCCAG
GATTTACCACGGTTTAATGATGATGTAGACGGTTTAGTTCGAGAATTCTGTTATGGGACTGGGATTTGGGTGACGACGAATATCAAGTGGAGTT
TTGCGAGCGAGCGTTATTTTGGTAAGGAGGGCATGGAGATAATGAAGCATAGAACTGTTACTTTACTTCCAAAGGTAGATAAACTGTTTAAATA
G
//
>LnSTS_07
ATGTCTTTCCACCTCCCGGATATCCATCGTGACTGGCCATTCCCTCGTGCTGACAACCCACACTACGTCGAAGTGGCGTCAGAGAGCGTACGCT
GGATTGAGTCGTTCAAACTTTTCCCGACAGATCGTCAAAGGTCGTTTCATGCAATCTTACCAGGTCTCCTAGGGTCCATGGCATATCCAAACCT
TTCTCGTGCACATTTCCGAACTGCTTGTGACCTCATGAATATTCTTTTCGCCGTCGACGACATAAGCGACCGCCTCAACCCTACAGAAGCCAAA
ATATTAGCAGACGCCATGTTAGATGCACTCAGGAATCCCGAGAAGGAGAGAGACGTTACAGAACACCGTCTTGCAGATCTTGTTCGAAGCTTTT
GGGCTCGGGCATTGGAAACGGCCAGTAGCACAACAATACGTCGCTTCATCCGGAGCTACGAGGATTACGTGTGTGCAATGACTCAAGAAGCACA
AGATCGTCAGTACAAGAGTGTACGCAAGTCAATGGATGAATACCTAGATCTTCGACGCTACACCGGTGCCATCAAGCCTTCTTTAGACTTAATT
TTGCTTCCGCTGGAAATCTCGGACAAGCTCTTGGACAGTCCCACTGTCAAGGAGTTGGAAATGATTGCAATTGAGCTCATTGCGGTGGCAAATG
ATATTGTTTCATTTAATGTGGAACAAGCGAGAGGTGACATACACAACTTGGTCATTGTCCTAATGGACGGGGACAAGAAAATGACCGTCCAAAC
AGCAATGGATTTGGTGGGGCAGTGGTACAGAAAACGTGGTCAAGATTTCGTCGAAGGGTTGAAGCGACTTCCCAGCGTTGACGACGGCAACTAC
GAGATACTCTGCCTTCATCAATATGCATGGGGTCTAGGTAACTGGGTGACTGCTAACTATGAATGGAGCTTCGAAAGCCATCGCTTTTTTGGGG
AGGAAAACGGCGAGGTTATGAAGTACCGTGTAGTCAAACTACTGCCTAAATCTCCCCCGCTCGTAACCGTTTGA
//
>LnSTS_08
ATGCTCACCTCAAGTCACCCTACTTCTTTCATGCTCCCAGACCTCGTTGCACATTGCACTTTCCCCCTCTCCTACCACCCTAACGGCGATGATA

TCGCCCAGCAATCAGTCGCGTGGCTTGATTCTAACTGCCCAGACTTGACGCTCAAACAAAGACGCGCGCTCCGCGGTCTTCAAGCGGGGGAGCT
TACCGCATATTGTTACAATACAACAAGTCCCGAACGTCTACGCGTCGTGTCTGATTTTATGAACTACCTCTTCCATCTGGATAACATCAGTGAT
GGGATGATGACAAGAGAGACTGATGTATTATCGGATGTGGTCATGAACGCCCTCTGGTTCAGTGATCACTATCGACCCACCCACGGCCCTGGCA
AGGAACAACCCGATGAAGAACTCAATCCAGGAAAGTTAGCTCGAGACTTCTGGGCTCGGTGCATCCCAGATGCCGGTCCCGGCGTCCAGGCGCG
CTTTAAAGAGACTCTCGAGCTGTTCTTCGAGGCTGTCAACATTCAAGCTCGGGCTCGCGATGACGGGATCATCCCTGATCTCGAGTCTTATATC
GATGTCAGAAGAGACACTTCAGGCTGCAAACCTTGTTGGGCTCTTATCGAATATGCTCTCGATATCGATCTTCCAGATTATGTCGTCGAACACC
CCATCATCGAAGCCCTTAATCAAGGAACAAACGATCTCGTCACTTGGTCGAATGATATTTTTTCATATAATGTCGAGCAATCTCGAGGAGATAC
TCATAATATGATTGTCATCCTCATGAAGTATCACGGGCACGATCTGCAAAGTGCAATTGATTATGTTGGCAACTTATGTGCCCAGACAATCGAC
GCGTTCAATGAGAACAAAAGCCGTCTTCCATCCTGGGGCGCCGAAGTCGACGAGATGGTCGGACGATATCTCAAAGGGCTTCAAGATTGGATCG
TCGGCTCACTCCACTGGAGTTTCATGACACATCGGTATTTTGGCACGAATGGTGCAGAAGTTAAGCAACATAGACTCGTAAAACTCCTACCCCG
TGAACAATGA
//
>LnSTS_09
ATGAATCATACTACAACACAGATCCTCCTTCCCGACTTGATATCAATGCTCCCATTGGAAGGAGCCACCAATCCCCACTACGAAAAAGGGGCCG
CCGAGTCTCGAGCCTGGATCAACAGTTACAATGTATTCACTGACAGAAAGCGCGCTTTCTTCGTCCTTGGCTCTAACGAGCTGCTCGTCTCCCA
CGCATATCCATACGCTGGATACGATGTTTTCAAAATCTGCTGCGACTTTGTCAATGTCCTCTTTGTGTTCGACGAGCTCAGCGACGAACAAGAT
GGAAAGGACGCACTCACTTTGGGTAACATATTCGTCAATGCCATGACGGATCCCTTATGGAATGACCAGTCAAAATTCTCGAGGATGACAAAAG
AATTTCGCAGTCGATACACAAAATTGGCTGGCCCGAACACTACTGCTCGCTTCCTGAAATACTGGGAAAGCTATTGTGCCGCTGTTATCACAGA
GGCCGAGCTCCGAGAGAAAGGCCAAATTCTCGATGTCGACTCGTTTATGGAACTGCGACGTGATAATAGTGCCGTCCGCCTTTGTTTCGGACTC
ATTGAATTTTGTTTCGGAACCGACCTTCCCGATGAAGTCTTTGAAGATCCCACATTCCTCAAAATATATTGGCCGCAGTTGACCTTGTTTGTT
GGGCAAATGATGTATATTCCTATGACATGGAACAAAGCAAAGGGATCAGTGGAAATAATATTGTCACTGTCCTCATGCACGACAAAAATATGGA
CCTTCAAACAGCCGTCGACTATGTTGGCACTTACTCCAAGGAGCTCGTAGACCGCTTCATGGACTTACAAGCGCATCTACCATCCTGGGGCAGC
GCAGTTGACTCCCAAGTTGCCCTTTTCATCAAGGGACTTGGTTATTGGGTCAAAGGAAATCTCGATTGGAGCTTTGAGACACAGCGTTACTTTG
GCCCAAAACACATGGAGATCAAAAACACATTGCTCGTGACACTTCGGCCGCTAGAATGCCCCGAAGAAACAGACTCAGAATCTGATACTGAGTG
A
//
>LnSTS_10
ATGTCTGCCTCGACTTCAACAACAACCAAGATTATCCTCCCTGACCTCGTTTCTCACTGCACCTTCAAGAAACGCGTCAACCGCCACCGAAAAC
AAATCACCACTGAAACTAAGCAGTGGCTTTTCAAAGGGGGGAACCTAAACGGGAAGAAGCGTGATGCGTTCCACGGCCTCAAAGCTGGACTCCT
CACTGCTATGTGCTACCCCGACGCTGGTTGTCCTCAACTTCGGGTATGCAATGACTTCCTCACCTACCTTTTCCACCTCGACAATCTATCCGAC
GACATGGACAATCACGGTACTCGTTCAACCGCAGATGAGGTTCTCAACTCCCTGTACCACCCGCACACATACCGTTCTTCCGCCCGAGTGGGAA
AGATGACTCGAGACTATTATAAACGCTTAATTCTCACCGCATCGCCTGGAGCTCAGCAGCGCTTCATCGAAACGTTCGACTTTTTCTTCCAGAG
TGTAACTCAGCAGGCATCTGACCGTAAAGATGGTCTTATTCCTGATCTTGAGTCCTACATTGCTCTTCGTCGAGATACGTCTGGGTGCAAACCA
TGCTGGGCACTCATCGAATACGCGAATAACCTCGACATACCAGACGAAGTCATGGAACATCCCATCATCCAAAGTTTAGGTGAAGCGGCAAATG
ATCTAGTCACGTGGTCTAATGACATATTCTCATACAATGTCGAACAATCAAAAGGCGACACACACAATATGATTCCAGTCGTGATGAACGAAGA
AGGTCTTGATCTACAAGCTGCTGTTGATTTCGTTGGAAATATGTGCAAGCAGTCCATCGACCGTTTCGTGGACGATCGGGCCAACCTTCCTTCT
TGGGGCCCTAAAATCGACCGCGATGTCGCTCTATATGTTGACGGGCTTGCAGACTGGATCGTAGGGTCTTTGCACTGGTCATTTGAATCCGAAA
GGTACTTTGGCAAGACAGGGCGCGAGGTAAAGACTACTCGCGTTGTGAATCTCCTCCCCCGCCGAGTCTAA
//
>LnSTS_18
ATGTCCCCTGCAAGAATTAAGACATTGCCGGATGTACCTGAGTGTTCCGAGATCCGTGACTGTGTGGCGGATTTCCTAGGCCGATGCAAGATAG
GGTACAAGCACGCTCCCATAGATCCATCTTTCCTAGAATGCTGCTATCGAGAGGCAAAGTCCCGTGGTTATGTCACAGAAGACGAACAATCGTT
TCTTCAGTTCCTTCCTGGTGGAGCTGCCATGGCTCTCACTGGATACGCACACCTCCCAGATATGTCTATTCGTGTTTCTATAGCTCTTTACACG
GCGTGCGGGATATATTTGGACGACAAATTCAAAACAGATGTCGACGCTGTTGCCAAGTTCAACGAACGGTTTATTCGTGGTACCCCTCAAGAAG
ATAGGGTTCTCGACGCTTTTGCTGATATCATTCTCGATATCCTCCATCGGTTCCCGCGAGTGAGTGGGAACATGATTGTATCTTCAACCCTAAA
TGCGGTCAATGCACTTCTGCTTGAATATGAAACTCGTAATATGAAGGTATCAAATAATGCAGACAGCTATCCAACTTTCTCCAGAATAATGTCT
GGAGCTTCAGAGAGTTATGCGTTATTTGTGTTTCCCCACACCATTCCCGTCGAAGACTATATTCAAGCCCTTCCTGAACTTATGATATTTATCA
ACAACGGCAACGATATTCTCTCATTCTTCAAAGAGGAAGCGGATGGAGAGTCTGTGAATCGTATATCCCTACTTGCTGCGTGTCGTGGCTCCAC
CAAGGCCGAGGCTCTTCGGTGCCTCGTAAATGAAGCGGTTACAGCTAACGACAAAGTCCTTAAGATCTTGGAACCTCATTGTGATTCTTACGAC
GCGTATCGTAGATTTAGTAGTGGCTTCATTGAGTTTCACGCAACGTTGAAGCGGTACCGTTTGGACGAACTAGAGCTCTAA
//
>LnSTS_19
ATGGCTTTGTACTCTTTGCGTTCATTGTTGCCTACTCTGTTCTCGTTTGCGATTTCGCCCATTTCACCGCTGGATTCTTCCATGAATTATCACA
CATATCGTCTTTCAGATGAAGAAATAGAACGCTCGATATACCGAGATTTCCTCAGGAAGATGGAATATTGCAAACCTGATTTGCATGATGCCGA
AAAAACTTTGGAACAGGCCTTGAGATCTGAAATGTACAGCCGGAACATGCAATGCCCCCAGCTCGAAAAAACAGTCCATCTGGCAGCCCGCCTC
GTGGAGTTAGCATACCCAGACTGCACATTCGAAGAGCAAAAAGTCATAGCCCTCGTGAACTGGTTCGTTATATACCTAGACGATGTCGACAGCG
AACCATGTGCCGCCTTTGTCCCCCGCCTCCTCCGAGGCCAAAAGCAACTCGACCCTGTGCTTGATGCTTTTGCCGACGTTCTTAACTCCATGCA
CGACTACTACGACTCTCTCGCCGCCAACCAAATCGTGACCTCCATCCTCAACTTCATCAACCTGACCCCCGTCGAGTCACAGATCGCCGCGGGC
ACCTTCACCGTGCCCTCGCGGTCCAGACGCTTCCCGGGGTTCCTCCGCGATCGTTCCGGGCTAGGCGTGCCATTCGCGCTTTTCGCATTCCCGA
AGTCGCAAGGGCTGGACACAGCAGCGTACCTCCGCGCTCTCCCCGATATGGACTTTTGGATTTGCGCCGCGAACGATTTGCTATCATACCACAA
GGAGATGCTCGCTGGCGAGACAAATAACTATGTGTCGAGTAGGGCACTCGTGGAGAAAAAGACCCCTCTCCGGGTATTGGTTGAGCTGGAACGC
GAATTAGAGGAGTCCCGGAATGTGATACATGCGACTCTCAGCTCGCACCCTGCTGCTATCAAAGCATGGCGCGCCTTCGAGGTTGGCGGAGTTG
CATGGCATTTGGAGCAAAAACGCTATAAGCTCAAAGATCTGGCATTAGATCAACAGGTTACTGCGAGGGGGTGA
//
>LnSTS_20

ATGGCCCCAGTTTCTCTTATTCGCCCTACATCTGCCAATGTGGTGCAGGATCTAAATAAAACTGGAATATTGAGAAGGTTTCTTCAAGGAATGG
CTTATTGTCCATCAAATATTTCCGGTACAAATGAGGAGCTTGAAAAAATTATGCGTGCTGAGATGAATAACCGGAATATCCGTTGTCCTCAGCT
CGAAAAAACTCTACATCTTGCTGCTAGCCTAATCGAACTGGGTTATCATGACTGTACACTTACAGAAAAAACGAACATCGCCCTTTATAATTGG
TACCTGATATATATCGACGACATGTCTTCTAAAGACACAGGCCCGTTTATGGCCTTCCAAGAACGTTTCCTTCGTCGTATGCCGCAACTCAACC
CAGTACTCGAAGCTCTCGTCGGGGTTCTCATGCGCATCTACGAGCTATATGACATGCCAACTGCAAATTCCATCTTGAGCGCTACCTTCAATTT
CGTCAACTCTACTTGTATAGAGCCCGAAATTGAAACCCTCCCCCTTACGCGCGGCTTGGTTCGCTTTCCGTGGTTCCTCCGTGATCAGACTGGC
GTGGCAATCGCGTTTGCGCTTTTGCTGTTCCCCAAATCGAAGAATGTCGGGGTCACTGACTATATACAAGCGCTATCCGACATGAATTTTTGGA
TATCAGTCACGAATGACATCCTATCGTTCCACAAGGAAGAACTTGCGGGGGAAAGGGCTAATTACGTTTACAATCGAGCCTATATCGAAGACAA
GGTACCCTTGAGCGTCCTTGCTGAGATGAGCCAGGAGCTCTTTGAATCGCGAAATGCGGTTTATGCAGCTCTGGAGCATTGTCCGCAAGCTGCT
GACGTCTGGCACACCTGGGAACAAGGTTATGTGCGATGGCACATAGACCAGAAACGGTATAAATTAAGCGAATTATCTTTGTAG
//
>LnSTS_21
ATGGATTTCTCCACACCGAACATTAACGGCACAAACACAAAGCTCGAAGCAGCTATGACTGAAGAGATGGATACGCGCAACCTTCGTTGCTATC
AGCTCGAAAAGACATTACATCTGGCAGCAAGCTCCACCGAGCTGTGTTATCATACTTGTGACTTCGAGGAGAAGAAGAGTATCGCACTCTATAT
CTGGTACATGTTCTACGTCGACGATATGGCTTCTCGTGACGTCGATTCCTTTGCACCATTTCAACAACGCTTTCTCCGACGATCTCCACAACTC
GACCCTGTCCTAACCGCCTTTGCCGATCTCCTCCTTCGAATGTGTGACCAGTACGATACAATTACCGCCAACTCTATCATTTCCGCCACATTTG
AATTCGTAAACTCCACCTGCATCGAGTCCGATTTAGAGCGCATGCCTCTCGTTCAAGGCGTGCATCGTTTCCCTTTGTTCATTCGTGACCGAAC
CGGTGTATCAACCTCCTTCGGCCTGATGCTTTTCCCACTCTCCAAGAAGATCCGTGTAACGGATTACATCCAGGCACTACCCGATATGATCTTT
TGGATTTCTTTATCAAACGACCTCTTGTCCTTCCACAAGGAAGAACTAGCGGGAGATACAAACAACTATGTTCATATTCGAGCTCTCATGGAAG
AGAAAACTCCCATCGAAGTCCTTTTTGAGATGGAGCAAGAGCTAACTCTGGCACGGCAAACAATATACAAGGCTTTGGCAGGTTCCCCTACCGC
CACTGCCGCCTGGTTAGGATTTGAGCGCGGATATGTTGCATCGCACCTAGCCCAAAAGCGATACAAGCTCCAAGACCGGGGACTTTAG
//
>LnSTS_25
ATGTCTCAAGACACTAGCAAAGCGAAATTTGAAATGGCGGCAGTCTTGAAAGAATTTCTTCAAGAAACTGCGTATCACCCACCCACTATCCCGA
GTACTAATGAAGAGCTCGAGAATAAAATGTACGAGTACATGCATGGTCGCAACCTCGCATGTCCTCAGCTTGAACGGACGTTACATCTTGCCGC
TAGCCTGATCGAAATAGCATTTCCGGAATGTACCTTATCGGAAAAGACCGTCATTGCCATCTTCAACTGGTTCATTATTTACATTGACGATACT
TCTCCTGGGGATGTTAGTCCTTTTGTTGCCTTTGGCCCACGTTTTTTCAGTCAAACTCAGCAACTTGATCCCGTCCTTGATGCCTTCGTTGATA
TTTTGCAGCGCATCTGTGACCAGTATGAACCCGCCACTGCAAATTACATTCTGGCCTCGGCATTCAATTATATCAGCTCCACTTGCATCGAGCC
CGAGATTGAGGCACATGCCGTCGTACCCGGTGTCATTCGATTTCCTTGGTTTATACGTGGCCAGACTGGTATATCACTAGCCTTCGCCTTGATG
CTATTTCCGAAATCTAAGGGCGTCAGTGCCGTCCAGTACATCCAAGTCTTGGAGGAGATGAATTTTTGGATATCAGGTGTCAATGACCTTATGT
CGTTCCCAAAGGAACAGCTTGCGGGAGAAAGAAACAACTATGTGCATGTTCGAGCGCGCACAGAGGAAAAATCCCCAATGGAGGTTTTGGCTGA
GTTAAACCAGGAGTTGCACATGTCGCGAAAGTTAATATATGCTGCGTTATCTCTCATCCCTGGAGCTAGTACCATTTGGTCTGCGTTTGAGAAT
GGATATATCAAATGGCATTTAATCCAGGAGCGGTACAAGTTAGCGGAAATGGATTTGACTGGTTGA
//
>LnSTS_27
ATGAGTACAGTCACCCAACTTTTGAATAACTTCGTGCACGTTGTCTCGCGAGCTGGAAGCTTGATCACTCCTGATTTCCTGAATGGGGAGTTTT
ACGATGCTCCACTGAGTGACGCGGGCGATCCTCCTGCCAAGGACGACGTGACCGATCTTATGGTCACAGTTGGGCAAATTCTTGGTAAAATGCT
CCATGACTGCCACATCCCGTACTGCGCTGTGTCCTTCGACTACGGACTCATGAAAGCGCCTGTTGCCGAAGCCGAGCGTCGTGGATATGCGCTC
GAGGGTCCCAACTCAATGCGCCAGGCCCTTTTCCTGGGAGTATACCTCACGTCCACAATGTACGCTCACGTCACTGACGATGACGCGCTGCGCA
CATATATTATCTTCTACATCATGTTTATGTTCTATGTCGATGACAAGTACTTCGAGCAGTCAGACAAGGAGAGTGGACTCCTCCACTTCATCGC
GCGGTTCAATCAAAATCAACCCCAGGCAGAGCCATCGTTAACTAACTTCGCCGATTTCCTGCGCGACGACACGGCCGCCGTCTTTGAGCCAATT
GGTGGAGGCATAATTATCACATCGACGCTTAACTTCATCAATGGGATGATTTTGGAGACTTCAGTTAAGCTTGGGCAGATATCGCCGTACGCTC
AAAACTTCCCTTACTTTGTCCGTAACAAGTCGGGGTTCCCCGAAGCCTGTGTCATTCTTGCTTTCCCTAGAGATATGCCTGTGAGGCAGTACGC
TCAAGCCTTCCCAGAAATCTGCGATTATCTGTGCTATGTCAATGATATTATGTCTTTTTATAAAGAAGAGCTGGTTGGAGAAACAGAAAACCTC
GTCTCACTTCTCGCAGCAGTGACTGACTCCAAGGTAGAGCACTTGCTGTCCACCGATTCACCGGCCCCCGTCACCCGCAACGACAAGTATGGTG
TCCTCCGGCGTCTCTCCGCTGCCGTTGCCTCAGGGCACAAGAAGACCCTTCAAATATTATCGGGAAACCCCGTTGCTCAAGATATCTACCTCAA
GTTCGCCATTCAATATGTCACAATGTATATCTCTATGAGGGAAAGGTACAAGTTGGATGAGCTTCGTTTTGGCGATAAGGCGTAA
//
>PoSTS_01
ATGTCAGCCATCGCATCCTCCTCCGCCCAGGCGGCCCCGCCTAGCAAGATTGTTATCCCAGACCTCGTCTCTCACTGCACCTTTGAGCTTCGAG
TCAACCGCCATCGCAAAATCGCCTCCGCGCAGTCAAAGCGATGGCTCTTCCGCGGAGACAACCTTACTGGCAAAAAGCGGGACGCGTACCATGG
ACTCAAGGCTGGCCTGCTGACTTCTATGTGCTACCCGAACGCGGGTGCCCCTCAGTTGAGAGTTTGTTGCGACTTCATGAACTACCTGTTCCAT
CTCGACAACCTCTCCGATGACATGGACAACCGGGGTACAAAGTCAACAGCAGATGTCGTACTGAACGCACTTTATAACCCGAAGATGCGTCAGA
CGAAACGTGTTGGCAAGATGACCAAGGATTACTGGCAGCGACTTATTCGCACCGCTGCTCCGGGTGCCCAGCAGCGCTTCATCGAGACTTTCGA
CTTCTTCTTCCAAGCTGTATCTCAGCAAGCGCGGGATCGCGCTGAGGGTTCAATCCCCGATCTCGAGTCCTACATTGCTCTGCGCCGCGACACA
AGCGGATGCAAACCTTGCTGGGCTCTGATTGAATACGCCAACAACCTGGACATTCCCGACGAGGTCATGGACCATCCCATCATCCGTAGCCTCG
GAGAAGCCGCCAACGACCTCGTTACCTGGTCCAATGATATCTTCTCATACAATGTCGAACAATCCAAGGGTGATACGCACAACATGATCCCTGT
TGTGATGCATGAGGAGGGTCTCGAACTCCAGGCCGCAGTTGACTTCGTGGGTGCACTTTGCAAGCAGTCGATTGATCGCTTCGTAGAATGCCGC
GCGAACCTGCCTTGTTGGGGCCCTGAAATTGATCGTCAAGTCGCAGTATATGTTGAGGGGTTGGCCGATTGGATAGTTGGTTCACTACACTGGT
CGTTTGAGTCGGAGCGCTACTTCGGCAAATCCGGGCTCGAGGTGAAGAAGAACCGCGTCATCAACCTCCTCCCCCGACGGTCATAA
//
>PoSTS_02
ATGTCGACGTTAATCTCTCCAGATCACTTCATACTTCCTGACCTCGTTTCCGACTGCACCTATCCTCTGCGCGTCAACGACAACTGTGAAGAGG
TGGCGCGCGTCTCGGAACAGTGGCTACTTAACGCGGCGAATCACAATGAGCGCAAACGCCGTGCTTTTCTTGGGCTCAAGGCAGGCGAGTTAAC
GGCAGCTTGTTACCCCGACGCCGACGCCTTCCACCTCCGCGTATGCGTAGACTTCATGAACTATTTGTTCAACCTCGATGATTGGCTCGATGAA

TTCGATGTAGAGGGCACCCGTGGAATGCATGACTGCTGTATTGGTGTGATGCGCGACCCGCTAAATTTTGAGACGGATAAGCGCGCGGGTATCA
TGACAAAGTCATTCTTCAGCCGATTTATCGAAACCGGCGGCCCTGGGTGTACAGAACGCTTCATTCACACTATGGATCTCTTTTTCAAGGCTGT
TGCCATTCAAGCGGCTGATCGCGAGAAGGATGTCATTCCTGACTTCGAGTCTTACATCACCATTCGTCGAGATACGAGTGGTTGTAAACCTTGC
TTCGCCCTTATTGAATACGCCTCTCGAATCGACCTTCCTGATGAGGTAGCTGAGCATCCATTGATTCGCTCAATGGAAGAAGCGACGAATGACC
TAGTGACATGGTCAAACGATTTGTTCTCTTACAACGTCGAGCAATCGAGAGGAGATACACACAACATGATTCCGGTCATCATGCACCAACGTGA
CCTGAATCTCCAAGAAGCAGTAGAATATGTCGGAGCTCTATGCAAATCAAGCATCCAGAGGTTCGAAACCGATCGCAAGAACCTCCCAACATGG
GGTCCCGAAATTGATCGGGACGTGGCAGTTTATGTCGAAGGACTCCAAAACTGGATCGTCGGTTCCCTGCATTGGTCCTTCGATTCGGAACGCT
ACTTCGGTACCTCCGGTCTTGAAGTGAAGCAACAGCGCATAGTCAAACTCCTTCCAAAAGTTCCATCATGA
//
>PoSTS_03
ATGGTCCCAGTCGCTTCCTTCATCCAAGAGCCTTCACGAGCACCTTCCTCCTTCATACTCCCGGATCTTGTTTCCCACTGCAAGTTCCCTCTTA
CCTACCACCCTCAAGGGGATGCTGTTGCCCTCGAGTCCGTTACATGGCTCGACAGACTATGCCCAGACCTCTCTCCCAAGGCCAGGAAGGCCCT
CTGGGGCCTCCAAGCTGGAGAACTTACCGCATACTGCTACCCCAGCTGCTCGCCCGACCGCCTCCGAGTTGTCAGCGACTTCATGAACTACCTC
TTCCACCTCGACAACATCAGTGACGGCATGATGACTCGCGAAACCGACGTCCTCGCTGATTCTGTCATGAACGCCTTGTGGCATCCCGAAGAGT
ACAGACCCACTCGTTCCCCTGGGAAGGAGCAGCCTGCTGAAGAGCTCGACGCTGGGAAGATTGCTAGAGACTACTGGTCCCGTTGCATCCCAGG
CGCAGGTCTGGGCGTGCAAGCTCGCTTCAAGGAGAGCCTCCAGCTTTTCTTCGAGGCCGTCAATGTGCAAGCTCGTGCGCGTGACGCAGGGGAA
GTTCCAGATCTCGAGTCGTACATTGATGTTCGCCGCGACACGAGTGGCTGCAAGCCCGTTTTCGACCTCATTGAATACGCGATGGACTTTGAGC
TGCCAGAAGAAGTTGTCAATCATCCTGTCATCAAGGCCCTTAATCAAGGAACCAACGACCTCGTTACTTGGTCGAACGACATCTTTTCATATAA
CGTGGAACAGGCTCGAGGTGACACGCACAACATGATTGTTATCTTGATGAAATACCACGGCCACACGTTGCAAAGTGCCGTCGACTATGTCGGT
GAACTTTGTTGCCAGACGATCAACAACTTCTGTGCCAACCGCCAAGTGATTCCCTCGTGGGGACCAGAGACCGATAGAATGGTCCAAGAATACG
TGCAAGGCCTCCAGGACTGGATCACTGGCTCTTTGCACTGGAGCTTCAAGACACACCGTTATTTTGGCACCAATGGTGCCGAAGTGAAGAAGAC
GAGGCTTGTCAAATTGTTACCCTTGAAAAACGGGGCAAACCCATGCGCCGGCGAGGCCACGAAGTGA
//
>PoSTS_05
ATGTCTTCCCAGCCAACTCAAATCGTCCTCCCTGACCTCCTGGCCATGTGCCCTCTGAAGGGATACACAAACCCTCACTACAAAGAAGCAGCCG
CAGAATCTGTCGCGTGGATCGACAGCTACAACGTCTTCACTGACAGGAAGCGTGCCTTCTTCGTCCAAGGTTGCAATGAGCTCCTTGTCTCCCA
CACATTCCCATACGCACCCTACGAGCAATTCAGAACGTGCTGCGACCTGGTCAACTTACTATTTGTCATCGACGAAGTGAGCGACGACCAGAGC
GGGAAGGATGCCCGTGCAACAGGCCAGGTATTCCTCAACGCTCTTGGTGACCCAGAATGGAACGATGGTTCTTTACTGTCCAGAATCACAAAGG
ACTTTAGGGCCAGATATTTCCGCCTCGCCGGACCAAATTCCTCTCGTCGCTTCTTGAAGCACTGCGAGGATTATATCAACGCAGTGTCCACCGA
GGCTGAACTCCGTGAAAGGGGCGAGGTCCTGGACATCGAGCCCTTCACTGCTCTCCGTCGCGAAAACAGCGCGATTCGCCTCTGCTTCGGTCTC
TTCGAATACGCACTTGGCATCGATCTACCCGATGAAGTATTCCAAGACAAGACATTCCAAGATATGTACTTTGCCGCCGTTGACATGGTTAGCT
GGAGCAACGATGTATACTCCTATAACATGGAGCAAGCCAAAGGACACAGCGGAAACAACATCGTCACCGTCCTCATGAAATCCAAGGGAATGGG
AATTCAAGAAGCTGTGGACCACGTTGGCGTTCACTTCCAACAGATTATGGACATATATATGGACTCAAAGACGCGTCTACCATCATGGGGGTCT
GAAGTTGATGTCAATATCGCCCGCTACGTCGAGGCACTTGGCCATTGGGCAAAAGGAAACCTTGACTGGAGTTTCGAAACTCAACGTTACTTCG
GTGCTGAGCATCTCGAGGTGATGGCAACCAGAGTCGTTACTCTAAGGCCCCACGGCTCGCCCGAGGATTTCGACGAATAG
//
>PoSTS_06
ATGACCTCGACTACGAAACACCCCCTTCCCTCGCACTTCATCCTTCCAGACCTTCTTGACCAATGGCCTTTTGAAACCGAGCCCAATCCCCACC
AAGAAATTGTCGACGATAGCGCTCGCTGGGTGGAATCCTACAAGGCCTTCAGTCCAAAAGCCCAAGACGCCTTCAACCGCTGTAACTTCGGCAT
CTTCGCGTCGTTGGCGTACCCTAGATCAGAAGGAACACATTATCGAGCCGCCTGCGATTTGATGAATCTGTTCTTTGTCTTCGACGAATTTAGC
GATGCCGAAAACGGAGACGTCGTGCGTCAACAGGCTGCTGATATTATGAACGCCCTCAGGTACCCTGACAAAATCCCAGCGGGTGGCGACAGTA
TACTTGGAGCCATGACCCGAGACTACTGGAAGCGGACTCTCGAGGTCTCAAGCAAGTCGTCTGCCGAACGCTTCATCCGCAACTTCGATGGATA
CACCGACGCGGTTAGGCAAGAGGCTGTTGACCGCGACGAAGGCCGCGAGCGATCGATTGAAGAATATATGGAGCTTCGCCGTGGAACCATCGGC
GTTTACCCATCCTTCGATTATTTTCTCCTCGAGGACGACATCCCTGACGAGTATATCGACCATCCCGCCGTGGCAAGCCTAGCTCTGGGGGCGG
TGGATATGACAATATTGGCGAATGACGTATATTCGTGGAACGTCGAGCAGTGCCGCGGGGAGGACAGACACAACCTTGTCGCCGTAGCCATGCG
CGAGAAAGGGTTATCGGTGCAGGAAGCCATGGACTACGTTGGGACCATCTACGCTGGTATCCGCGACAAATATGTCAAGGAATTCAACGAGCTT
CCCCAATTCCCTGAAAAATATGACAAGCTCGTCAAGGACTACTGCTGGCACATGGGGAATTGGGTGACCACGAATATCAAGTGGAGCCACTTCG
GCGAGAGGTACTTCGGGAAGAAGGGTAGAGAAATCTTGAAGCATCGGACGGTGGAAGTGATGAGACCGTCTGTCCTTGGGTGGGTCATGAGGAA
GTCATACCCCCTCATAGTGACGGTCAAATACGCACCCAGGGAGTACTACTACATTGCGGTGACGTTCCTCATGCTTCTGTTGGCTCTACTAAGT
TCCCCTTCGAGAGCAAGGATCCTTGGACATTCTTCTTTTCCGTCGTCCTTTTGA
//
>PoSTS_09
ATGAACAGAGGTGCAAGCTACCAGCTTCCAGACCTAACCTCCCTTGTCCGCGACTTTGAGCTGCACACAAATCCTCATTGCCACGCCATTTCAC
TCACCTCTGGACCAGGAAAGTTAATCGATGAGCGCGATAAAGCGTGTTGGCGTGGAATGAAGGCGGGGTTGCTGGCATCATTGTGCTTCCCGAT
ATGCGACGCCCCGCAACTGAAAGACCTGGCCGACATCATGTCTGCGTTCATCTGCATCCAAGTGCGCTTAGTGGGTGCATGCGACATGGCAGAA
TGTGGGTGGACGTCTTCGGAGGGGGAGGGTCAAGCGGTGTCGGGAGGCTGGGAGCTCCTTGCGCAGAATGCCTTACTTGCTCGATTGATCCCAC
AGGTGAAGCGGCTTGCCGCGCGTGCGCCAGCAGACTGGTCCTCACGGCTGGAGAAAGCATCGCACGCTTTCCATGCCGCCCATCTGCAGCTAGT
CGCGAAATCCCCGCCGCCGGATAGTATCGAGGCATGCTTGGCTCTCCATACAGACGCCAGTGGCTTCCGTATAATATTCCTCCTAGCCGAGGTC
ATAAAGGAGCTCCACCTCCCCCCAAACGTCGAATTGCTAGAACAACTCGCAGACAGCGCGCTCAAGATCATCGTTGGGTTTCTCCAACTCGTTT
CGTATAATCTGCATGCCTCCAAGAAGAGCAGCGGGAACGCCGTGGCCGTCGTCATGTCTCTCAGGGAAATATCCCTGCAGGGGGCGCTCAAAGT
CTGCATGGGCGAATGCAGGAGGAATGTCGAGACGTACCGCACTGTCGAGCGCGCGATTCTCGTTGGTCTCTCCGGAGAGGCGCCCCCGTCAATG
CCGCCACGCAGGCCCAGGCCGCTTGCAGGACATACACGGGCTTCGAGTTCGATCTGGTCGTGGATTCCTTTCGTAGCTCCGCAGGAGGCTCCGG
CGCCGCCTGCCCCCGAGCCGGCTCCTCTTCTCCCTTTACTAGAGGTCGAGGAGTGGGACGATGAGGTCAAGGACGACGTAAGATCATATGTGAA
CGGGCTGAGAGATTGCGTCGTGGGGTATATCCACTGGTTATATGAGACGGAGATATATTTTGGACAGCTTGGGGACGAGGTCAGGGCTACTGGA
TGGACATTCCTACCCGTAGTTTCATCGTAG

```
//
>PoSTS_11
ATGGCCTCGCATTACCGTTTACCCAACCTCCTCGCTCTCTTCAACACAGACGGTGCTCGTGTCAACCCTCGTTTCCACGAACTTGATTCGCAGT
TCAATCAATGGCTGGACACTCTCACTCTCGACGCGGGCTTTGTCGAGACGTTGAAGCAGATCCAGATGCCGGTGTTGATATCCCATGCATATCC
AGAAGCATCCTTGGGTCAGCTTAGGACATGCCTGGACTACCTCACTCTGGCTTTCATCTTCGAGGAGATCACGGAGAACACATCCAGCTCGCAG
AGCCAGCGATGGGCAGACCTTTATATGGGCATGTACCGGGGGACAGCGTCTGTCCTTTCGAACATCGTGCCTGAAGAAACCGACCATCCGCTAT
TTCCTGTCATGACAAGCCTAGCACACAACGTCATGTCATCATTGGAACCTGTCTTTCACGAATCGTTCATCACGGAGAACCTTGCTTCTGTCCA
GGCCATCGTCCAAGAAGCCATTGATCGTGAAGTCGATGATGCCCCCTCTCGCCAGCCGCGGACGCTCGAAGCCTATTATATCAACCGCCTGGCT
ACTGTCGGTCTGATGCCATTCCTCATTCTCGCACAATGGACCGGTGGGATTCGGTTGCCAGCTTTCATCCAAGATTCGCATTCTGTCCAGACGA
TGTCCCAAGCGGCCGTTGAAATGGTGTTTTTAGCCAATGATATTTACTCTTATAAGAAGGAGAAAATGGCTGGGGCGACACAAAATAATGTCAT
CACCGTCATAATTGAAGACCCATCCACAAGCATCTGCGAGGGGAACCTCCAAGGAAGCTTCGATTACTCAGAGAGGCTGTTTCAGGATGCCCTG
AGCCGCTTTTATACTCATAGGGAGATGCTTCTGGAAAATGTATCGGACCAGCAAAACTACACCGCCGACATAAACAAATTCAGCCGAGCGATGA
TGGACTGTGTTGTTGGAAACATCAAATGGAGTATGATTTGTAGGCGATATGCTGTCTTCGAATGCGAGCAGGCGAGGAATAATGAGGTGGTGAA
AATATGA
//
>PoSTS_16
ATGCCTGTCAATATCCGTGACATTGTGGCTTTACATCTTCGCCGTTGCAACATCCCTTATGAAGTACCTGTTCCCGATGTAAAGCTCCAGGCAG
CATGCCTCAATTACGCCAAGAACCACGGGTACGAAATTGGCGGCAAGAAGACTCTGGGGCCATGCATACCTGGAGGAGTCGTGATGGCCTCGAA
CGCGTTCGGCCATATTGCCGACCTCCCGACTCGCGTCATCATTGGCATATACACGGCGTTCATGATCTACCTGGACGATATCTCCTCCAGCGAC
ATCGAGGCCGTTGCACAGTTTAACCAAAGATTTTACCGCGCAGAGCCTCAGCTCGATAAAGTGCTTGACGACTTCGCGCAGCTTCTTCGAGACT
TTCCCAACTATTTTTGCACGGCTGGGTCGGATATGATCGTCACCTCAACACTGAATTTTGTGACGTCCCTTTTTATGGACGTGGAGACAGAAGG
CATGGATGTTGATGGAAATGCAAAGCGATACCCCAGGTTTGCACGGATTTTGAACGGGGCTGCCCTCGCGTATACTCTCCTAGTGTTCCCGAAG
TCGCTGCCAATATCAAGTTATATTCAAGCGGTCCCGGAGATAAACGTCTACGTGGAGAACACTAACGACGTTCTCTCATTTTACAAAGAACAGA
TGGCGGGAGACGATATCAACTATGCCAGCCTGCTTTCTCACTCCTACAGTGTGACGAAGTACGAAGCACTCATCCACCTCAGTAATATCGCCGT
TGATGCGAACGAACGAATTCTCCGCCTCCTGGAACCGGTTCCCGAGGCCTTGGAGGCTTACACACGGTTCAGGAATGGCTACGTTAGATTCCAT
ACTGGTCTCGGTGGTAGGTATAAACTAGCAGAACTGAAGTTGACGAACATAAGTGATTACCAGGATAGACTTGGAGCACATGTCAATCAAACGG
CGTGTAAAGTTTGTCAACGTAATTTGTGTAATTAG
//
>TvSTS_01
ATGGCCGTCATCGCCGCCGCACCTCGTCCCAAGAGGATCTTCTTCCCGGACCTCATCGCCCACTGTCCTTACCCCCTGCGCTACAACCCGCACT
GCGAGGCCGCATCCGCCGAGTCCAAGGTGTGGCTCATGTCCGGGTGCCGGCTGAGCAAAAAGAAGCGTGCCGCCTTCCATGGGCTCAAGGGCGG
GCTGCTCACGGCGATGGTGTACCCGGAGGCAGACTACGACCAGTTCCGCGTGTGCTGCGACTGGATCAACTACCTCTTCCACCTCGACAACATC
TGCGACGAGATGGACGACCGCACGACCGTCAGCACCGCCGGCGTGATCATCGGCGCGCTGCGCGACCCGCACGGCTTCCGTCCCGCGTCCGCAG
TAGGAAGGCTGACGCAGAGCTTCTGGCGCCGGATGTCTGCGACGGCGTCGCCCGGCGCCCAGCGGCGGTTCATCGAGACGTTCGAGCTGTTCTT
CCGCGCGGTCGCCCAGCAGGCGCGCGACCGCGCGTCGGGCAACATCCCCGACCTCGAGTCGTACATCGCGATGCGGCGCGACACGAGCGGGTGC
AAGCCGTGCTGGGCCCTCATCGAGTACGCGAACGACCTGGACTTGCCCGACTGGGTCATGGACCACCCATGTGTGCGAGGCTTGGAAGAAGCCG
CGAACGATCTGGTCACGTGGTCGAACGACATCTTCTCCTACAACGTGGAGCAGTCGAACGGCGACACGCACAACATGATCGTCGTCGTGCAGAC
GCAAGAGCAGCTCGACCTGCAGTCCGCCGTCGACTACGTCGGCGACCTCTGCCTCGGGTGCGTCGACCGCTTCGAGACGCTGCGCGCCGCGCTG
CCCTCGTGGGGCCCGCAGATCGACGACCAGCTCGCGGTGTACGTCCAGGGCCTCGGCGACTGGATGATCGGCAACCTCGTCTGGTCGTTCGAGA
CCGAGCGGTACTTTGGGCGCTCGGGCAGGAAGGTGCGCCGCGCGCTCGCCGTCGCCCTTCTCCCGCGGCGCAAGTAG
//
>TvSTS_04
ATGCCCGTCATCCTCGCTTTCCCTGACTTCATCTCGCCGGTCCCGTACCCCCTCCGCTGCCATGCACAGGAACGCGAGGTCTCGCGCGAGTCCG
AGGAGTGGCTTCTCAGCATGGCGAACTTCTCGGAGAAGCAGCGCGCCAAGTTCCTCACTCTGAACGCCGGTCTCCTCAGCGGCATGTGCTACAT
CGACTGCTCGTTCGACGAGCTCCGCGTCTGCACCGACTTCATGAACTTCCTCTTCACCCTCGACGACTGGACAGACGAGTTTGACGTGACCGGG
ACGCGCGGCCTCGCGGAGTGTGTGATGAACACGCTTTACTGGCCGCATACGTACTCGTCGGATACCGCAGCGCACAGGCTCACCAAGTCGTTCT
GGGTGCGCATGCAAGAGACCGCAGGCCCTGGTTGCCAACAGCGCCTCATGTCCACGCTCGACACCTACTTCCAAGCAATCATGCAGCAGGCGGA
CGACCGCGGCTCCGAGAACATCCCCGAGCTGGAGGAGTACATCCTGCTCCGCCGCGACACAAGCGGCTGCAAAACGGGCTTCGCGTTCATTGAG
TACGCGGCGCGCATCGACCTCCCGGACGACGTCGTCGACCACCCGATCATCAAGGCGCTCGCTGACGCGACGAACGACCTCGTGTCATGGGCAA
ACGACGTGCTCTCATACAATGCGGAGCAAGCCCGCGGCGATACGCACAACCTTGTCTGCGTGCTCATGCACATGAACGGCGTCGACCGGCAAGA
CGCGATTGAGCTCGCCGGGGAGCTCTGGACGAAGACGCTCGAGTGGTTCTTCGAGTGCCGCAAGGTCGTCCCGTCCTGGGGCGCCGAGATCGAC
CGGCAGGTCGCGCTGTACATCCAGGGGCTCGAGGACTGGATCATCGCGAACGCGGAGTGGAGCTTCGAGACGGAGCGCTACTTCGGCAAGGACG
GCCACGCCGTGAAGAAGTCGCGCAAGGTCGCGCTCCTCCCGCAGCGCGTCCGCGCATGA
//
>TvSTS_05
ATGTCTTCCCCGTCTTCGTTCGTCCTGCCCGACCTCCACGCCGTGACCCCCTTCAAGGGCTCCTTCAACCCCCACTACCCCGAGGCGGCCGCCG
AGTCCTCAGAATGGGTCAACAGCTACAAGGTCCTCTCCGACAAAAAGCGCGCCTTCTTCCTCCAGGGCGGCAGCGAGCTCCTCTGCGCACACGC
CTACCCCTACGCCGGGTACCAGCAGTTCCGCACCACCTGCGACTTCGTCAACCTGCTCTTCACGGTCGACGAGATCAGTGACGACCAGAACGGC
AAGGGCGCGTACGAGACCGGCCTCACCTTCTACAACGCGATGAGCAACCCCGCGTACGACGACGGCACCGTCCTGTGCAAGATGACGAAGGAGT
TTACCGCGCGCCTCCTTGAGCACTGCGGCCCTCAGACGTACCGTCGGTTCATCAAGCACTGCAAGGACTACATCGAGGCCGTCGCGGTAGAGGC
CGACCTCCGCGAGCGCGGAGAGGTGCTCGACCTCGAGGCGTACCAGACGCTCCGCCGCGAGAACAGCGCCGTGCGCTTCTGCTTCGGTCTCGCC
GGTTATGCCCTTGGCATCGACCTGCCCGACGAGGTCGTCGAGCACCCGGCCTTCATGGCCATGCACCTCTCCACCGTCGACATGGTCTGCTGGT
CTAACGACTTGTACTCCTACAACATGGAGCAGGCGATGGGCCACACCGGCAACAACGTCATCACCGTGCTCATGCAGCACAAGGGCCTCGACCT
GCAGGGCGCTGCTGACTACACCGGGGTGCACTTCAAGGGCCTCATTGACACGTTCCTCGACGCGAAGCGCAGCCTGCCCAGCTGGGGCCCGAAG
CTCGACGGCGAGGTCGCGCAGTACGCGATGGCGATGGAGACCTGGGTGATCGGGAACCTCAACTGGAGCTTCGAGACGCAGCGCTACTTCGGGC
```

ACGCGCGCCACGAGATCAAGCGCACCCGCGTCGTGCAGCTGTACCCCCGCCGCATCGTGGAGGAGTCATCCGACGAGGAGGACAACTAG
//
>TvSTS_06
ATGTCTGCCCAACAGTTCACCCTCCCCGATCTCCTCGCCGTCTGCCCTCTTAAGGACGCGACCAACCCACACTACGCCCAGGCTGCCGCCGAAT
CAACCGCTTGGGTGAAGAGCTACAACATCTTCGATGCGCGCAAGCTCGCCTTCCTCCTGCAGGGCAGCAGCGAGCTGCTCGTCTCGCACGCATA
CCCCTACGCGCCATACGAGCAGTTCCGCACTTGCTGCGACTTCGTCAACCTGCTCTTCGTCGTCGACGAGGTCAGCGACGACCAGAACGGCAAA
GATGCCCGCCGGACCGGCGAGGTGTACCTGAACGTCATGCGCTACCCGGACTGGGATGACGGCTCGGCCCTTGCGAAAATGACTCGAGAGTTCA
AACAGCGGTTGCTCGCCTTCGCCGGCCCCAACAGCTACCGCCGGTTCCTCATGCACTGCGACGACTACGTGAACGCCGTCGCACGCGAGGCGGA
GTACAGGGAGCGCGGAGAGGTGCTCGACCTCGATGCGTTCCAGACGCTCCGACGCGAGAACAGCGCTATCCGCCTGTGCTTCGGCCTGTTTGAG
TTTGCGCTCGGCATAGACTTGCCCGACTTCGTCTTCCAGGACCCCCACTTCATGACCCTGTACTGGGCCGCCGCCGACATGGTCTGCTGGTCAA
ACGACGTCTACTCGTACAACATGGAACAGGCGAAGGGCCACACCGGCAACAACATCGTCACCGTGCTCATGCGGCAGAAAAGCATCGGTCTCCA
GGAGGCAGCAGACCTCGTCGGCGCGCATTTCGCCGCGCTGATGGGCCGATTTGTGGAGACCAAGAAGCAGCTGCCATCATTCGGCGCGGCCGCA
CTGGATGATGCGGTGGCAAAGTACGTCGCGGCGATGGAGCACTGGGTCATCGGCAACCTCGAGTGGAGCTTCGAGTCGCAGCGGTACTTTGGCG
CAGAGCACACGCGCGTGAAGGCGACCCGCGTGGTCGTGCTGAGCCCCGCGGACGAAAACTGA
//
>TvSTS_07
ATGTCTCGCTCTCTTCGTCTTCCCGATACTCTCTCGCGGTGGCCCTACCCGCGGCGCATTAACCCCGCTTACGAGGAGGTTTCTGCTGAGTCTG
CGGCATGGCTGAGGTCGTTCCACGCGTTCTCCGACCAGGCACAGGTCGCCTTCGACAAGTGCAAGTTCGGTCTTTTGGCCTCGCTGACGTACCC
AAACGTCGACAAAGATCACCTCCGCGCCGCCTGCGACCTCATGAACGTCTTCTTCGTGTTCGACGAGCAGACGGACATCGCGGACACGACCCGT
ACCCGCGAGCTCGCGGACATCGTTATCGACGCCGTGCGCCATCCGGATCGCCCCCGTCCAGAAGGGGAGCCCATCGTCGGAGAAATAACGCGGC
AATTCTGGGCCCACGCGTGCGTAAACTCTACCGCGTCTGGGCGGGCGCGGTTCGAACAGGAGTGGACGCGCTATGTCGAGTCCGTCGTCGGGCA
GGCGGAGGACCGCGATGCGGGCCGCCTTCGCACGACTGAGGAGTACCTCGAGTTGCGGCGGTTCACGATTGGTGCCGACCCGAGCTACGCGCTC
GCGATGGCCCGAGTGGACCTGCCACTCGCCGTGTCCGAACTTCCTGTCTTCCGGAAGCTCCGCGGGTGCATCACGGATATGCTTATATTTGATA
ATGACCTGCTCTCGTACCGCAAGGAGTACGCCGCGGGGGACGACATGCACAACATCATCACGCTAGTCATGAACGAAAAGCAGATTGACGTGGA
TGCCGCGGTCGAATGGCTCGCCGCGGAGCACGCCAAGCGGGTGGACGAGTTCTTCGTCCTGTGGCCACAGGCGTCGGCGATGTCCTTCGGCTCG
GACGAGCTCAATCAGGCGGTGGCAACCTACTTGGACCATCTCGTCAACTGGCCCCGCGGAGATGAGTGCTGGAGCTTCGAGAGCGGGAGGTACT
TCGGGAAAGACGGCGCCCGCGTCAAGAAAGAGAGGGTCATCGAGCTCAAGACTAGAGATTAA
//
>TvSTS_09
ATGCCTCAGCAGTATATCCACATTCCCGACCTAATGGCCACCTGGCCATGGCCTGACCGGATCCACCCTCTGCATGAGGAAGTCGACGCCGAGT
CGAGCGCGTGGCTCAAGTCGTTCGCCCCCTTCACGCCCCAGTCGCAACACGCATTCGACAAGTGTAATGCTGGGCTGCTCTGTGCCCATGTATA
TCCCGACATATCGCGAAAAGCCCTGCGTACGAGCATGGACCTCATCAACATATTGTTCGTGGTCGACGAATATACGGACGTTGAGTCTGCGCCC
GCCGTGAGAGAGATAGCCGACATGCTCGTCGACGCGGTGTACAACCCGCAAAAGCCTGGTCAGGCGGGGGAGCTAGTTTTGGGGAAAATGATGA
GAGAATGGTCGCGACGTTGTCTCCCGGCTGCCACCCCGGAAGCAGTCTCGCATTTTCGCAAGTCGTTCACCGATTACCTCGAGGCCGTTGTCTC
GGAGGCCGAAGGACGATGCACCGGCACCATTCGCTCGATCGACAGCTACATGAAAAACCGCCGCGAAAACAATGGTGTTCGCCCATCCCTCTTT
CCATGCGAGCTGCATTTGAGCATCCCGGATGAGGTGTTCTACCACCCGCATGTTGTAGATCTGCAGACATGCATCGTCGACATGATTACCACGG
TTAACGATATCATATCGTACAACAGAGAACAGGCCACCCACATCGACGACTGTAACCTGCTCACGGTCGTCATGCGGGAGCTAGGCACAAACTT
CGATGGTGCCATGGCCTGGATCGTCGATTATCACCACGACGTCGCTACGCGATTCATCGAGGGACTCAAAAACATCCCCTCCTTTGGGCCCGAA
GCCGACGCTCGACTCCAGGAGTACCTGCAAAGCATAGCGAGCTGGCCGGGCGGTGCAGACAGCTGGAGCTTCGAGTCCGCGAGGTACTTTGGGG
AGCGGGGCGCGGAGTACCAGAAGTCGCGCCGGGTCCCTCTTCTGCAGAAGAGTAAGCGGTACCCGCAATGCCGTCGCGAAAAGGTCCATGTCCA
CCTTATAGAGCACCTTGCCCAGCCGACCTCATGA
//
>TvSTS_10
ATGGCGACCTGGCCATGGCCCCGACGGATCCACCCGCTATACGAAGAAGTCGCCGCCGAGTCGAGTGCATGGCTCAAGTCGTTCGCCCCGTTCA
CGCCCGAGTCGCAGCACGCCTTCGACAAGTGCAACTTCGGGCTCCTGGCTGCCCTTGCATACCCCGATGCATCGCGAGAAACCTTGCGCACCAG
CATAGACCTCATGGACCTGTTCTTCGTGATCGACGAATATACGGACCATGAGCTGGCGCCAGCAGTGAAGGAGATGGTCGGCATGCTCCTCGAC
GCGGTACACAACCCGCAGAAGCCCCGTCCGGCGGGTGAGCCTATTCTGAGTGAGATGATGAGAGAGTGGTCAGAGCGCTGTCTCCCAACCGCCA
CCCCCGAGGCAGCCTCCCATTTCCTCAAATCGTTCACCGACTACCTCGAGGCGGTTGTCGCGCAGGCCGACGAACGATGCAGCGGTGCTGTCCG
CTCGATCGACAGCTTCATCAAGATCCGTCGCGAGAACAACGGCGGCCGCCCCTCCCTCTTTCCAAGCGAGCTGCACTTGAGCATCCCGGACGAG
GTGTTTTACCACCCATACGTCGTCGATATGCAGGCGTGCATCATCGACATGATCACCACGATCAATGACATCCTGTCGTACAACAGAGAACAGG
CCACTCATACCGACGACTACAACCTGGTCACGGTCGTCATGCGGGAGCTTGGCATGGACCTCGACGGCGCCATTGAGTGGGTCGTAGATTTCCA
TCACGGCATCGCAGCTCGGTTCATCGAGGGACTTGAGCACCTCCCCTCCTTTGGGCCCGGGGTCGACGCGCAGCTCCAGGAATACCTGCAGAAC
ATAGCCAACTGGCCGCGCTGCGTGGACAGCTGGAGCTTCGAGGCGGGGAGGTACTTTGGGGAGCGAGGTGTAGAGTACCAGAGGACGCGCCAGG
TGCCTCTTCTACCAAAGAGTAAGCGAGACGTGCTATACCATCGCGAAGAGGTCCAGGTCCACCTCATAGAGTACCTTGCCCAGCCGACCCCAGC
GTAG
//
>TvSTS_12
ATGAGCGTCGAGCAGCTCCGCCCTTTCCCGACGCACTTCCGTCTGAAGGACCTAACTGCGATCACTTCCCCCGTGTTCGAGTTCAAGACGAATC
CGCACCAGGAAGCAGCGGCGCAAGCCACTAACGTGTGGTTCGAGAGGCGTAATGTCTACCATGGGCTCAAGAAGCAGAAGTTCTTGTCCCATCG
GTTTGACTCCTACGCGGGGATGAGCTTCCCGGATGCTGACGCTGCCCACCTCGAGACGTGCATCGCGTTCTTTTTCTGGGCGTTCTCGTTTGAC
GATTTATCCGACGAGGGTGCACTGCAGTCCAAGCCCGACGCAGTCCAGGTCGGTGTGGACATCTCCATGGAGGTCCTCAAGAACCCCGCCGCGC
CCGCTCCGAATTTCCCCTACGCCGCCATGTTGCACGACATTTGGCGACGTTTCAGATCTACCGCCAGCCCTGGCGCCTGTAACAGGTTCTACCG
GGCCGTCGAGAGCTGGATGAACTCGCAAGTCGAGCAGGCTCGGAATCGCTCCACAGATGAGATTCCTTCCGTCGAGGACTTTATCATCCTTCGC
CGAAGAACCATTGGTGGTCCTATTGTGGAAGCAATGGTCGAATACTCATTGAATCTCCAAATCCCCGAGTACGTCTGGGACCACCCCGTGCTAC
AGGAGATGTCGAAGGCAGTGATCGACATCATGACCTGGCCCAACGACCTGTGCTCGTTTAACAAAGAGCAGGCCGATGGGGATTTCCAGAACCT

CGTGTTCTGTATCATGATCGAGCGCGATGTCGATCTCCAGAGGGCAGTCGACATCCTAACCGACATGCTGGCGCAGCGCGTTGCGGACTACGTG
AGGTACAGGGACCAGCTTCCTAGCTTCGGACCCGAGGTAGATGCCGAGCTCGCTCGTTACAACAAGGCCATGGAGCAGTACACGCAAGGCACCG
TCGTCTGGTACTACTACAGTCCTCGTTACTTCCGAGGACAGGACGTCTCTGGGAAGACAGAGATTGTTGTGCCCGTCTACGAGCGCGCCGCGCC
TGTCGAAGAGGCCGCACCCGCGCCGCATGTCTCCACCACCGACTCCGAGAAGGGCGCGGTGTACGCCTCGTTCCGCTTCCCCTTCTCCGCGTTC
CCCCATCTATCGTTATTGCTTCTCTCGCTGTTCGCCTGCTTGCTGTACGTCAACCTTCCGTCGCTCGGCCTCCGTCTCGCTCTCCCGTGA
//
>TvSTS_13
ATGGGGAGCATCCGCATCCCGAATTTCCCAGCGTTCTGCAATCCGTACCTCGAACTCCGGAGCAATCCCCACTGCCACGCCATCTCGCTCTCGG
CCGAGAAGTGGGCGCTGTCGACCCCGCTCTTCCTCGACGCGGAGGAGGAAGCACGTCTGCCAGGCGCACGCCTCGGGCTGCTCGCGGCGCTCTG
CTTCCCCACATGCGACGCGAGCCAGCTCTTCGCGATCACCAAGTTCCTCATCGTGATCATGCACTGGGTGGACCGGCCGCGGTCGCTGTACGCG
GACGAGGAGGCGTTTGACCCGGTATGGGCCAAGTACTTCCGCCCGACGACGCGCCGGGAATGGCAGAAGCGTTTCCAACGGCACCTAGCCGCCT
TCCGGCTCGGGCAAGCACTCACGGCACAAGATGCGGCGCAGGGCATCGTCCCCGATCTGGAGTCGTACATCGGGCTTCGGCGAGACGCGTGCGG
GGTCAGAATGCTGCTCGACCTGGTTCTTTACGCCGGAGGGTTGAACGTCCCACCATACGTGTACGAGCACCCTGTCCTGCGAAGGTTGCGGCAA
GATGCTGCGGATATCATCGCGTGGTCCACGGACGTCGCGGCGTACGCGCGGAGGCCTCATAGCAGCAACATCATCGCAGTGCTCATGCATGAAC
GACGATATACGGCACAGGCGGCCGCGCACTTTGCCGGGAACCTCGTCAAAGACACCATTACGAACTTCGTCACGAACGAGATGTCGCTGCCGGC
GTTCGGTGACCGAGACGCGGATGTCCGGGCCTACGTCCAGGGCCTGCGCGATTGCGTCGTCGGCACCTTGCACTGGCTGTACGAGACCGACCGG
TATTTCGGGGAGACGGCGGAGGACGTGCGCTCGTCCGGCTGGGTGTTCATGAACGCGTGA
//
>TvSTS_14
ATGCCCTACATTCAAGATGCATGCTCCTCGCCTCTGCCGTTGTGTGCGAGCGTGGACGCGATAACAGAGATCAAAGACATCCTGAAGGACTTCC
TGCGGCGTCTCAACTACCGCTCACCCCACACCCCCGCGAACGCGAAGCTCAGGGCCGAAGTCACCGCCGAGATCGCCTCATGGAACGCCGATCT
CAGCCCGTCCTTCAAGCACGGCCTCGCAGAAACCTGCTACACCATCGCCGAGTCCGCCTACGCACACACCAGCTACGAACACCAGCGTATCATC
GCACTCTACACGACCTACCTCACATATGTCGACGACCTCGGTGGGCGGGACCTCGACGCGCTCGGAGAGTTCGGACGGCGGCTGCTCGCGCGCG
AGGCGCTGGGGGACTCTGCGCTGGACCGCCTCGTCACGAACCTGCAGGACATGTACGCGTACTATCCCCGCCTGAGCGCGCACTCGATTGCGGT
CTCGACGCTCGACTTCTTCGTCGGGTCGTACGTTGAGGCTACGGGGAAAGAGATGGCGGTCGCGCCGGGGGCGACGAAGTACCCCGGATACATG
CGCATGAAGACGGGCATCGGCGCGGCATATGCGCTGTTCAACTTCGTGAAGGACTGGCGGGATCCGGCGGACCATTTCTTTTTGCAGCTCATCC
CCGAGATCGAATTCTACACAGATACTATCAATGACATACTTTCATTCTACAAAGAATCGTTGGCCGGAGAGACCGACAATCTCATCCACCTCCG
CGCCGCGGCCGAGCAGAAGGATCCGCTCTCTGTGCTGCGCGATGTCGTGGAGGAGACGCTGGAGAGCATCCGCAAGGTGGAGGTGCTGACCGCC
GCGGACCCGCAGCTGGCGAAGATCTGCCGCAGCTACGTCGTTGGGTATGTCGAGTTCCACTTCCGAGCGAAGAGGTATCGACTGGAGGATTTGG
AGATGGAGTATTGA
//
>TvSTS_16
ATGGTCGCAGAAGTACTCCCTTCCCCCGCCAATCCGTTCCTTGTCTACGCCAATGGTGTCTCTCCAATCTTACCGACATCCCTCTCCCATATAG
ACGGTCATGTCTATCGATCGGGAAGCAACGATAGTAATGGCCTTTCAAGGCTCCTAGTCACTGGTCCGCAAGGATTACCACCCGCAATCACACC
CGAAGCCATTCAGGAAATGAAGGCAATTATGCAAGATTTCCTCCAGCAGTGCAACTACGAGGACCCAAGTACACCCAAAGACATCGAGCTGCGG
GAGAGAATGGCGTTGGAGATGGCGGCGTGGAGCCTCGACCTTCCTGCCACATTCATCGAGCAGGTGCACGAAACGAGCTGTCATTTTATCGAGA
CGGCCTACGTGCACACGACCCTGGAGCATCGCTTCTTCGTCGCCCGGTACTGCGCATACTTCCTCTACGCCGACGACCTCCCCGGCCGCTGTCT
GGACGCGCTCATACAGTTCCCGCGCCGCTTCGCAAACAGAGAGCAGCAGCTGGACCCCATCCTCAACCGCCTCGCGGAGATGATCAGAGGCGCG
CACGAGCTCTGGACGGACGTCGGGGCGAGCGCGATCATTGCGGGAACGCTCGACGCGCTTACCGCCTTCTACATCGAGTACACCACGTGCGGGC
TGGCAGTGAAGCCTCAAGCGGTGCGCTACCCGTACTACCTCCGCCTGAAGTCGGGCATCGACCCGCCATTCGTAGCGTTCATATTCATGCGCGG
ATGGCGGGACACGGCCGAGTCGTACGTGCAGATGCTTCCTGAGATTGAGTACTGGATCGGGGCTACAAATGATTTGTTGTCATTCTACAAGGAA
GAGCTGGCGCAGGAAACGAACAACTACATCCACATCCGCGCCGCGATGGAGCAGACAACGGGGCTCGTGATCCTTCGCAAGCTCGCCGATGAGA
TCCTGGACACTACGCGGCGGATCGAGGGCCTGGCCGCGGAAGATGCGGAGCTCGCTGCCTTGTGGCACGGCTACAAACAGGGCTTCCTCGAGTT
CCAGATCAAGGCGCCCCGGTATCGGCTCTCGGACCTGGGGTTGACGGAATGA
//

Amino acid sequences of isolated STSs

>AbSTS_01
MAFAVASSSPKSFILPDLVSHCDFKLRISRHRKQVTVETKKWLFQGDSLDANKRKAFHGLNAGLLTAMTYPNAACPQLRVCNDFLTFLFHLDNL
SDDMDNRGTHNIADVVLNSLYFPYSYPSTARIGRMTKHLYKRVIPTSSPGTQQRFIETMDFFFQSVTQQALDRANGVIPDLDSYIALRRDTSGC
KPCWALIEYANNLKIPDEVMDHPTILALGEAANDLVTWSNDIFSYNVEQSKGDTHNMIPVVMYQKDLSLQEAVDFVGQMCKSSIDRFKAERDNL
PSWGPHIDRQVNVYVQGLADWIVGSLHWSFESTRYFGSIGRKIKSTRVVELLPLRQEARARAATVTLRPTLSPITVPPRAPSPATPTESEPVTP
SSSVHSIPVPKSADSEESIPHSPWSVQRSQDVIVSPIPNSPKPTLGRIQTESLFVSETQEQTPESGVLVQYPEILSPAMANPMVNQRIVRNEPL
GLYSLTIWLFYTLIYAPTMTLLRVFLNQDVHPHPLCHYGYL*
//
>AbSTS_05
MPRPQQFILPDLLSSCPLEDGLNPHYREAAAESRAWINSFNIFSNRKRADFIQGLNELLCSHVYCYAGYEEFRTTCDFVNVLFVVDEISDEQSG
KDARATGLSYAESMRNADWDDNSVVAKITKEFRARLIRRAGHNNFRRFVASSDAYTRCVGREAELREAGEVLSLEEYMPLRRNNSAVLLCFDLV
EYILGVDLPESIYQNATFLKAYWAACDHVCWCNDVYSYNVEQSKGHTGNNVVTVLMNDRQIGLQEACDYIGDRCRQFMNDYLAARDELRATVGG
DASRFIDALGYWIIGNMEWSFESPRYFGHEHDEIKRTLTLTLKPSEVPEEVDSDSDCE*
//
>AbSTS_07
MRAIEQLSNQPLSHKALEDGVRDVISDFLLKTNQTPKAPEQPDALVFERECWAEADRRGYDMHYLSKHLPVGILVSNAAYFYQSFELRLYIAYY
TGLLLCVDDNFDIQSDGIARFMERYQRGECHPTDVLNNLADLLAETSTFFDPVATNLIMCATFSFMNAMVIENITAGTKIPSQAKRYPDYLRRF
SGISKAYATFIFRPSVSAAQYIHALPELEDIINYVNDITSFYKEELAGEDENTVSLLAKLNNRSKLDQLRVLSDSVAESHRRTLAILKGRDDAE
GDYLLFWSGYIPFHVSAKRYRLMELGIH*
//
>AbSTS_09
MGAVGQLSNQPLSREALEDGVRDVISDFLLKTNQTPKAPEQPDAPVFERECWAEADRRGYDMHYLSKHLPVGILISNATFFYHSFELKLYVAYY
SGILLCVDDNYDTQSRVDGIARFMERYQRGERHPTDILNNLADLLAETATFFDPVATNLIMCATFSFMNAMVIENITAGMKIPSQAKRYPDCLR
QFTGISKAYAAFIFRPSVSAAQYIHALPELEDIINYVNDIASFYKEELAGEDENAVSLLARLNDRSKLDQLRVLSDSVAESHRRILAILKGRDD
AERDYLLFWSGYIPFHLSTTRYRLMELGFN*
//
>AvSTS_01
MQSPTQASYRLPETLAGWPWPRMINPHYEEVKAESRAWFHSFRAFGPKSQDAFDKCDFWLLASLSYPFTDKARLRTGCDLMMLFFVFDEYTDLS
HGNDVRVYADMVMDALRNPHKPRPAGEILLGEVARQFWELAVKTTTPTAQKRFVDAFTRYTDAVVAEARDRDETHVRSIDEYFNIRRYTIGAEP
SYVPMELAMDIPDEVFFHPTVVKLTQLVTDVILLDNDLCSYNKEQANGEELHNILTIVMAELKVDLNGALDWLERRHAELNEAIIETWNSLPVW
DEDIRDDVDEYLLGVVGWVRSNDSWNFESQRYFGTDGLEIQKHRMVTMRPRKAGLGCPVEGDPLQELMV*
//
>AvSTS_03
MPEKFYIPNCLENWKWPRALNPYYEEVKAESAAWARSFGAFSPKAQHAYDRCDFNKLACLAYPLLDKAGARIGCDLMNMFFVYDEYSDVAPADE
VQVMADIIMDALRNPHKPRPEGEWVGGEVTRQFWELAIKTASPQSQKRFIKTFGTYTQSVVQQAADRDHHYVRTVQEYLEVRRDTIGAKPSFAI
LEVGMDIPDEAIEHPIIQELTILSIDMILLGNDIASYNLEQARGDDNHNIVTIVMHQEKTDIKGAMDWVVQYHKTLEDRFMELYAQVPSLDFGE
RVNKELAVYVDGLGNWVRASDQWGFESERYFGKKAPEIQKTRWVTLMPKERTDEIGPQIVDGSML*
//
>AvSTS_04
MQSPTQDSYRLPETLAGWPWPRMINPHYEEVKAESRAWFHSFRAFGPKSQDAFDKCDFWLLASLSYPFTDKARLRTGCDLMMLFFVFDEYTDLS
HGNDVRVYADMVMDALRNPHKPRPAGEILLGEVARQFWELAVKTTTPTAQKRFVDAFTRYTDAVVAEARDRDETHVRSIDEYFNIRRYTIGAEP
SYVPMELAMDIPDEVFFHPTVVKLTQLVTDVILLDNDLCSYNREQANGEELHNILTIVMAELKVDLNGALDWLERRHAELNEAIIETWNSLPVW
DEDIRDDVDEYLLGVVGWVRSNDSWNFESQRYFGTDGLEIQKHRMVTMRPRKAGLGCPVEGDLLQELMV*
//
>AvSTS_06
MSTQIQFRLPDPVEICHWPLPRMLNAHYAEVKAECVAWIHSFNALSPKAQKAFDKCDFSLLASLIYPSLDRQHLRTGCDLMMLFFVFDEFTDKE
DGKGVRKYVDIVVDAIEHPDRPRPAGEHVLGEITRQFWERAIQTASASSQRHFVKTFKEYAEAVIEEASDRVSDRVRSIDDYLALRRLTAGPYP
GFFPCEIRVDLPDDVFYHHSVANLTRLVAESVVVTNDTYSYNIEQAAGHQGHNIVTVVMREKHLSLHQALEWVGNYHAGILTEFLESRKKLPSF
GEELDAQVADYVEGLAHGVRGLDNWCFESGRYFGSKGLEVQKHRTVGLLPKVVQHDVATPMMALPVSDLDKMEVGESDLEKHQSSGWRYFDCFS
CLSGRWMYL*
//
>AvSTS_07
MQSQIPLTHFRLPDPAANWPWPRTLHVNYEEIKAEADAWLHSFNALSSKAQRAFDKCDFSLLGCLLYPHLDKERARTGCELMILFFIFDEFTDQ
EDGPGVRRYVDIVLDALRNPHVPRPVGEHVLGEITRSFWERAIKTATAASQRHFIQTFSEYAEAVILEAADRASERVRGIEDYLALRRLTAGPY
PGFLPCELRIDLPEAVYNHPALANMRRLVAESIVLTNDTYSYNIEQAAGHDGHNIVTVAMRELHLALPAALEWVGAYHANILAEFVECRGELPS
FAAEDAQIVEYVEGLAMGVRGLDAWCFESARYFGTRGREIQRERVVGLLPKTVIVGGLATPMMAGPAVEGNSVVV*
//
>AvSTS_09
MLTSPGHFYLPDLLALSTPFHSSTNPHYKEAAAESRAWINSYNVFTDRKRAFFVQGCNELLVSHTYPHAGYAQFRTICDFVNLLFVVDEVSDDQ
SGADARKTGEVYLNAMRDPEWDDGSPLAKMTKEFRARLLGSCGPRSFARFLRHSEDYINCVALEAEYRERGEVLDMEAFKHLRRENSAIRLCFG
LFEFSLGIDLPDEVFQETTFSSIYWAAADMVCWANDVYSYNMEQAKGHTGNNIVTVLMKAKGFDLQAASDYIGIYYAELMNRYIADKARLPSFG

PSIDTDVQRYVTAMENWPIGNLVWSFETNRYFGPRHAEIKRTRLVILKPREEDT*
//
>AvSTS_11
MSSPTHFVLPDLLSLSTPFQDATNPHWKRVAAESRNWVNSYKVFSDRRRAFFFQGQSELLTSHCYPYADAEEYRMCCDFINLLFTIDEISDEQD
HAGALLTGNTYLHVLRDPSWTDGSKLAKMTADFRSRFTRKAKPNTFRRFLESSQRYIDRVVEEADLRERGEILDLDAYLLLRRENSAVRLCFDL
IPYCLGIDLPNEVVEEPTFQKAYYASVDMVCWANDLYSYNMELNQGLEGNNFLTVLIQKQGMGIQRASDFVGEHYKRLMDDFLDASAHMPSFGP
AVDKDVQRYLQACTHWPAGNLAWSFETPRYFGARRDEIKRTRIVPLKPLELEKLEED*
//
>AvSTS_12
MALSQFSTLVFNAFSPTRDVSPVSSLPPTNSTRTVSPTPTVVEPGSFILPDLVSHCTVPLIYHSNGDAVAKHSLDWMLSFVPHFTPRKIAASRG
LLAGELTAFCYSNCSSERLRVVSDFMNFLFHLDNISDGMMARDAKGLSDWVMNAFEWPEGFRPLPGQKGEIEEISAAKLARDFWSRCIVDCPPA
VQQRFKSSMNMFFQAVHQQASDRANGNIPDLESYIDVRRDTSGCKPVFDLIEYTHGFALPDAVVEHPTMVALNQGANDLVTWSNDIFSYNVEQA
RGDTHNMICIFMALEGATLQEAVDRVGSLCKQTIDAFVEKQRRLPSWGEEIDRMVDLYVFGLQEWIVGALHWSYQTQRYFGADGEEVKRTRVVN
ILPLEKA*
//
>AvSTS_13
MALSQLWNLVFNAFSPTRDVSPVSSLPPTNSTRTVSPTPTVVEPGSFILPDLVSHCTFPLIYHSNGDAVAKHSLDWMLSFVPHFTPRKIAASRG
LLAGELTAFCYNNCSSERLRVVSDFMNFLFHLDNISDGMMAKDAKGLSDWVMNAFEWPEGFRPLPGQKGEIEEISAAKLARDFWSRCIVDCPPA
VQQRFKSSMNMFFQAVHQQASDRANGNIPDLESYIDVRRDTSGCKPVFDLIEYTHGFALPDAVVEHPTMVALNQGANDLVTWSNDIFSYNVEQA
RGDTHNMICIFMALEGATLQEAVDRVGSLCKQTIDAFVEKQRRLPSWGEEIDRMVDLYVFGLQEWIVGALHWSYQTQRYFGADGEEVKRTRVVN
ILPLENSA*
//
>LnSTS_01
MSPSSEIYFTIPNTLQNWPWPRHINPNYNVCKAESSAWCEGFKAFTPKAQKAFNKCDFNLLASLAYPLLNKEGCRVGCDLMNLFFVIDEYSDVA
DQCGAREQADIVMDALRHPLKPRPEGEWVGGEIARQFWENAIRTATPSAQRRFIRMFDSYLNSVVQQAEDRTHNYIRDIQSYFDVRRDTIGAKP
SFAINEIHLNISDEVMEDPIIKTLTSTSIDMLIIGNDLCSYNVEQARGDDGHNLVTIVMNERKIGLHAALQWISDLHDRLAAEFLEAYKKLPSP
IDPDVATYVDGLGNWVRGNDVWSFESERYFGVRGLEIQNRRIVQLLPKEKGEQGNTGLIPSTQGISVSTIKPSGFIQNILSSIWIKWGVTFLVL
MLSVASLRSIIILHRYELF*
//
>LnSTS_02
MADVIVLPDTLRNWPWPRSINPFYEVCKAESSKWCEGFRAFSPSAQRAFNKCDFNLLASLAYPLLNRDGCRIGCDLMNLFFVIDEHSDVADMKT
ARSQANIIMDALRNPLKPRPKGEWIGGEVARQFWENAIRSTTPTAQSRFVETFQSYTDAVVEQAKDRHTNHIRDIGSYMDVRRDTIGAKPSLAI
CQVHMNIPSEILEHPIIAELTRLCIDMLIIGNDLCSYNVEQARGDDGHNLVAIVCHEMKFSLERSFHWISVLHDDLVERFLRVWRDIPTFGGPI
DREVRTYVDGLGNWVRANDQWSFESERYFGKMGLEIMQTRRVKLLPKQHCRDVNLPLTERSLEKQLLEKRPKGPIAKVITLFFFILIVFSAGIQ
CYFVL*
//
>LnSTS_03
MSTQFVLPDILARWPWPRAINPYYAECRLSSQEWGAKFQAFTPKAQYAFDLCDPSLLASLGFPRTSRAGCRVVCDLMYLFGVFDEHSDVMDARS
VRRCADVVMDALRNPQVPRPKDEEIIGEITRSFWCNAIKTVGPTAQRRFIEEFSVYTESVVEQAQDRDHFVFRDIKSYMDIRRNNVGAKPAFAL
LEMDMELPHEIFDHKSLITLRECTVDMLCLANDMYSFNVEQAKGDDHNIITLIMLHEKLNIQQAMEYVAALHQIFADKFLGTYNSLPSFGSPVV
DAMVHRYVQGLGNWIRANDSWSFESWRYFKDEGLKIQKERVVKLLPKTKSLGRSNCAHPELPRDI*
//
>LnSTS_04
MALITSLKSTERTTFVLPDLLANWPFNPEPNPRQDIVAQSAAWVESFNPFDAKAQNAFNRCKFGIFASLAYPHATGAHFRVACDLMNLFFVFDE
YSDRADGEAVGKQAADIMNALRYPDTIPPEGDSLLGAMTRDFWLRTMKCASESSAQRFIRNFDEYTDAVRQEAADRDAGCIRSVPEYFKIRRGT
IGVHPSFDYYLLRDDLPDECVNHPDVQRLASAAVDMTILANDVYSYNKEQAKGEDSHNLVAVVMKEHDLTVQEAMDYIGDLYNHIRKQYCEKFQ
DLPRFNDDVDGLVREFCYGTGIWVTTNIKWSFASERYFGKEGMEIMKHRTVTLLPKVDKLFK*
//
>LnSTS_07
MSFHLPDIHRDWPFPRADNPHYVEVASESVRWIESFKLFPTDRQRSFHAILPGLLGSMAYPNLSRAHFRTACDLMNILFAVDDISDRLNPTEAK
ILADAMLDALRNPEKERDVTEHRLADLVRSFWARALETASSTTIRRFIRSYEDYVCAMTQEAQDRQYKSVRKSMDEYLDLRRYTGAIKPSLDLI
LLPLEISDKLLDSPTVKELEMIAIELIAVANDIVSFNVEQARGDIHNLVIVLMDGDKKMTVQTAMDLVGQWYRKRGQDFVEGLKRLPSVDDGNY
EILCLHQYAWGLGNWVTANYEWSFESHRFFGEENGEVMKYRVVKLLPKSPPLVTV*
//
>LnSTS_08
MLTSSHPTSFMLPDLVAHCTFPLSYHPNGDDIAQQSVAWLDSNCPDLTLKQRRALRGLQAGELTAYCYNTTSPERLRVVSDFMNYLFHLDNISD
GMMTRETDVLSDVVMNALWFSDHYRPTHGPGKEQPDEELNPGKLARDFWARCIPDAGPGVQARFKETLELFFEAVNIQARARDDGIIPDLESYI
DVRRDTSGCKPCWALIEYALDIDLPDYVVEHPIIEALNQGTNDLVTWSNDIFSYNVEQSRGDTHNMIVILMKYHGHDLQSAIDYVGNLCAQTID
AFNENKSRLPSWGAEVDEMVGRYLKGLQDWIVGSLHWSFMTHRYFGTNGAEVKQHRLVKLLPREQ*
//
>LnSTS_09
MNHTTTQILLPDLISMLPLEGATNPHYEKGAAESRAWINSYNVFTDRKRAFFVLGSNELLVSHAYPYAGYDVFKICCDFVNVLFVFDELSDEQD
GKDALTLGNIFVNAMTDPLWNDQSKFSRMTKEFRSRYTKLAGPNTTARFLKYWESYCAAVITEAELREKGQILDVDSFMELRRDNSAVRLCFGL
IEFCFGTDLPDEVFEDPTFLKIYWAAVDLVCWANDVYSYDMEQSKGISGNNIVTVLMHDKNMDLQTAVDYVGTYSKELVDRFMDLQAHLPSWGS
AVDSQVALFIKGLGYWVKGNLDWSFETQRYFGPKHMEIKNTLLVTLRPLECPEETDSESDTE*

//
>LnSTS_10
MSASTSTTTKIILPDLVSHCTFKKRVNRHRKQITTETKQWLFKGGNLNGKKRDAFHGLKAGLLTAMCYPDAGCPQLRVCNDFLTYLFHLDNLSD
DMDNHGTRSTADEVLNSLYHPHTYRSSARVGKMTRDYYKRLILTASPGAQQRFIETFDFFFQSVTQQASDRKDGLIPDLESYIALRRDTSGCKP
CWALIEYANNLDIPDEVMEHPIIQSLGEAANDLVTWSNDIFSYNVEQSKGDTHNMIPVVMNEEGLDLQAAVDFVGNMCKQSIDRFVDDRANLPS
WGPKIDRDVALYVDGLADWIVGSLHWSFESERYFGKTGREVKTTRVVNLLPRRV*
//
>LnSTS_18
MSPARIKTLPDVPECSEIRDCVADFLGRCKIGYKHAPIDPSFLECCYREAKSRGYVTEDEQSFLQFLPGGAAMALTGYAHLPDMSIRVSIALYT
ACGIYLDDKFKTDVDAVAKFNERFIRGTPQEDRVLDAFADIILDILHRFPRVSGNMIVSSTLNAVNALLLEYETRNMKVSNNADSYPTFSRIMS
GASESYALFVFPHTIPVEDYIQALPELMIFINNGNDILSFFKEEADGESVNRISLLAACRGSTKAEALRCLVNEAVTANDKVLKILEPHCDSYD
AYRRFSSGFIEFHATLKRYRLDELEL*
//
>LnSTS_19
MALYSLRSLLPTLFSFAISPISPLDSSMNYHTYRLSDEEIERSIYRDFLRKMEYCKPDLHDAEKTLEQALRSEMYSRNMQCPQLEKTVHLAARL
VELAYPDCTFEEQKVIALVNWFVIYLDDVDSEPCAAFVPRLLRGQKQLDPVLDAFADVLNSMHDYYDSLAANQIVTSILNFINLTPVESQIAAG
TFTVPSRSRRFPGFLRDRSGLGVPFALFAFPKSQGLDTAAYLRALPDMDFWICAANDLLSYHKEMLAGETNNYVSSRALVEKKTPLRVLVELER
ELEESRNVIHATLSSHPAAIKAWRAFEVGGVAWHLEQKRYKLKDLALDQQVTARG*
//
>LnSTS_20
MAPVSLIRPTSANVVQDLNKTGILRRFLQGMAYCPSNISGTNEELEKIMRAEMNNRNIRCPQLEKTLHLAASLIELGYHDCTLTEKTNIALYNW
YLIYIDDMSSKDTGPFMAFQERFLRRMPQLNPVLEALVGVLMRIYELYDMPTANSILSATFNFVNSTCIEPEIETLPLTRGLVRFPWFLRDQTG
VAIAFALLLFPKSKNVGVTDYIQALSDMNFWISVTNDILSFHKEELAGERANYVYNRAYIEDKVPLSVLAEMSQELFESRNAVYAALEHCPQAA
DVWHTWEQGYVRWHIDQKRYKLSELSL*
//
>LnSTS_21
MDFSTPNINGTNTKLEAAMTEEMDTRNLRCYQLEKTLHLAASSTELCYHTCDFEEKKSIALYIWYMFYVDDMASRDVDSFAPFQQRFLRRSPQL
DPVLTAFADLLLRMCDQYDTITANSIISATFEFVNSTCIESDLERMPLVQGVHRFPLFIRDRTGVSTSFGLMLFPLSKKIRVTDYIQALPDMIF
WISLSNDLLSFHKEELAGDTNNYVHIRALMEEKTPIEVLFEMEQELTLARQTIYKALAGSPTATAAWLGFERGYVASHLAQKRYKLQDRGL*
//
>LnSTS_25
MSQDTSKAKFEMAAVLKEFLQETAYHPPTIPSTNEELENKMYEYMHGRNLACPQLERTLHLAASLIEIAFPECTLSEKTVIAIFNWFIIYIDDT
SPGDVSPFVAFGPRFFSQTQQLDPVLDAFVDILQRICDQYEPATANYILASAFNYISSTCIEPEIEAHAVVPGVIRFPWFIRGQTGISLAFALM
LFPKSKGVSAVQYIQVLEEMNFWISGVNDLMSFPKEQLAGERNNYVHVRARTEEKSPMEVLAELNQELHMSRKLIYAALSLIPGASTIWSAFEN
GYIKWHLIQERYKLAEMDLTG*
//
>LnSTS_27
MSTVTQLLNNFVHVVSRAGSLITPDFLNGEFYDAPLSDAGDPPAKDDVTDLMVTVGQILGKMLHDCHIPYCAVSFDYGLMKAPVAEAERRGYAL
EGPNSMRQALFLGVYLTSTMYAHVTDDDALRTYIIFYIMFMFYVDDKYFEQSDKESGLLHFIARFNQNQPQAEPSLTNFADFLRDDTAAVFEPI
GGGIIITSTLNFINGMILETSVKLGQISPYAQNFPYFVRNKSGFPEACVILAFPRDMPVRQYAQAFPEICDYLCYVNDIMSFYKEELVGETENL
VSLLAAVTDSKVEHLLSTDSPAPVTRNDKYGVLRRLSAAVASGHKKTLQILSGNPVAQDIYLKFAIQYVTMYISMRERYKLDELRFGDKA*
//
>PoSTS_01
MSAIASSSAQAAPPSKIVIPDLVSHCTFELRVNRHRKIASAQSKRWLFRGDNLTGKKRDAYHGLKAGLLTSMCYPNAGAPQLRVCCDFMNYLFH
LDNLSDDMDNRGTKSTADVVLNALYNPKMRQTKRVGKMTKDYWQRLIRTAAPGAQQRFIETFDFFFQAVSQQARDRAEGSIPDLESYIALRRDT
SGCKPCWALIEYANNLDIPDEVMDHPIIRSLGEAANDLVTWSNDIFSYNVEQSKGDTHNMIPVVMHEEGLELQAAVDFVGALCKQSIDRFVECR
ANLPCWGPEIDRQVAVYVEGLADWIVGSLHWSFESERYFGKSGLEVKKNRVINLLPRRS*
//
>PoSTS_02
MSTLISPDHFILPDLVSDCTYPLRVNDNCEEVARVSEQWLLNAANHNERKRRAFLGLKAGELTAACYPDADAFHLRVCVDFMNYLFNLDDWLDE
FDVEGTRGMHDCCIGVMRDPLNFETDKRAGIMTKSFFSRFIETGGPGCTERFIHTMDLFFKAVAIQAADREKDVIPDFESYITIRRDTSGCKPC
FALIEYASRIDLPDEVAEHPLIRSMEEATNDLVTWSNDLFSYNVEQSRGDTHNMIPVIMHQRDLNLQEAVEYVGALCKSSIQRFETDRKNLPTW
GPEIDRDVAVYVEGLQNWIVGSLHWSFDSERYFGTSGLEVKQQRIVKLLPKVPS*
//
>PoSTS_03
MVPVASFIQEPSRAPSSFILPDLVSHCKFPLTYHPQGDAVALESVTWLDRLCPDLSPKARKALWGLQAGELTAYCYPSCSPDRLRVVSDFMNYL
FHLDNISDGMMTRETDVLADSVMNALWHPEEYRPTRSPGKEQPAEELDAGKIARDYWSRCIPGAGLGVQARFKESLQLFFEAVNVQARARDAGE
VPDLESYIDVRRDTSGCKPVFDLIEYAMDFELPEEVVNHPVIKALNQGTNDLVTWSNDIFSYNVEQARGDTHNMIVILMKYHGHTLQSAVDYVG
ELCCQTINNFCANRQVIPSWGPETDRMVQEYVQGLQDWITGSLHWSFKTHRYFGTNGAEVKKTRLVKLLPLKNGANPCAGEATK*
//
>PoSTS_05
MSSQPTQIVLPDLLAMCPLKGYTNPHYKEAAAESVAWIDSYNVFTDRKRAFFVQGCNELLVSHTFPYAPYEQFRTCCDLVNLLFVIDEVSDDQS
GKDARATGQVFLNALGDPEWNDGSLLSRITKDFRARYFRLAGPNSSRRFLKHCEDYINAVSTEAELRERGEVLDIEPFTALRRENSAIRLCFGL
FEYALGIDLPDEVFQDKTFQDMYFAAVDMVSWSNDVYSYNMEQAKGHSGNNIVTVLMKSKGMGIQEAVDHVGVHFQQIMDIYMDSKTRLPSWGS
EVDVNIARYVEALGHWAKGNLDWSFETQRYFGAEHLEVMATRVVTLRPHGSPEDFDE*

//
>PoSTS_06
MTSTTKHPLPSHFILPDLLDQWPFETEPNPHQEIVDDSARWVESYKAFSPKAQDAFNRCNFGIFASLAYPRSEGTHYRAACDLMNLFFVFDEFS
DAENGDVVRQQAADIMNALRYPDKIPAGGDSILGAMTRDYWKRTLEVSSKSSAERFIRNFDGYTDAVRQEAVDRDEGRERSIEEYMELRRGTIG
VYPSFDYFLLEDDIPDEYIDHPAVASLALGAVDMTILANDVYSWNVEQCRGEDRHNLVAVAMREKGLSVQEAMDYVGTIYAGIRDKYVKEFNEL
PQFPEKYDKLVKDYCWHMGNWVTTNIKWSHFGERYFGKKGREILKHRTVEVMRPSVLGWVMRKSYPLIVTVKYAPREYYYIAVTFLMLLLALLS
SPSRARILGHSSFPSSF*
//
>PoSTS_09
MNRGASYQLPDLTSLVRDFELHTNPHCHAISLTSGPGKLIDERDKACWRGMKAGLLASLCFPICDAPQLKDLADIMSAFICIQVRLVGACDMAE
CGWTSSEGEGQAVSGGWELLAQNALLARLIPQVKRLAARAPADWSSRLEKASHAFHAAHLQLVAKSPPPDSIEACLALHTDASGFRIIFLLAEV
IKELHLPPNVELLEQLADSALKIIVGFLQLVSYNLHASKKSSGNAVAVVMSLREISLQGALKVCMGECRRNVETYRTVERAILVGLSGEAPPSM
PPRRPRPLAGHTRASSSIWSWIPFVAPQEAPAPPAPEPAPLLPLLEVEEWDDEVKDDVRSYVNGLRDCVVGYIHWLYETEIYFGQLGDEVRATG
WTFLPVVSS*
//
>PoSTS_11
MASHYRLPNLLALFNTDGARVNPRFHELDSQFNQWLDTLTLDAGFVETLKQIQMPVLISHAYPEASLGQLRTCLDYLTLAFIFEEITENTSSSQ
SQRWADLYMGMYRGTASVLSNIVPEETDHPLFPVMTSLAHNVMSSLEPVFHESFITENLASVQAIVQEAIDREVDDAPSRQPRTLEAYYINRLA
TVGLMPFLILAQWTGGIRLPAFIQDSHSVQTMSQAAVEMVFLANDIYSYKKEKMAGATQNNVITVIIEDPSTSICEGNLQGSFDYSERLFQDAL
SRFYTHREMLLENVSDQQNYTADINKFSRAMMDCVVGNIKWSMICRRYAVFECEQARNNEVVKI*
//
>PoSTS_16
MPVNIRDIVALHLRRCNIPYEVPVPDVKLQAACLNYAKNHGYEIGGKKTLGPCIPGGVVMASNAFGHIADLPTRVIIGIYTAFMIYLDDISSSD
IEAVAQFNQRFYRAEPQLDKVLDDFAQLLRDFPNYFCTAGSDMIVTSTLNFVTSLFMDVETEGMDVDGNAKRYPRFARILNGAALAYTLLVFPK
SLPISSYIQAVPEINVYVENTNDVLSFYKEQMAGDDINYASLLSHSYSVTKYEALIHLSNIAVDANERILRLLEPVPEALEAYTRFRNGYVRFH
TGLGGRYKLAELKLTNISDYQDRLGAHVNQTACKVCQRNLCN*
//
>TvSTS_01
MAVIAAAPRPKRIFFPDLIAHCPYPLRYNPHCEAASAESKVWLMSGCRLSKKKRAAFHGLKGGLLTAMVYPEADYDQFRVCCDWINYLFHLDNI
CDEMDDRTTVSTAGVIIGALRDPHGFRPASAVGRLTQSFWRRMSATASPGAQRRFIETFELFFRAVAQQARDRASGNIPDLESYIAMRRDTSGC
KPCWALIEYANDLDLPDWVMDHPCVRGLEEAANDLVTWSNDIFSYNVEQSNGDTHNMIVVVQTQEQLDLQSAVDYVGDLCLGCVDRFETLRAAL
PSWGPQIDDQLAVYVQGLGDWMIGNLVWSFETERYFGRSGRKVRRALAVALLPRRK*
//
>TvSTS_04
MPVILAFPDFISPVPYPLRCHAQEREVSRESEEWLLSMANFSEKQRAKFLTLNAGLLSGMCYIDCSFDELRVCTDFMNFLFTLDDWTDEFDVTG
TRGLAECVMNTLYWPHTYSSDTAAHRLTKSFWVRMQETAGPGCQQRLMSTLDTYFQAIMQQADDRGSENIPELEEYILLRRDTSGCKTGFAFIE
YAARIDLPDDVVDHPIIKALADATNDLVSWANDVLSYNAEQARGDTHNLVCVLMHMNGVDRQDAIELAGELWTKTLEWFFECRKVVPSWGAEID
RQVALYIQGLEDWIIANAEWSFETERYFGKDGHAVKKSRKVALLPQRVRA*
//
>TvSTS_05
MSSPSSFVLPDLHAVTPFKGSFNPHYPEAAAESSEWVNSYKVLSDKKRAFFLQGGSELLCAHAYPYAGYQQFRTTCDFVNLLFTVDEISDDQNG
KGAYETGLTFYNAMSNPAYDDGTVLCKMTKEFTARLLEHCGPQTYRRFIKHCKDYIEAVAVEADLRERGEVLDLEAYQTLRRENSAVRFCFGLA
GYALGIDLPDEVVEHPAFMAMHLSTVDMVCWSNDLYSYNMEQAMGHTGNNVITVLMQHKGLDLQGAADYTGVHFKGLIDTFLDAKRSLPSWGPK
LDGEVAQYAMAMETWVIGNLNWSFETQRYFGHARHEIKRTRVVQLYPRRIVEESSDEEDN*
//
>TvSTS_06
MSAQQFTLPDLLAVCPLKDATNPHYAQAAAESTAWVKSYNIFDARKLAFLLQGSSELLVSHAYPYAPYEQFRTCCDFVNLLFVVDEVSDDQNGK
DARRTGEVYLNVMRYPDWDDGSALAKMTREFKQRLLAFAGPNSYRRFLMHCDDYVNAVAREAEYRERGEVLDLDAFQTLRRENSAIRLCFGLFE
FALGIDLPDFVFQDPHFMTLYWAAADMVCWSNDVYSYNMEQAKGHTGNNIVTVLMRQKSIGLQEAADLVGAHFAALMGRFVETKKQLPSFGAAA
LDDAVAKYVAAMEHWVIGNLEWSFESQRYFGAEHTRVKATRVVVLSPADEN*
//
>TvSTS_07
MSRSLRLPDTLSRWPYPRRINPAYEEVSAESAAWLRSFHAFSDQAQVAFDKCKFGLLASLTYPNVDKDHLRAACDLMNVFFVFDEQTDIADTTR
TRELADIVIDAVRHPDRPRPEGEPIVGEITRQFWAHACVNSTASGRARFEQEWTRYVESVVGQAEDRDAGRLRTTEEYLELRRFTIGADPSYAL
AMARVDLPLAVSELPVFRKLRGCITDMLIFDNDLLSYRKEYAAGDDMHNIITLVMNEKQIDVDAAVEWLAAEHAKRVDEFFVLWPQASAMSFGS
DELNQAVATYLDHLVNWPRGDECWSFESGRYFGKDGARVKKERVIELKTRD*
//
>TvSTS_09
MPQQYIHIPDLMATWPWPDRIHPLHEEVDAESSAWLKSFAPFTPQSQHAFDKCNAGLLCAHVYPDISRKALRTSMDLINILFVVDEYTDVESAP
AVREIADMLVDAVYNPQKPGQAGELVLGKMMREWSRRCLPAATPEAVSHFRKSFTDYLEAVVSEAEGRCTGTIRSIDSYMKNRRENNGVRPSLF
PCELHLSIPDEVFYHPHVVDLQTCIVDMITTVNDIISYNREQATHIDDCNLLTVVMRELGTNFDGAMAWIVDYHHDVATRFIEGLKNIPSFGPE
ADARLQEYLQSIASWPGGADSWSFESARYFGERGAEYQKSRRVPLLQKSKRYPQCRREKVHVHLIEHLAQPTS*
//
>TvSTS_10
MATWPWPRRIHPLYEEVAAESSAWLKSFAPFTPESQHAFDKCNFGLLAALAYPDASRETLRTSIDLMDLFFVIDEYTDHELAPAVKEMVGMLLD

```
AVHNPQKPRPAGEPILSEMMREWSERCLPTATPEAASHFLKSFTDYLEAVVAQADERCSGAVRSIDSFIKIRRENNGGRPSLFPSELHLSIPDE
VFYHPYVVDMQACIIDMITTINDILSYNREQATHTDDYNLVTVVMRELGMDLDGAIEWVVDFHHGIAARFIEGLEHLPSFGPGVDAQLQEYLQN
IANWPRCVDSWSFEAGRYFGERGVEYQRTRQVPLLPKSKRDVLYHREEVQVHLIEYLAQPTPA*
//
>TvSTS_12
MSVEQLRPFPTHFRLKDLTAITSPVFEFKTNPHQEAAAQATNVWFERRNVYHGLKKQKFLSHRFDSYAGMSFPDADAAHLETCIAFFFWAFSFD
DLSDEGALQSKPDAVQVGVDISMEVLKNPAAPAPNFPYAAMLHDIWRRFRSTASPGACNRFYRAVESWMNSQVEQARNRSTDEIPSVEDFIILR
RRTIGGPIVEAMVEYSLNLQIPEYVWDHPVLQEMSKAVIDIMTWPNDLCSFNKEQADGDFQNLVFCIMIERDVDLQRAVDILTDMLAQRVADYV
RYRDQLPSFGPEVDAELARYNKAMEQYTQGTVVWYYYSPRYFRGQDVSGKTEIVVPVYERAAPVEEAAPAPHVSTTDSEKGAVYASFRFPFSAF
PHLSLLLLSLFACLLYVNLPSLGLRLALP*
//
>TvSTS_13
MGSIRIPNFPAFCNPYLELRSNPHCHAISLSAEKWALSTPLFLDAEEEARLPGARLGLLAALCFPTCDASQLFAITKFLIVIMHWVDRPRSLYA
DEEAFDPVWAKYFRPTTRREWQKRFQRHLAAFRLGQALTAQDAAQGIVPDLESYIGLRRDACGVRMLLDLVLYAGGLNVPPYVYEHPVLRRLRQ
DAADIIAWSTDVAAYARRPHSSNIIAVLMHERRYTAQAAAHFAGNLVKDTITNFVTNEMSLPAFGDRDADVRAYVQGLRDCVVGTLHWLYETDR
YFGETAEDVRSSGWVFMNA*
//
>TvSTS_14
MPYIQDACSSPLPLCASVDAITEIKDILKDFLRRLNYRSPHTPANAKLRAEVTAEIASWNADLSPSFKHGLAETCYTIAESAYAHTSYEHQRII
ALYTTYLTYVDDLGGRDLDALGEFGRRLLAREALGDSALDRLVTNLQDMYAYYPRLSAHSIAVSTLDFFVGSYVEATGKEMAVAPGATKYPGYM
RMKTGIGAAYALFNFVKDWRDPADHFFLQLIPEIEFYTDTINDILSFYKESLAGETDNLIHLRAAAEQKDPLSVLRDVVEETLESIRKVEVLTA
ADPQLAKICRSYVVGYVEFHFRAKRYRLEDLEMEY*
//
>TvSTS_16
MVAEVLPSPANPFLVYANGVSPILPTSLSHIDGHVYRSGSNDSNGLSRLLVTGPQGLPPAITPEAIQEMKAIMQDFLQQCNYEDPSTPKDIELR
ERMALEMAAWSLDLPATFIEQVHETSCHFIETAYVHTTLEHRFFVARYCAYFLYADDLPGRCLDALIQFPRRFANREQQLDPILNRLAEMIRGA
HELWTDVGASAIIAGTLDALTAFYIEYTTCGLAVKPQAVRYPYYLRLKSGIDPPFVAFIFMRGWRDTAESYVQMLPEIEYWIGATNDLLSFYKE
ELAQETNNYIHIRAAMEQTTGLVILRKLADEILDTTRRIEGLAAEDAELAALWHGYKQGFLEFQIKAPRYRLSDLGLTE*
//
```

References

Agger, S., Lopez-Gallego, F., Schmidt-Dannert, C. (2009) Diversity of sesquiterpene synthases in the basidiomycete *Coprinus cinereus*. *Mol Microbiol* **72**: 1181-1195.

Barfield, M., Chakrabarti, B. (1969) Long-range proton spin-spin coupling. *Chem Rev* **69**: 757-778.

Dexter, D.D., Silverton, J.V. (1968) The conformation of from 1,4-cyclohexadiene stereoisomeric allylic-allylic proton couplings. *J Am Chem Soc* **90**: 3590-3592.

Felsenstein, J. (1989) PHYLIP—phylogeny inference package (version 3.2). *Cladistics* **5**: 164-166.

Ichinose, H., Kitaoka, T. (2018) Insight into metabolic diversity of the brown-rot basidiomycete *Postia placenta* responsible for sesquiterpene biosynthesis: semi-comprehensive screening of cytochrome P450 monooxygenase involved in protoilludene metabolism. *Microb Biotechnol* **11**: 952-965.

Ichinose, H., Ukeba, S., Kitaoka, T. (2022) Latent potentials of the white-rot basidiomycete *Phanerochaete chrysosporium* responsible for sesquiterpene metabolism: CYP5158A1 and CYP5144C8 decorate (*E*)-α-bisabolene. *Enzyme Microb Technol* **158**: 110037.

Thompson, J.D., Higgins, D.G., Gibson, T.J. (1994) CLUSTAL W: improving the sensitivity of progressive multiple sequence alignment through sequence weighting, position-specific gap penalties and weight matrix choice. *Nucleic Acids Res* **22**: 4673-4680.

Wawrzyn, G.T., Quin, M.B., Choudhary, S., López-Gallego, F., Schmidt-Dannert, C. (2012) Draft genome of *Omphalotus olearius* provides a predictive framework for sesquiterpenoid natural product biosynthesis in Basidiomycota. *Chem Biol* **19**: 772-783.
